# Non-coding RNAs in Various Stages of Liver Disease Leading to Hepatocellular Carcinoma: Differential Expression of miRNAs, piRNAs, lncRNAs, circRNAs, and sno/mt-RNAs

**DOI:** 10.1038/s41598-018-26360-1

**Published:** 2018-05-22

**Authors:** Srinivas V. Koduru, Ashley N. Leberfinger, Yuka I. Kawasawa, Milind Mahajan, Niraj J. Gusani, Arun J. Sanyal, Dino J. Ravnic

**Affiliations:** 1 Division of Plastic Surgery, Department of Surgery, Pennsylvania State University College of Medicine, 500 University Drive, Hershey, PA 17033 USA; 2 Department of Pharmacology, Department of Biochemistry & Molecular Biology, and Institute for Personalized Medicine, Pennsylvania State University College of Medicine, 500 University Drive, Hershey, PA 17033 USA; 3 Genomics Facility, Department of Genetics and Genomics Sciences, Icahn School of Medicine, Mount Sinai, 1425 Madison Ave, New York, NY 10029 USA; 4 Program for Liver, Pancreas, & Foregut Tumors, Department of Surgery, Pennsylvania State University College of Medicine, 500 University Drive, Hershey, PA 17033 USA; 5 Division of Gastroenterology, Hepatology and Nutrition, Department of Internal Medicine, Virginia Commonwealth University, 1201 E Marshall St, Richmond, VA 23298 USA

## Abstract

Hepatocellular carcinoma (HCC) was the fifth leading cause of cancer death in men and eighth leading cause of death in women in the United States in 2017. In our study, we sought to identify sncRNAs in various stages of development of HCC. We obtained publicly available small RNA-seq data derived from patients with cirrhosis (n = 14), low-grade dysplastic nodules (LGDN, n = 9), high grade dysplastic nodules (HGDN, n = 6), early hepatocellular carcinoma (eHCC, n = 6), and advanced hepatocellular carcinoma (HCC, n = 20), along with healthy liver tissue samples (n = 9). All samples were analyzed for various types of non-coding RNAs using PartekFlow software. We remapped small RNA-seq to miRBase to obtain differential expressions of miRNAs and found 87 in cirrhosis, 106 in LGDN, 59 in HGDN, 80 in eHCC, and 133 in HCC. Pathway analysis of miRNAs obtained from diseased samples compared to normal samples showed signaling pathways in the microRNA dependent EMT, CD44, and others. Additionally, we analyzed the data sets for piRNAs, lncRNAs, circRNAs, and sno/mt-RNAs. We validated the in silico data using human HCC samples with NanoString miRNA global expression. Our results suggest that publically available data is a valuable resource for sncRNA identification in HCC progression (FDR set to <0.05 for all samples) and that a data mining approach is useful for biomarker development.

## Introduction

Hepatocellular carcinoma (HCC) was the fifth leading cause of cancer death in men and eighth leading cause of death in women in the United States in 2017. The incidence of HCC has been increasing every year, with an estimated 40,710 new cases in 2017^1^. In addition, death rates are increasing by 3% every year, with an estimated 28,920 deaths in 2017^1^. These statistics suggest more research needs to be done towards understanding the biology, diagnosis, and prevention. Primary liver cancer, or HCC can be triggered by ongoing inflammation from pathologies such as cirrhosis, dysplastic nodule formation, viral infections (i.e. viral hepatitis), and NASH. Cirrhosis is a condition in which the liver becomes scarred secondary to repeated insults.

HCC often originates in a focus of dysplasia within a cirrhotic regenerative nodule. With progression to more severe dysplasia, the lesion assumes increasingly malignant characteristics until it becomes frankly cancerous. Tumors may be well differentiated or poorly differentiated. As expected, poorly differentiated HCC has a more aggressive course and poorer outcomes than well differentiated HCC.

Scientific progress in next generation sequencing (NGS) has enhanced our understanding of biological systems by profiling whole transcriptomic expression at a molecular level^2^. Small RNAs are small non-coding RNAs (sncRNA) consisting of 17–250 nucleotides in length that perhaps play a crucial role in disease development^3^. A nearly comprehensive repertoire of various types of sncRNAs has been collected and analyzed including: microRNA (miRNAs, 17–22 nucleotides)^4^, piwi-interacting RNAs (piRNAs, 26–33 nucleotides)^5^, small nuclear/nucleolar RNAs (sn/snoRNAs, 70–120 nucleotides), long non-coding RNAs (lncRNAs, more than 200 nucleotides)^6^, and circular RNAs (circRNA)^7^. This has led to great interest in revealing their role in transcriptional regulation. piRNAs are the largest class of the small non-coding RNA family and are implicated in epigenetic and post-transcriptional regulation but still lack functional characterization^8^. lncRNAs are a diverse class of RNAs, believed to have an important role in cellular mechanisms; however, little biological relevance has been established thus far^9^. circRNAs are a recently rediscovered class of non-coding RNAs, which were initially described as scrambled exons^10,11^. They are resistant to endonuclease treatment and are highly stable^12^. snoRNAs are perhaps the most ancient and highly conserved class of sncRNAs carrying out a fundamental role in modification and processing of ribosomal RNAs (rRNA), transfer RNAs (tRNA), and small nuclear RNAs (snRNA)^13^. Two well-known classes of snoRNAs are C/D snoRNAs and box H/ACA snoRNAs which primarily differ in sequence and structure^13^.

The present study focused on in-depth analysis of small RNA sequencing data obtained from cirrhosis, low-grade dysplastic nodules (LGDN), high-grade dysplastic nodules (HGDN), early stage hepatocellular carcinoma (eHCC), and advanced stage hepatocellular carcinoma (HCC). Tissue samples were compared to healthy liver tissue. We aimed to identify the differential signature and dysregulated expression of small non-coding RNAs. We further identified the most predominantly expressed non-coding RNAs and molecular pathways that could serve as biomarkers during malignant transformation.

## Materials and Methods

### Read Alignment and Annotations

Healthy (BioProject: PRJNA266511) and diseased liver (BioProject: PRJEB11462) small RNA sequencing raw sample datasets^14^ were downloaded from NIH short read archive (SRA). The datasets contained 14 cirrhosis, 9 low-grade dysplastic nodules, 6 high-grade dysplastic nodules, 6 early HCC, and 20 advanced HCC samples along with 9 healthy liver tissue samples (normal clinical assessment and normal liver enzymes). Downloaded SRA files were converted to FASTQ files using the SRA toolkit version 2.5.7^15^. The FASTQ files were uploaded to PartekFlow^®^ software, version 6.0 (Partek, Inc., St. Louis, MO) on a Linux based High Performance Computing system at Pennsylvania State University College of Medicine, adapter-trimmed, and remapped to human genome hg19 using BWA-0.7.12 aligner (BWA-MEM) with a few modifications (mismatch penalty 2, gap open penalty 6, clipping penalty 4, and alignment score cutoff 15) for short read mapping^2,16^. miRBase version 20 (http://www.mirbase.org/), which contains more than 1900 high confidence miRNAs^17^ was used for annotation. piRNA data was generated and annotated from piRBase (http://regulatoryrna.org/database/piRNA), which is manually curated with a focus on functional analysis^18^. lncRNAs were quantified using reference annotation LNCipedia (http://www.lncipedia.org) version 3.1, downloaded from all coordinates relative to the hg19 reference genome^19^. circRNA database (http://www.circbase.org) contains thousands of circRNAs and annotation was download and quantified^20^. Total small RNA (including miRNA, piRNA, snRNA, snoRNA, mt-RNA, tRF3, tRF5, tRNA, and rRNA) was annotated using Gencode version 26 (www.gencodegenes.org)^21^, which provides comprehensive information on human sncRNAs. Transcript abundances were determined and expression levels were represented using normalized reads per million (RPM) values. All small RNAs with expression RPM values > 1 in 100% of the samples were considered robustly expressed and used for further analysis. Expression matrices were compared among clinicopathological features including cirrhosis, low-grade dysplastic nodules, high-grade dysplastic nodules, early stage HCC, and advanced stage HCC samples^2^. Statistical analyses were carried out using the non-parametric Mann-Whitney U test followed by false discovery rate (FDR) correction through the Benjamini-Hochberg method. A default FDR < 0.05 was considered statistically significant^22^ with a log_2_-fold change more than 1. Circos plots^23^ were generated for differential expression of all small RNAs in various stages of liver disease.

### HCC and Normal Liver Tissue Samples

Hepatocellular carcinoma (n = 3; moderately to poorly differentiated) and normal liver tissue (n = 3; adenoma) frozen samples were obtained from the Institute for Personalized Medicine (IPM) at Penn State College of Medicine, Hershey, PA, after written approval. Total RNA was extracted using a Direct-zol™ RNA Kit (Zymo Research, cat#: R2051) according to the manufacturer’s instructions. The extracted RNAs were quantified and quality checked using a NanoDrop 1000 Spectrophotometer (Thermo Fisher Scientific, Waltham, MA) and a BioAnalyzer RNA 6000 Nano Kit (Agilent Technologies, Santa Clara, CA).

### MicroRNA Profiling Using NanoString nCounter miRNA Assays

Total RNA samples were analyzed according to manufacturer’s instructions for the nCounter miRNA Expression Assay kit (NanoString Technologies®, Seattle, WA). Briefly, 100 ng of each sample total RNA was used for nCounter Human miRNA sample preparation. Hybridization was conducted for 16 h at 65°C. Subsequently, probes were purified and counted on the nCounter Prep Station. Each sample was scanned for 600 FOV (fields of view) on the nCounter Digital Analyzer. Data was extracted using the nCounter RCC Collector.

### NanoString nCounter miRNA Data Analysis

For platform validation using synthetic oligonucleotides, NanoString nCounter miRNA raw data was normalized for lane-to-lane variation with a dilution series of six spike-in positive controls using nSolver v4.0 software (www.nanostring.com/products/nSolver). The sum of the six positive controls for a given lane were divided by the average sum across lanes to yield a normalization factor, which was then multiplied by the raw counts in each lane to obtain normalized values. For each sample, the mean plus two times the standard deviation of the eight negative controls were subtracted from each miRNA count in that sample. Only miRNAs with non-negative counts across all samples were retained for downstream analysis. The relative miRNA levels were indicated as median fold changes and a cutoff of two fold-change (up or down) was used^24^. A Venn diagram was prepared using FunRich 3.1.3 open source software^25,26^.

### Biological Processes and Gene Network Visualization by MetaCore

Biological pathway interactions of microRNA expressions were analyzed using MetaCore pathway analysis of differentially expressed genes (Thomson Reuters, New York, NY)^2,16,27^ with *p < *0.05 and greater than two-fold change. We performed multiple comparative analysis and enrichment analysis on all five stages of liver disease. Functional gene networks were built based on differentially regulated miRNA gene lists as input to generate disease biomarkers and Gene Ontology terms (Data analyzed by Gene Arrays, Entity of Vedic Research, Inc., New York)^2,16,27^.

### Statistical Analysis

Paired student’s t test was used to compare diease stage vs healthy samples, with an FDR* < *0.05 considered statistically significant and log_2_-fold change greater than one. Furthermore, the Benjamini and Hochberg multiple testing adjustment method was applied for all small RNA sequencing studies and an *p-value* < 0.05 with fold change greater than two for pathway analysis.

### Ethics

Data presented in the study was downloaded from the NIH data sets (BioProjects: PRJNA266511 and PRJEB11462). We haven’t recruited any human subjects in this study (Not applicable).

## Results

### Differential Expression of miRNA in Cirrhosis, LGDN, HGDN, eHCC, and HCC Tissue Samples

**Figure 1:**
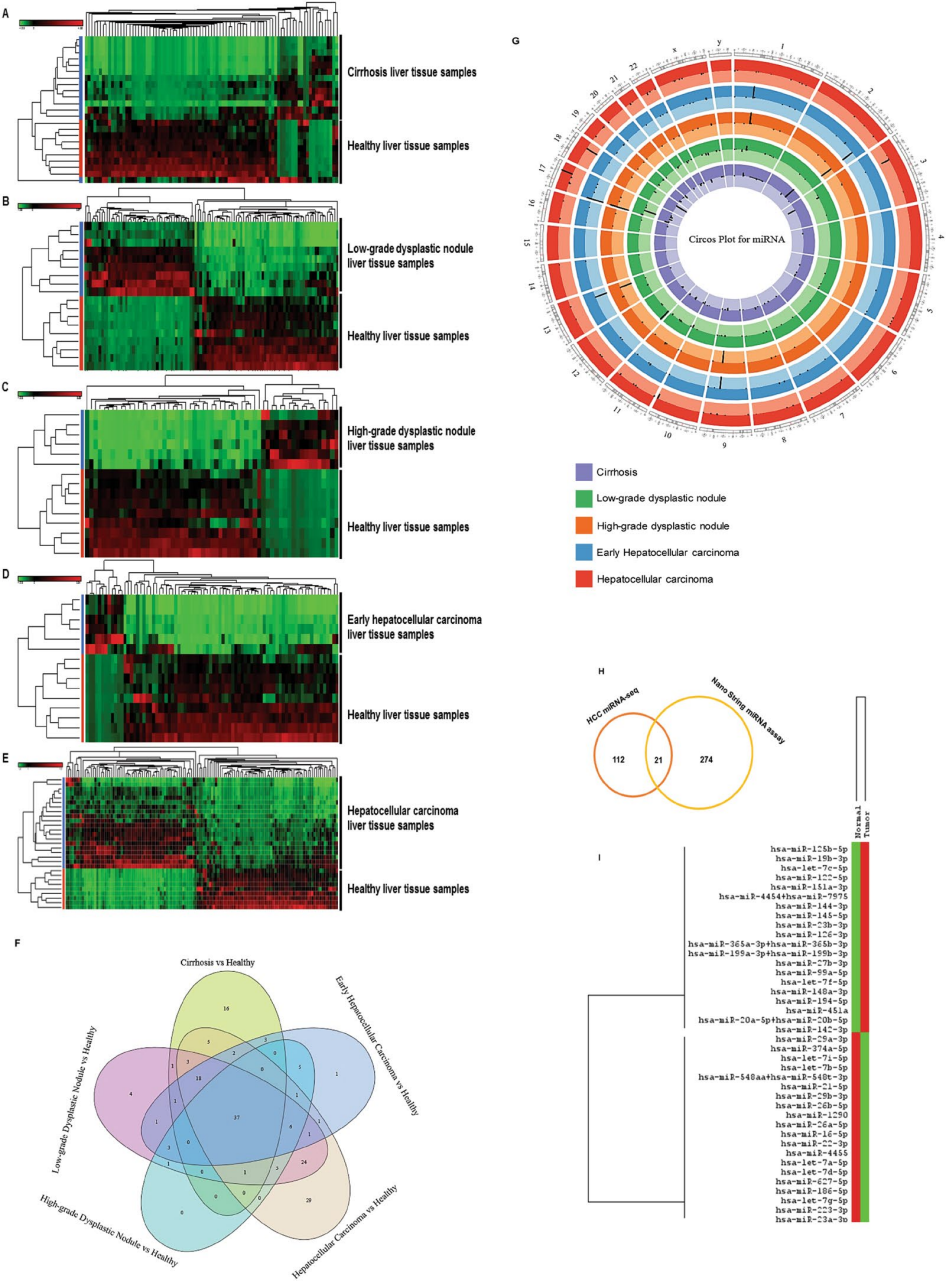
Differential expression of miRNAs in liver tissue samples: Differentially expressed miRNAs were quantified (*FDR* < *0.05*) and a heatmap was prepared for each disease stage (Cirrhosis, Low-grade dysplastic nodule, High-grade dysplastic nodule, Early stage Hepatocellular carcinoma, and Advanced stage Hepatocellular carcinoma) with healthy control samples (**A**–**E**). Enriched miRNAs for all stages of liver disease were summarized by a Venn diagram, which identified 37 miRNAs commonly expressed in all stages and 29 additional differentially expressed miRNAs were enriched in advanced hepatocellular carcinoma alone (**F**). (**G**) A Circos plot was prepared incorporating all differential expressions of miRNAs in cirrhosis, low-grade dysplastic nodule, high-grade dysplastic nodule, early hepatocellular carcinoma, and hepatocellular carcinoma tissue samples compared with healthy samples (FDR < 0.05). Chromosomes and bands were listed in the chromosomal positions of miRNAs affected expression in liver disease vs healthy samples. The innermost ring is for cirrhosis, followed by low-grade dysplastic nodule, high-grade dysplastic nodule, early hepatocellular carcinoma, and hepatocellular carcinoma. Darker and lighter background colors represent upregulated and downregulated genes respectively. We validated the miRNA sequence data using NanoString global miRNA expression assay. Data was exported using nSolver software. (**H**) A Venn diagram showing miRNA overlap between HCC miRNA-seq data and HCC-NanoString validation data. (**I**) A heat map illustrating the top 20 up- and down- regulated miRNAs in the HCC-NanoString validation data.

We first annotated the datasets to microRNA analysis. Using stringent statistics (FDR < 0.05) with filters set at a log_2_-fold change greater than one and total minimum reads > 500, expression data was visualized (Fig. 1A–E, Tables 1–5). Our analysis found 87 miRNAs differentially expressed in cirrhosis compared to normal liver tissue (15 upregulated and 72 downregulated; Fig. 1A, Table 1), 106 miRNAs in LGDN (46 upregulated and 60 downregulated; Fig. 1B, Table 2), 59 miRNAs in HGDN (18 upregulated and 41 downregulated; Fig. 1C, Table 3), 80 miRNAs in eHCC (12 upregulated and 68 downregulated; Fig. 1D, Table 4) and 133 miRNAs in HCC (64 upregulated and 69 downregulated; Fig. 1E, Table 5). The top five differentially upregulated miRNAs in cirrhosis (Table 1) were: miR-7704 (403-fold), miR-22 (143-fold), miR-101 (113-fold), miR-486 (75-fold), and miR-192 (32-fold). The top five downregulated were: miR-122 (312-fold), Let-7g (204-fold), miR-103a (83-fold), miR-532 (79-fold), and miR-451a (62-fold). The top five differentially upregulated miRNAs in LGDN (Table 2) were: miR-141 (625-fold), miR-101 (208-fold), miR-22 (111-fold), miR-16 (61-fold), and miR-486 (35-fold); whereas, the top five downregulated were: miR-451a (513-fold), miR-378c (104-fold), miR-361 (95-fold), miR-122 (81-fold), and miR-30c (78-fold). The top five differentially upregulated miRNAs in HGDN (Table 3) were: miR-101 (266-fold), miR-22 (170-fold), miR-16 (54-fold), miR-192 (45-fold), and miR-19b (34-fold). The top five downregulated were: miR-26b (1 million-fold), miR-20a (1 million-fold), Let-7f (1 million-fold), miR-22–3p (1 million-fold), and Let-7c (364-fold). The top five differentially upregulated miRNAs in eHCC (Table 4) were: miR-101 (215-fold), miR-22 (94-fold), miR-10b (34-fold), miR-19b (34-fold), and miR-192 (29-fold). The top five downregulated were: miR-20a (1 million-fold), miR-22–3p (1 million-fold), miR-26b (1 million-fold), Let-7f (1 million-fold), and miR-30c (3545-fold). The top five differentially upregulated miRNAs in HCC (Table 5) were: miR-142 (1 million-fold), miR-7704 (257-fold), miR-101 (147-fold), miR-23a (124-fold), and miR-22 (85-fold); whereas, the top five downregulated were: miR-122 (513-fold), Let-7g (358-fold), miR-378c (187-fold), miR-185 (68-fold), and miR-451a (58-fold).

**Table 1:** Differentially expressed miRNAs in cirrhosis vs healthy patient’s tissue samples (FDR < 0.05).

microRNA ID	Chromosome	Fold change	FDR
hsa-miR-122-5p	18	−312.73	1.58E-08
hsa-let-7g-5p	3	−204.40	2.12E-04
hsa-mir-103a-2	20	−83.22	3.85E-04
hsa-miR-532-5p	X	−79.93	2.96E-04
hsa-miR-451a	17	−62.96	3.56E-04
hsa-miR-378c	10	−59.31	2.52E-04
hsa-mir-27b	9	−44.44	5.86E-04
hsa-miR-152-3p	17	−44.35	6.26E-04
hsa-mir-194-2	11	−44.16	3.85E-04
hsa-miR-185-5p	22	−39.71	1.44E-03
hsa-miR-128-3p	2	−31.89	2.15E-03
hsa-miR-24-3p	19	−27.07	1.38E-03
hsa-let-7a-5p	11	−25.41	1.22E-04
hsa-let-7b-5p	22	−23.18	6.26E-04
hsa-miR-148b-3p	12	−21.63	9.32E-04
hsa-miR-210-3p	11	−19.22	1.11E-04
hsa-mir-23a	19	−14.26	5.34E-03
hsa-miR-139-5p	11	−14.07	4.82E-03
hsa-mir-101-2	9	−13.98	2.96E-04
hsa-mir-17	13	−13.52	6.00E-03
hsa-miR-99a-5p	21	−12.34	1.77E-04
hsa-miR-93-5p	7	−12.26	6.12E-03
hsa-mir-199a-2	1	−12.07	3.85E-04
hsa-miR-125b-5p	11	−12.00	4.74E-03
hsa-mir-3591	18	−11.52	2.12E-04
hsa-miR-148a-3p	7	−10.94	2.52E-04
hsa-mir-224	X	−10.83	7.73E-03
hsa-mir-23b	9	−10.81	7.29E-03
hsa-mir-3607	5	−9.29	3.71E-04
hsa-miR-450a-5p	X	−7.90	1.22E-02
hsa-miR-200a-3p	1	−7.80	1.08E-02
hsa-mir-140	16	−7.77	1.38E-03
hsa-miR-92a-3p	13	−7.70	1.08E-02
hsa-mir-103a-1	5	−7.57	1.03E-03
hsa-mir-152	17	−7.05	1.49E-02
hsa-mir-378a	5	−6.78	2.78E-02
hsa-miR-126-3p	9	−6.45	1.72E-03
hsa-miR-574-3p	4	−6.41	9.98E-03
hsa-mir-126	9	−6.26	1.55E-02
hsa-mir-128-1	2	−5.62	2.27E-02
hsa-mir-144	17	−5.51	9.16E-03
hsa-miR-29a-3p	7	−5.21	5.08E-03
hsa-mir-21	17	−4.99	3.56E-03
hsa-mir-101-1	1	−4.51	3.95E-03
hsa-let-7e-5p	19	−4.45	2.27E-02
hsa-miR-340-5p	5	−4.04	9.41E-03
hsa-miR-27a-3p	19	−3.92	6.00E-03
hsa-miR-100-5p	11	−3.89	7.36E-03
hsa-miR-26a-5p	12	−3.79	5.34E-03
hsa-mir-199a-1	19	−3.20	8.12E-03
hsa-miR-215-5p	1	−3.07	4.17E-02
hsa-miR-423-3p	17	−3.07	1.60E-02
hsa-miR-21-5p	17	−3.01	1.03E-02
hsa-miR-30b-5p	8	−3.00	2.58E-02
hsa-mir-106b	7	−3.00	1.92E-02
hsa-mir-125b-2	21	−2.96	1.95E-02
hsa-mir-455	9	−2.92	3.76E-02
hsa-mir-195	17	−2.80	3.58E-02
hsa-mir-26b	2	−2.69	1.28E-02
hsa-miR-107	10	−2.62	1.55E-02
hsa-mir-3653	22	−2.58	4.32E-02
hsa-miR-103a-3p	20	−2.40	1.84E-02
hsa-miR-103b	20	−2.40	1.84E-02
hsa-mir-660	X	−2.34	3.88E-02
hsa-let-7a-1	9	−2.21	3.76E-02
hsa-mir-505	X	−2.21	4.32E-02
hsa-mir-192	11	−2.03	2.27E-02
hsa-miR-194-5p	1	−1.86	2.04E-02
hsa-miR-25-3p	7	−1.84	3.23E-02
hsa-let-7i	12	−1.65	4.73E-02
hsa-let-7i-5p	12	−1.58	4.77E-02
hsa-mir-148a	7	−1.48	2.51E-02
hsa-mir-409	14	7.84	4.00E-02
hsa-miR-769-5p	19	8.13	6.63E-03
hsa-miR-320b	1	14.14	2.89E-04
hsa-mir-136	14	15.82	1.80E-02
hsa-miR-127-3p	14	17.15	3.85E-04
hsa-miR-320a	8	17.51	9.02E-06
hsa-mir-186	1	19.52	3.85E-04
hsa-mir-181c	19	24.73	4.46E-02
hsa-miR-1307-5p	10	26.74	6.00E-03
hsa-mir-10b	2	28.57	3.03E-02
hsa-miR-192-5p	11	31.98	1.83E-02
hsa-mir-486-2	8	75.42	9.02E-06
hsa-miR-101-3p	1	113.21	2.51E-02
hsa-mir-22	17	142.96	1.05E-07
hsa-miR-7704	2	403.15	1.11E-04

**Table 2:** Differentially expressed miRNAs in low-grade dysplastic nodule vs healthy patient’s tissue samples (FDR < 0.05).

microRNA ID	Chromosome	Fold change	FDR
hsa-miR-141-3p	12	625.50	7.60E-04
hsa-miR-101-3p	1	208.06	7.45E-06
hsa-mir-22	17	111.76	6.50E-10
hsa-mir-16-1	13	60.69	1.53E-05
hsa-mir-486-2	8	34.93	7.48E-06
hsa-miR-192-5p	11	34.41	2.10E-06
hsa-mir-150	19	33.64	2.15E-04
hsa-miR-199a-3p	1	32.48	8.85E-04
hsa-mir-19b-2	X	32.27	4.61E-06
hsa-miR-15a-5p	13	26.71	1.98E-03
hsa-mir-10b	2	26.66	7.48E-06
hsa-mir-181c	19	26.55	7.45E-06
hsa-mir-181a-1	1	23.72	9.13E-06
hsa-mir-130a	11	22.38	4.29E-05
hsa-miR-424-5p	X	21.67	1.24E-04
hsa-let-7f-1	9	20.45	8.44E-05
hsa-mir-186	1	15.49	7.48E-06
hsa-miR-30a-5p	6	15.34	1.37E-03
hsa-miR-23b-3p	9	13.80	7.60E-04
hsa-mir-26a-1	3	11.20	1.99E-03
hsa-miR-1307-5p	10	10.97	4.06E-02
hsa-mir-181b-1	1	10.53	5.98E-05
hsa-mir-92a-1	13	9.89	1.51E-04
hsa-miR-182-5p	7	9.56	2.65E-02
hsa-mir-10a	17	9.26	2.38E-04
hsa-mir-26a-2	12	8.35	8.27E-03
hsa-mir-191	3	8.25	2.67E-03
hsa-mir-92a-2	X	7.75	1.55E-03
hsa-mir-340	5	7.19	2.26E-03
hsa-miR-126-5p	9	6.67	6.16E-03
hsa-miR-29c-3p	1	6.49	9.39E-04
hsa-mir-16-2	3	6.39	3.99E-02
hsa-miR-127-3p	14	6.04	3.77E-03
hsa-mir-19a	13	5.91	2.26E-03
hsa-mir-30b	8	5.51	1.27E-02
hsa-mir-155	21	5.47	1.70E-02
hsa-mir-100	11	5.34	5.61E-04
hsa-miR-10b-5p	2	5.12	4.41E-03
hsa-mir-145	5	4.71	8.60E-03
hsa-let-7d	9	4.66	2.23E-02
hsa-miR-320a	8	4.27	2.86E-03
hsa-mir-181b-2	9	3.70	1.65E-02
hsa-mir-532	X	3.63	4.22E-02
hsa-mir-125b-1	11	3.18	1.70E-02
hsa-mir-34a	1	2.94	4.06E-02
hsa-mir-193b	16	2.45	4.20E-02
hsa-miR-200a-3p	1	−1.78	4.37E-02
hsa-let-7i-5p	12	−1.78	4.66E-02
hsa-miR-215-5p	1	−1.96	3.38E-02
hsa-mir-455	9	−2.03	4.12E-02
hsa-mir-21	17	−2.08	2.65E-02
hsa-let-7e-5p	19	−2.10	3.33E-02
hsa-mir-3120	1	−2.20	3.66E-02
hsa-miR-92a-3p	13	−2.21	1.74E-02
hsa-mir-101-1	1	−2.22	3.72E-02
hsa-mir-99a	21	−2.28	1.74E-02
hsa-mir-103a-1	5	−2.29	2.11E-02
hsa-mir-224	X	−2.34	3.22E-02
hsa-mir-106b	7	−2.34	1.61E-02
hsa-mir-140	16	−3.01	6.10E-03
hsa-mir-320a	8	−3.02	3.79E-03
hsa-mir-23b	9	−3.12	7.86E-03
hsa-mir-144	17	−3.19	4.12E-02
hsa-mir-152	17	−3.28	2.51E-03
hsa-mir-125b-2	21	−3.47	4.41E-03
hsa-miR-340-5p	5	−3.52	2.16E-03
hsa-miR-148a-3p	7	−4.16	3.07E-03
hsa-miR-148b-3p	12	−4.20	6.24E-03
hsa-mir-17	13	−4.21	1.70E-03
hsa-miR-423-3p	17	−4.43	4.41E-03
hsa-mir-3607	5	−4.44	1.60E-03
hsa-miR-93-5p	7	−4.74	7.05E-04
hsa-mir-126	9	−4.75	5.55E-04
hsa-mir-3653	22	−4.81	1.33E-04
hsa-miR-126-3p	9	−4.89	2.43E-04
hsa-mir-23a	19	−5.30	1.44E-03
hsa-miR-24-3p	19	−5.44	2.26E-03
hsa-let-7a-5p	11	−5.53	7.60E-04
hsa-miR-139-5p	11	−5.53	3.98E-04
hsa-mir-3591	18	−6.30	2.41E-04
hsa-mir-199a-2	1	−6.72	1.77E-04
hsa-let-7b-5p	22	−6.81	8.14E-04
hsa-mir-101-2	9	−6.99	2.88E-04
hsa-mir-378a	5	−7.06	5.82E-05
hsa-miR-100-5p	11	−7.71	1.06E-05
hsa-miR-128-3p	2	−8.01	5.61E-04
hsa-mir-194-2	11	−8.52	3.03E-04
hsa-let-7d-5p	9	−10.76	9.51E-03
hsa-miR-450a-5p	X	−12.31	2.38E-05
hsa-miR-210-3p	11	−12.69	1.77E-05
hsa-miR-152-3p	17	−14.35	2.06E-05
hsa-miR-532-5p	X	−15.17	3.77E-03
hsa-mir-424	X	−15.74	1.47E-05
hsa-mir-27b	9	−17.77	7.48E-06
hsa-let-7c-5p	21	−19.23	1.62E-03
hsa-miR-125b-5p	11	−20.94	1.17E-05
hsa-miR-99a-5p	21	−27.01	4.07E-08
hsa-mir-103a-2	20	−28.04	4.82E-06
hsa-miR-185-5p	22	−68.06	2.60E-08
hsa-miR-374a-5p	X	−69.55	4.61E-06
hsa-let-7g-5p	3	−77.05	4.07E-08
hsa-miR-30c-5p	1	−77.77	1.33E-04
hsa-miR-122-5p	18	−80.83	4.82E-06
hsa-miR-361-3p	X	−94.56	3.30E-05
hsa-miR-378c	10	−103.67	5.71E-05
hsa-miR-451a	17	−513.04	2.05E-06

**Table 3:** Differentially expressed miRNAs in high-grade dysplastic nodule vs healthy patient’s tissue samples (FDR < 0.05).

microRNA ID	Chromosome	Fold change	FDR
hsa-miR-101-3p	1	266.25	1.10E-02
hsa-mir-22	17	169.91	1.30E-10
hsa-mir-16-1	13	53.70	5.87E-03
hsa-miR-192-5p	11	45.02	1.43E-04
hsa-mir-19b-2	X	34.12	1.68E-03
hsa-mir-486-2	8	25.83	1.76E-04
hsa-mir-130a	11	24.80	4.06E-02
hsa-mir-186	1	23.55	2.97E-06
hsa-miR-424-5p	X	20.63	1.50E-02
hsa-mir-10b	2	16.15	1.11E-02
hsa-mir-181a-1	1	16.09	4.85E-04
hsa-miR-320a	8	13.48	2.01E-02
hsa-mir-10a	17	8.93	2.30E-02
hsa-mir-191	3	8.80	2.81E-02
hsa-mir-92a-1	13	8.19	1.46E-02
hsa-miR-127-3p	14	4.94	8.02E-03
hsa-mir-193b	16	4.90	1.09E-02
hsa-mir-100	11	4.11	3.72E-03
hsa-mir-125b-2	21	−2.90	4.18E-02
hsa-mir-106b	7	−3.01	4.27E-02
hsa-miR-99b-5p	19	−3.02	2.47E-02
hsa-mir-200b	1	−3.81	2.62E-02
hsa-mir-140	16	−3.91	1.53E-02
hsa-miR-148b-3p	12	−3.99	2.34E-02
hsa-miR-126-3p	9	−4.34	1.67E-02
hsa-mir-3591	18	−4.43	1.09E-02
hsa-miR-100-5p	11	−4.74	1.48E-03
hsa-mir-3607	5	−5.39	1.35E-02
hsa-miR-423-3p	17	−6.19	3.76E-03
hsa-mir-101-2	9	−6.49	1.53E-02
hsa-miR-93-5p	7	−6.63	2.12E-03
hsa-let-7a-5p	11	−6.99	1.31E-02
hsa-miR-24-3p	19	−7.33	5.92E-03
hsa-mir-199a-2	1	−7.90	1.40E-03
hsa-miR-450a-5p	X	−10.52	3.21E-02
hsa-miR-374a-5p	X	−13.20	1.31E-02
hsa-miR-99a-5p	21	−13.46	2.70E-05
hsa-mir-27b	9	−14.75	4.93E-04
hsa-miR-152-3p	17	−14.77	1.82E-02
hsa-miR-125b-5p	11	−15.62	6.66E-03
hsa-miR-128-3p	2	−16.39	2.13E-02
hsa-miR-210-3p	11	−16.54	1.43E-04
hsa-mir-424	X	−21.44	1.78E-02
hsa-let-7b-5p	22	−21.90	1.81E-02
hsa-miR-200a-3p	1	−24.56	1.34E-02
hsa-miR-532-5p	X	−27.50	1.64E-04
hsa-mir-103a-2	20	−30.41	1.35E-02
hsa-miR-451a	17	−35.59	1.48E-03
hsa-let-7d-5p	9	−35.82	5.12E-03
hsa-miR-185-5p	22	−70.25	3.66E-07
hsa-miR-378c	10	−76.83	2.87E-05
hsa-let-7g-5p	3	−237.19	1.48E-03
hsa-miR-122-5p	18	−243.58	4.71E-07
hsa-miR-30c-5p	1	−298.01	1.10E-04
hsa-let-7c-5p	21	−364.58	3.61E-05
hsa-miR-22-3p	17	−1000000.00	1.03E-16
hsa-let-7f-5p	9	−1000000.00	1.03E-16
hsa-miR-26b-5p	2	−1000000.00	1.95E-16
hsa-miR-20a-5p	13	−1000000.00	3.67E-16

**Table 4:** Differentially expressed miRNAs in early hepatocellular carcinoma vs healthy patient’s tissue samples (FDR < 0.05).

microRNA ID	Chromosome	Fold change	FDR
hsa-miR-101-3p	1	215.60	4.49E-03
hsa-mir-22	17	94.20	9.45E-09
hsa-mir-10b	2	34.24	9.28E-04
hsa-mir-19b-2	X	29.45	9.73E-03
hsa-miR-192-5p	11	29.09	1.14E-03
hsa-mir-486-2	8	26.76	7.29E-04
hsa-miR-320a	8	18.04	9.73E-03
hsa-mir-186	1	16.42	6.95E-05
hsa-miR-127-3p	14	15.94	1.09E-02
hsa-mir-181a-1	1	9.96	2.97E-03
hsa-mir-193b	16	3.63	1.84E-02
hsa-mir-100	11	3.29	1.06E-02
hsa-mir-21	17	−2.07	3.11E-02
hsa-miR-92a-3p	13	−2.20	1.53E-02
hsa-let-7i-5p	12	−2.39	1.84E-02
hsa-miR-99b-5p	19	−2.48	1.84E-02
hsa-mir-455	9	−2.60	2.48E-02
hsa-mir-99a	21	−2.70	1.60E-02
hsa-mir-200b	1	−2.76	2.49E-02
hsa-mir-3653	22	−2.77	1.72E-02
hsa-let-7i	12	−2.78	1.84E-02
hsa-mir-200a	1	−2.80	3.77E-02
hsa-miR-29a-3p	7	−2.87	1.84E-02
hsa-miR-215-5p	1	−2.89	1.77E-02
hsa-mir-101-1	1	−2.97	1.63E-02
hsa-let-7e-5p	19	−3.05	1.59E-02
hsa-miR-27a-3p	19	−3.47	1.44E-02
hsa-mir-106b	7	−3.60	7.83E-03
hsa-mir-128-1	2	−3.63	2.07E-02
hsa-miR-340-5p	5	−3.87	8.94E-03
hsa-miR-150-5p	19	−3.90	1.06E-02
hsa-mir-199a-1	19	−4.02	9.64E-03
hsa-miR-148b-3p	12	−4.14	5.46E-03
hsa-mir-23b	9	−4.38	9.73E-03
hsa-miR-148a-3p	7	−4.55	8.26E-03
hsa-mir-125b-2	21	−4.84	5.35E-03
hsa-mir-103a-1	5	−5.16	5.75E-03
hsa-mir-17	13	−5.22	6.28E-03
hsa-mir-152	17	−5.92	2.77E-03
hsa-mir-3607	5	−6.14	3.00E-03
hsa-miR-100-5p	11	−6.19	4.14E-04
hsa-mir-126	9	−7.00	2.19E-03
hsa-let-7a-5p	11	−7.06	2.77E-03
hsa-mir-140	16	−7.07	2.31E-03
hsa-miR-126-3p	9	−7.18	1.73E-03
hsa-mir-23a	19	−8.07	1.47E-03
hsa-miR-93-5p	7	−8.58	8.01E-04
hsa-mir-3591	18	−8.87	7.27E-04
hsa-mir-101-2	9	−9.79	2.08E-03
hsa-miR-450a-5p	X	−10.96	7.29E-04
hsa-miR-24-3p	19	−11.91	2.08E-03
hsa-mir-144	17	−12.34	4.51E-03
hsa-mir-194-2	11	−12.62	1.84E-03
hsa-miR-200a-3p	1	−12.71	1.39E-02
hsa-miR-139-5p	11	−13.55	1.18E-02
hsa-miR-210-3p	11	−13.69	8.98E-05
hsa-mir-199a-2	1	−14.20	3.57E-04
hsa-miR-128-3p	2	−18.16	7.74E-03
hsa-miR-152-3p	17	−18.61	3.51E-03
hsa-miR-374a-5p	X	−19.82	1.14E-03
hsa-miR-99a-5p	21	−20.45	5.10E-06
hsa-let-7b-5p	22	−21.06	9.64E-03
hsa-miR-125b-5p	11	−24.27	1.78E-04
hsa-miR-532-5p	X	−27.40	5.35E-03
hsa-let-7d-5p	9	−32.21	3.51E-03
hsa-mir-27b	9	−32.67	1.05E-04
hsa-mir-424	X	−46.39	3.61E-03
hsa-mir-103a-2	20	−51.50	4.95E-06
hsa-miR-185-5p	22	−58.42	4.53E-06
hsa-let-7c-5p	21	−68.82	2.19E-03
hsa-miR-378c	10	−82.90	6.70E-07
hsa-miR-451a	17	−188.05	4.57E-04
hsa-miR-122-5p	18	−236.49	5.10E-06
hsa-let-7g-5p	3	−405.25	9.45E-09
hsa-miR-361-3p	X	−2902.32	6.70E-07
hsa-miR-30c-5p	1	−3544.83	2.77E-06
hsa-let-7f-5p	9	−1000000.00	5.42E-17
hsa-miR-22-3p	17	−1000000.00	5.42E-17
hsa-miR-26b-5p	2	−1000000.00	3.58E-16
hsa-miR-20a-5p	13	−1000000.00	4.76E-16

**Table 5:** Differentially expressed miRNAs in hepatocellular carcinoma vs healthy patient’s tissue samples (FDR < 0.05).

microRNA ID	Chromosome	Fold change	FDR
hsa-miR-142-5p	17	1000000.00	2.06E-11
hsa-miR-7704	2	256.97	8.73E-05
hsa-miR-101-3p	1	147.24	6.28E-08
hsa-miR-23a-3p	19	123.92	3.72E-08
hsa-mir-22	17	85.32	5.45E-17
hsa-mir-10b	2	73.25	2.02E-10
hsa-miR-130b-3p	22	64.54	1.41E-06
hsa-mir-182	7	61.30	1.51E-02
hsa-mir-16-1	13	57.49	2.69E-07
hsa-miR-197-3p	1	40.60	7.72E-07
hsa-miR-192-5p	11	30.83	1.59E-10
hsa-miR-15a-5p	13	28.71	3.39E-05
hsa-mir-19b-2	X	28.71	6.50E-06
hsa-mir-486-2	8	26.10	2.41E-08
hsa-mir-150	19	23.61	2.20E-03
hsa-mir-98	X	22.41	2.99E-02
hsa-miR-199a-3p	1	21.84	3.26E-04
hsa-mir-181c	19	20.83	2.27E-06
hsa-mir-183	7	19.92	1.02E-03
hsa-mir-181a-1	1	17.61	3.21E-05
hsa-miR-182-5p	7	17.27	1.32E-03
hsa-miR-23b-3p	9	15.46	8.41E-06
hsa-mir-136	14	15.45	8.72E-06
hsa-mir-186	1	15.42	3.23E-10
hsa-mir-130a	11	14.61	1.33E-03
hsa-miR-1307-5p	10	14.53	4.71E-03
hsa-miR-10b-5p	2	13.00	7.50E-07
hsa-let-7f-1	9	12.70	5.27E-05
hsa-mir-92a-1	13	10.86	3.79E-04
hsa-mir-193a	17	10.85	3.49E-05
hsa-miR-126-5p	9	10.17	9.18E-03
hsa-mir-191	3	10.03	1.28E-04
hsa-mir-26a-1	3	8.35	3.16E-03
hsa-mir-181b-1	1	8.16	3.33E-04
hsa-miR-30a-5p	6	8.04	3.05E-04
hsa-mir-30b	8	7.93	4.25E-03
hsa-mir-10a	17	7.78	1.28E-03
hsa-mir-29b-2	1	7.10	1.55E-02
hsa-miR-204-5p	9	6.73	1.89E-03
hsa-mir-93	7	6.48	1.29E-03
hsa-mir-26a-2	12	6.36	1.10E-02
hsa-mir-92a-2	X	6.11	2.72E-03
hsa-miR-127-3p	14	6.02	6.97E-05
hsa-mir-16-2	3	6.00	9.69E-03
hsa-mir-19a	13	5.72	4.58E-03
hsa-miR-29c-3p	1	5.45	1.02E-03
hsa-mir-340	5	5.24	4.02E-03
hsa-mir-145	5	5.22	2.99E-02
hsa-mir-532	X	5.15	1.77E-03
hsa-mir-15b	3	5.13	3.17E-02
hsa-mir-155	21	5.10	4.11E-03
hsa-mir-193b	16	4.87	1.51E-05
hsa-miR-320a	8	4.43	2.20E-03
hsa-miR-484	16	4.20	1.95E-02
hsa-mir-24-1	9	4.11	3.43E-02
hsa-mir-29c	1	3.99	3.36E-02
hsa-mir-365b	17	3.90	1.94E-02
hsa-miR-27b-3p	9	3.76	3.10E-02
hsa-mir-34a	1	3.22	1.32E-03
hsa-miR-769-5p	19	2.90	3.59E-02
hsa-mir-100	11	2.79	4.71E-03
hsa-mir-125b-1	11	2.68	1.72E-02
hsa-miR-221-3p	X	2.68	1.72E-02
hsa-mir-409	14	2.33	1.94E-02
hsa-miR-146a-5p	5	−1.92	1.72E-02
hsa-let-7i	12	−1.95	3.26E-02
hsa-let-7i-5p	12	−1.99	1.29E-02
hsa-miR-486-5p	8	−2.02	3.41E-02
hsa-mir-106b	7	−2.04	1.77E-02
hsa-miR-378a-3p	5	−2.17	1.64E-03
hsa-let-7e-5p	19	−2.21	2.28E-02
hsa-miR-429	1	−2.26	1.60E-02
hsa-mir-484	16	−2.27	4.71E-02
hsa-mir-103a-1	5	−2.36	1.72E-02
hsa-mir-140	16	−2.41	6.53E-03
hsa-miR-92a-3p	13	−2.43	2.22E-02
hsa-mir-455	9	−2.46	2.18E-02
hsa-miR-29a-3p	7	−2.50	2.40E-02
hsa-mir-320a	8	−2.69	1.73E-03
hsa-miR-375	2	−2.75	1.34E-02
hsa-mir-505	X	−2.93	1.88E-03
hsa-mir-99a	21	−3.18	1.99E-04
hsa-miR-215-5p	1	−3.25	4.40E-04
hsa-mir-23b	9	−3.28	1.83E-02
hsa-miR-340-5p	5	−3.48	1.93E-03
hsa-miR-148b-3p	12	−3.71	4.08E-03
hsa-mir-152	17	−3.71	6.20E-04
hsa-mir-126	9	−3.78	3.83E-03
hsa-miR-93-5p	7	−3.80	5.19E-04
hsa-mir-125b-2	21	−3.81	4.58E-04
hsa-mir-17	13	−3.86	2.18E-03
hsa-miR-423-3p	17	−3.90	2.13E-04
hsa-mir-23a	19	−4.19	4.31E-02
hsa-mir-101-1	1	−4.25	8.10E-04
hsa-miR-126-3p	9	−4.32	3.55E-04
hsa-mir-3607	5	−4.41	5.43E-04
hsa-mir-3653	22	−4.48	4.14E-06
hsa-miR-29b-3p	1	−4.55	3.10E-02
hsa-mir-185	22	−4.72	8.54E-05
hsa-mir-200b	1	−4.78	1.02E-03
hsa-mir-200a	1	−4.85	3.16E-03
hsa-miR-148a-3p	7	−5.38	3.59E-05
hsa-miR-24-3p	19	−5.67	1.42E-03
hsa-mir-144	17	−5.79	2.76E-03
hsa-mir-223	X	−6.03	1.37E-02
hsa-mir-3591	18	−7.35	1.58E-06
hsa-miR-139-5p	11	−8.35	5.27E-05
hsa-mir-199a-2	1	−8.74	9.79E-03
hsa-miR-100-5p	11	−8.93	3.26E-09
hsa-let-7a-5p	11	−9.03	5.31E-05
hsa-miR-128-3p	2	−9.93	3.52E-05
hsa-miR-365b-3p	17	−10.55	3.70E-03
hsa-mir-378a	5	−11.05	3.72E-08
hsa-miR-374a-5p	X	−12.17	1.28E-03
hsa-mir-194-2	11	−13.00	9.17E-06
hsa-mir-101-2	9	−13.03	5.26E-07
hsa-miR-532-5p	X	−14.56	9.31E-04
hsa-miR-450a-5p	X	−14.85	1.26E-06
hsa-let-7b-5p	22	−15.34	1.49E-04
hsa-miR-365a-3p	16	−16.01	1.16E-03
hsa-miR-152-3p	17	−16.10	5.27E-05
hsa-miR-200a-3p	1	−21.02	5.19E-04
hsa-mir-27b	9	−22.30	9.04E-08
hsa-mir-424	X	−23.09	1.42E-03
hsa-mir-103a-2	20	−23.41	1.78E-03
hsa-miR-210-3p	11	−27.21	3.26E-09
hsa-miR-99a-5p	21	−29.63	4.87E-12
hsa-miR-125b-5p	11	−32.70	3.37E-09
hsa-miR-451a	17	−57.80	2.07E-04
hsa-miR-185-5p	22	−67.83	4.08E-11
hsa-miR-378c	10	−187.61	4.37E-07
hsa-let-7g-5p	3	−358.28	1.34E-09
hsa-miR-122-5p	18	−513.22	1.17E-06

Broadly visualized data for all five groups was represented in a Venn diagram (Fig. 1F), which showed 37 miRs are commonly expressed in all groups whereas, 16 miRs in cirrhosis, four miRs in LGDN, none in HGDN, one miR in eHCC, and 29 miRs in HCC are uniquely expressed (Fig. 1F). Several miRs expression patterns were common between groups, specifically 24 miRs in LGDN and HCC and 18 miRs in cirrhosis, LGDN, eHCC, and HCC (Fig. 1F). Circos Plots were prepared for comprehensive visualization of differentially expressed miRs in all five groups, including chromosome number and location (Fig. 1G). Black lines inside respective rings show the affected gene location and chromosome number. After plugging the data into Circos Plot, we found chromosomes 1, 2, 9, 13, and 17 were the most enriched chromosomes in all five groups (Fig. 1G).

### Validation of Differentially Expressed miRNAs in HCC Clinical Samples

In order to validate in silico miRNA sequencing data from a publically available source, we used human HCC specimens compared to healthy liver tissues by using an absolute quantification miRNA assay from NanoString technologies, which screens for more than 800 human miRNAs, validated to an independent cohort of tissue samples. In our analysis, we found more than 274 miRNAs were differentially expressed by more than a two fold change compared to healthy liver samples. We then exported the data using fold change (up and down regulated miRNAs) and compared miRNA-seq differentially expressed miRNAs in the HCC group using FunRich software. We created a grouped heatmap for the top 20 upregulated and downregulated miRNAs (Fig. 1I) and compared differentially expressed genes between the miRNA-seq and NanoString miRNA assays (Fig. 1H). We identified 21 miRNAs using a Venn diagram with 11 of them showing similar trends as the sequenced data (Table 6). Five miRNAs were upregulated (miR-130b, miR-182, miR10b, miR320a, and miR769) ranging from a 2.9 to 30-fold induction in the NanoString miRNA assay, whereas six miRNAs were downregulated (miR122, miR451a, miR200a, miR139, miR148a, and miR375) ranging from −2.2 to −6 fold (Table 6).

**Table 6:** Validation of Differentially Expressed miRNAs in HCC miRNA sequence data and HCC live tissue Samples.

HCC miRNA-seq		HCC Nanostring data-2FC	
microRNA ID	Fold change	miRNA	Fold Change
hsa-miR-130b-3p	64.54	hsa-miR-130b-3p	30.06
hsa-miR-182-5p	17.27	hsa-miR-182-5p	3.97
hsa-miR-10b-5p	13.00	hsa-miR-10b-5p	4.08
hsa-miR-320a	4.43	hsa-miR-320a	8.68
hsa-miR-769-5p	2.90	hsa-miR-769-5p	2.91
hsa-miR-375	−2.75	hsa-miR-375	−4.54
hsa-miR-148a-3p	−5.38	hsa-miR-148a-3p	−2.28
hsa-miR-139-5p	−8.35	hsa-miR-139-5p	−6.01
hsa-miR-200a-3p	−21.02	hsa-miR-200a-3p	−5.43
hsa-miR-451a	−57.80	hsa-miR-451a	−3.49
hsa-miR-122-5p	−513.22	hsa-miR-122-5p	−3.31

**Figure 2:**
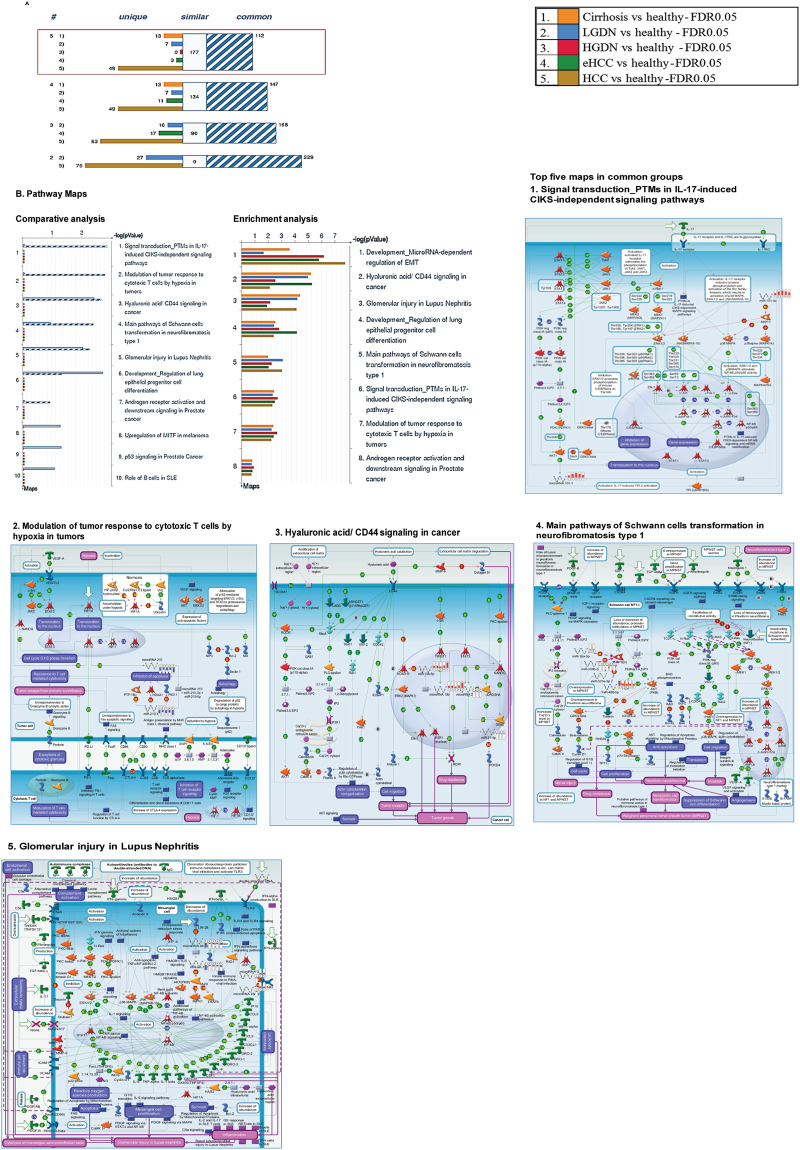
Enrichment analysis of microRNA for Pathway Maps, Gene Ontology, Disease by Biomarker and Network processes in liver diseases. Pathway analysis was carried out via MetaCore software, differentially expressed miRNA data for Cirrhosis, Low-grade dysplastic nodule, High-grade dysplastic nodule, Early stage Hepatocellular carcinoma, and Advanced stage Hepatocellular carcinoma were uploaded to MetaCore server and the most significantly affected pathways were created using comparative enrichment analysis. The gene content were aligned between all listed experiments above. The intersection set of experiments is defined as “common” and marked as a blue/white striped bar. The unique genes for the experiments are marked as colored bars. The genes from the “similar” set are present in all but one (any) file. The parameters for comparison are set as above. Enrichment analysis consists of matching gene IDs of possible targets for the “common”, “similar” and “unique” sets with gene IDs in functional ontologies in MetaCore. The probability of a random intersection between set IDs the size of the target list with ontology entities estimated in *p*-value of hypergeometric intersection. The lower *p*-value means higher relevance of the entity to the dataset, which shows in a higher rating for the entity (**A**) there is a unique signature in advanced hepatocellular carcinoma. (**B**) Pathway Maps: Comparative and enrichment pathway analysis showed most of the miRNAs enriched in various disease stages were involved the oncogenic pathways (The results were obtained using MetaCore pathways analysis tool; GeneGo/Thomson Reuters). Top five common pathways were listed (B1–B5). Experimental data was visualized on the maps as blue (for downregulation) and red (upregulation) histograms. The height of the histogram corresponds to the relative expression value for a particular gene/protein (Pathway maps were obtained from MetaCore pathways analysis tool; GeneGo/Thomson Reuters).

**Figure 3:**
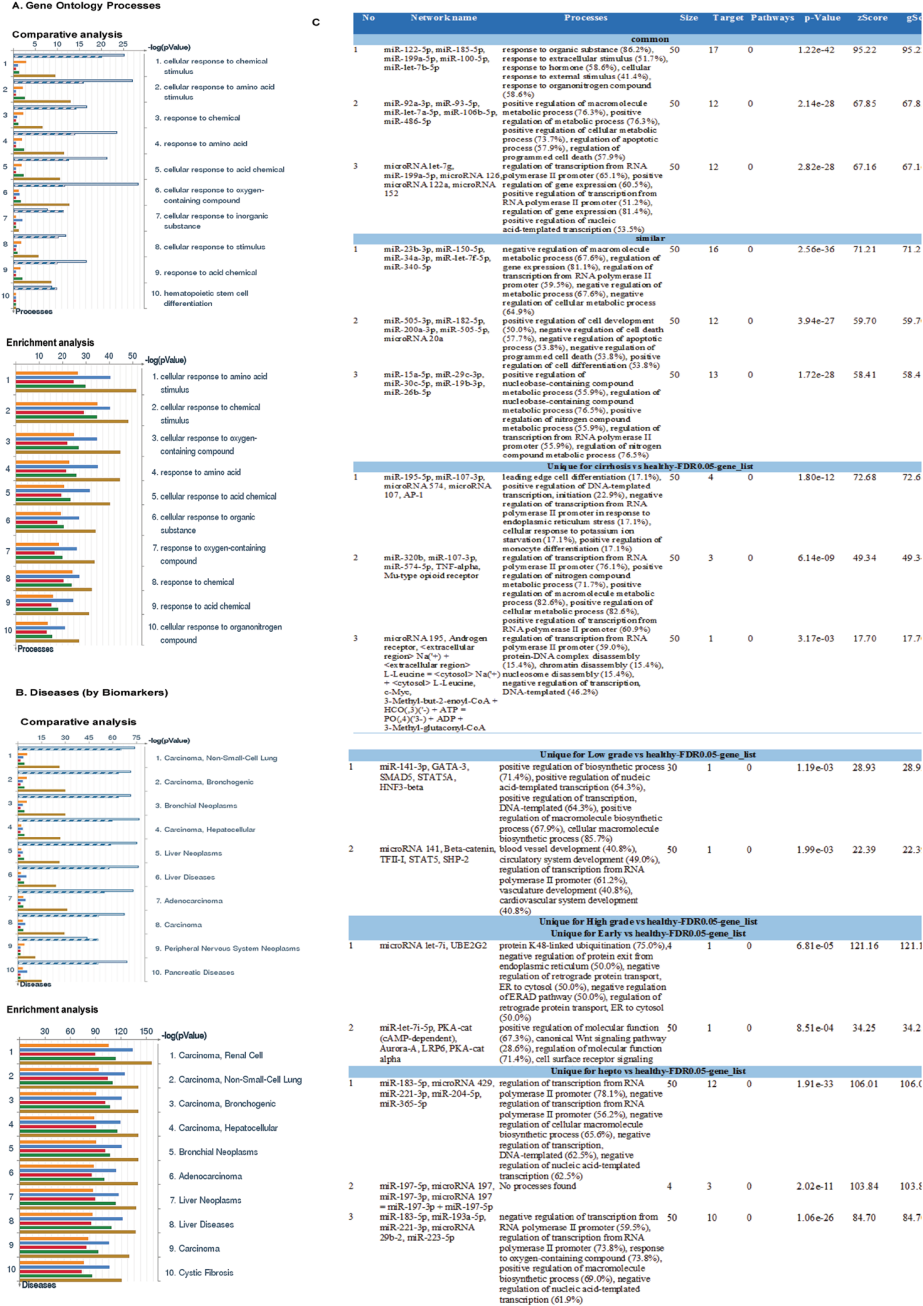
Enrichment analysis of microRNA for Gene Ontology in liver diseases. (**A**) GO Biological Processes: Comparative and enrichment analysis of GO processes shown in various disease stages. (**B**) Disease status: Comparative and enrichment analysis of disease by biomarkers shown in various disease stages, significantly affected miRNAs in liver disease included various carcinoma pathways. Disease folders were organized into a hierarchical tree. Gene content may vary greatly between such complex diseases as cancers and some Mendelian diseases. In addition, coverage of different diseases in the literature were skewed. These two factors may affect p-value prioritization for diseases. (**C**) Biological network analysis: Differentially expressed miRNA data were analyzed for the biological networks involved in liver disease, we presented a list of top score networks in common, similar, and unique groups.

**Figure 4:**
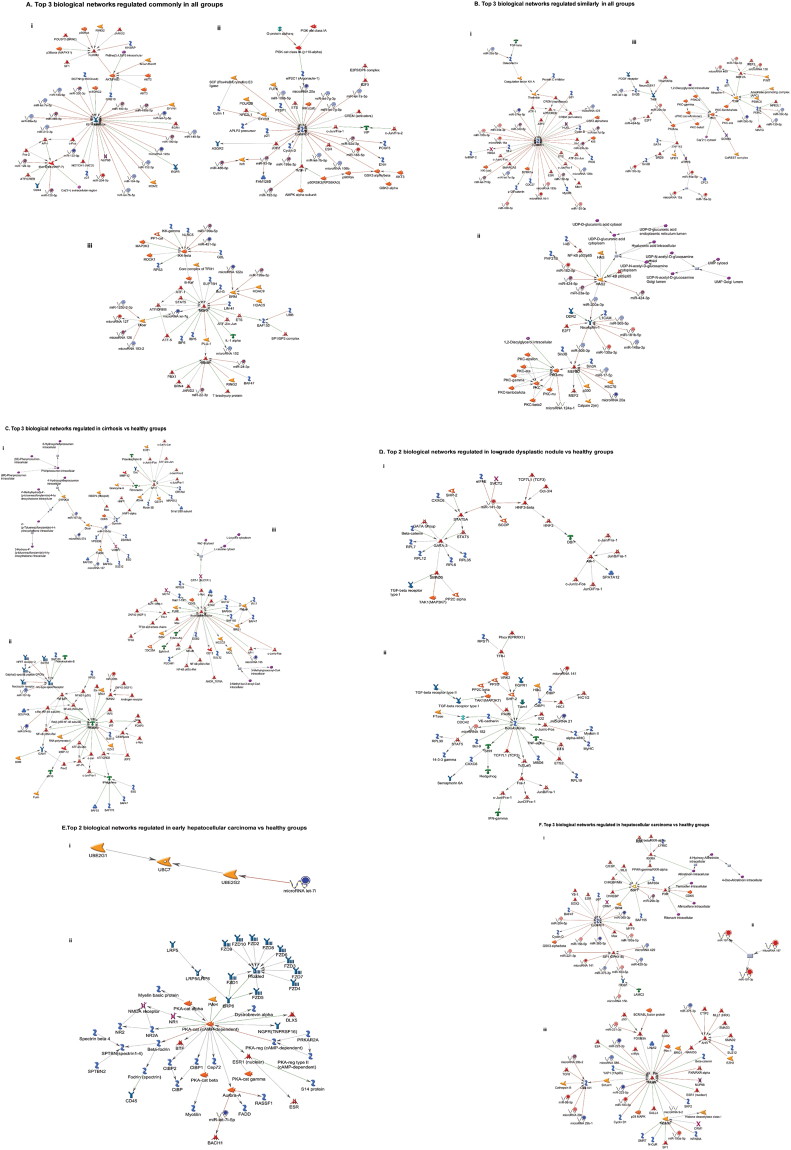
Enrichment analysis of microRNA for Network processes in liver diseases. (**A**–**F**) Top biological networks: The top three networks that are involved in Cirrhosis, Low-grade dysplastic nodule, High-grade dysplastic nodule, Early stage Hepatocellular carcinoma and Advanced stage Hepatocellular carcinoma with common, similar, and unique groups. This is a variant of the shortest paths algorithm with main parameters of enrichment. Enriched miRNAs were prioritized based on the number of fragments of canonical pathways on the networks. Up-regulated genes were marked with red circles and down-regulated genes with blue circles. The ‘checkerboard’ color indicates mixed expression for the gene between files or multiple tags for the same gene.

Following miRNA expression analysis, we used MetaCore pathway software to analyze the possible signaling pathways affected and enriched microRNAs role in cirrhosis and HCC pathogenesis. We uploaded all the differentially expressed miRNAs to the MetaCore server for comparative and gene enrichment analysis (Figs 2, 3 and 4). Processed data for comparative enrichment analysis showed a three way representation (common-high significance in all groups, similar-similarly enriched in all groups, and unique-various pathways specifically expressed in the individual groups). We found 112 pathways to be common, 177 similar, and a number unique to each group. 49 pathways were unique to advanced HCC data sets (Fig. 2A). Pathway Maps were utilized for comparative and enrichment analysis of differentially expressed miRNAs for biological pathways analysis (The results were obtained using MetaCore pathways analysis tool; GeneGo/Thomson Reuters). Diseased samples clearly showed involvement of signaling molecules in microRNA dependent EMT, CD44 signaling, and others (Fig. 2B). Gene Ontology (GO) Processes analysis revealed that most affected miRNAs were involved in the cellular response to amino acid and chemical stimulus, with HCC being the most affected group (Fig. 3A). Many cancer-related categories were over-represented via Disease Stages by Biomarkers analysis (Fig. 3B). Disease Stages by Biomarkers analysis found the most affected miRNAs were involved in cancer signaling pathways including renal, non-small cell lung, bronchogenic, and hepatocellular cancers (Fig. 3A). Biological network analysis of miRNAs involved in the highest affected network processes are listed in Fig. 4A with the top three commonly regulated networks in all five groups (Fig. 4A), top three similarly affected networks (Fig. 4B), and top uniquely regulated biological network pathways in liver disease shown in Fig. 4C–F.

### Differential Expression of piRNA in Cirrhosis, LGDN, HGDN, eHCC, and HCC Tissue Samples

**Figure 5:**
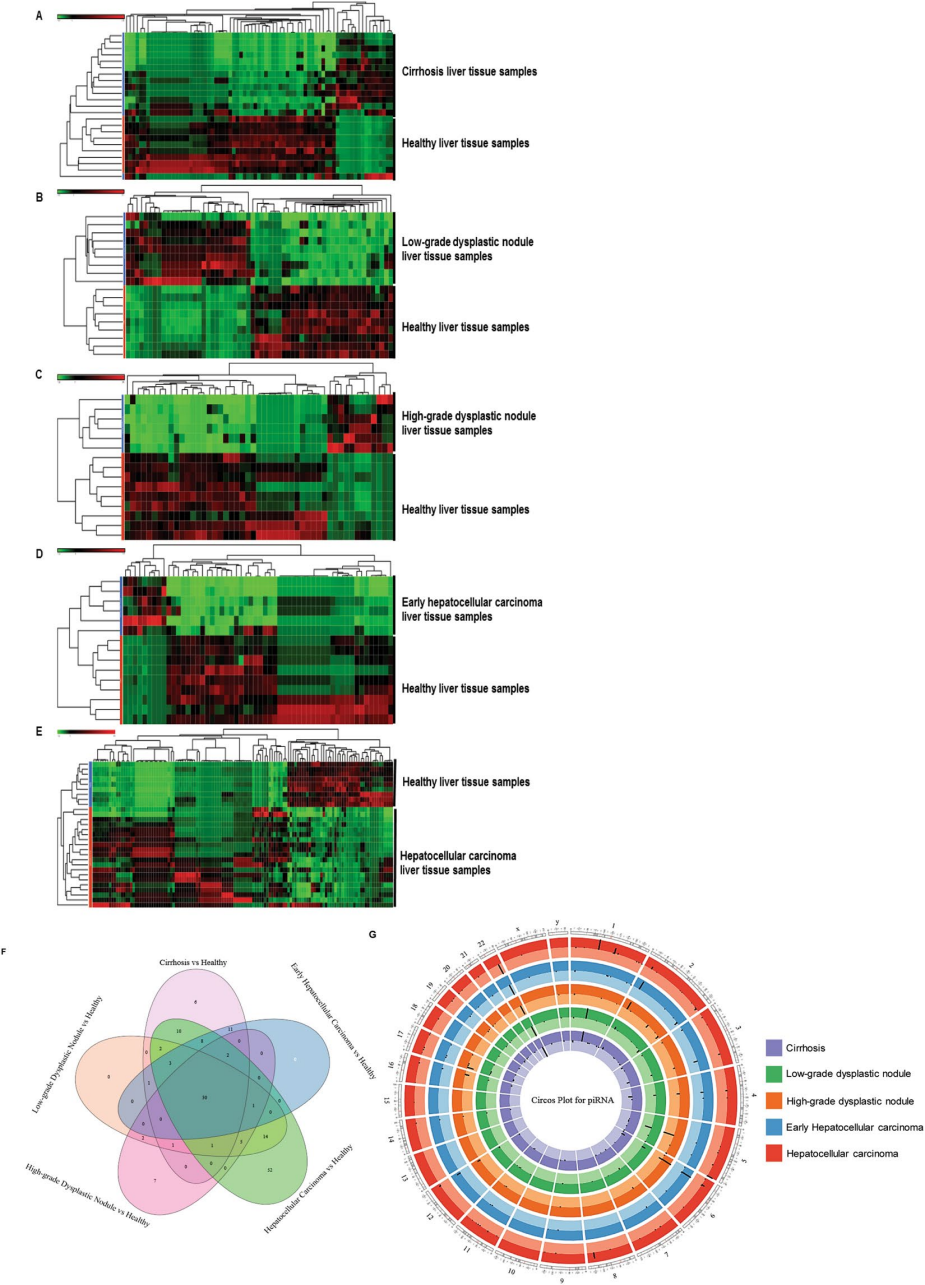
Differential expression of piRNAs in liver tissue samples: Differentially expressed piRNAs were quantified and a heatmap was prepared (*FDR* < *0.05*) for each disease stage (Cirrhosis, Low-grade dysplastic nodule, High-grade dysplastic nodule, Early stage Hepatocellular carcinoma and Advanced stage Hepatocellular carcinoma) with healthy control samples (**A**–**E**). All enriched piRNAs were summarized by a Venn diagram, which identified 30 piRNAs commonly expressed in all stages and 52 piRs were specifically dysregulated in advanced hepatocellular carcinoma (**F**). (**G**) A Circos plot was prepared incorporating all differential expressions of piRNAs in cirrhosis, low-grade dysplastic nodule, high-grade dysplastic nodule, early hepatocellular carcinoma, and hepatocellular carcinoma tissue samples compared with healthy samples (FDR < 0.05). Chromosome and bands were listed in chromosomal positions of piRNAs affected expression in liver disease vs healthy samples. The Innermost ring are cirrhosis, then low-grade dysplastic nodule, high-grade dysplastic nodule, early hepatocellular carcinoma, and hepatocellular carcinoma with darker and lighter background colors representing upregulated and downregulated genes respectively.

piRNAs are the largest class of endogenous non-coding RNAs. They have recently been shown to play important biological roles as RNA silencers; forming RNA-protein complexes that are required for both epigenetic and post-transcriptional gene silencing in germ line cells^2,28^. We used piRNA annotation to identify piRNA differential expression in cirrhosis, LGDN, HGDN, eHCC, and HCC groups (Fig. 5A–E, Tables 6–10). We found 75 piRNAs associated with cirrhosis (16 upregulated and 59 downregulated; Fig. 5A, Table 7), 60 piRNAs in LGDN (28 upregulated and 32 downregulated; Fig. 5B, Table 8), 49 piRNAs in HGDN (12 upregulated and 37 downregulated; Fig. 5C, Table 9), 56 piRNAs in eHCC (9 upregulated and 46 downregulated; Fig. 5D, Table 10), and 128 piRNAs in HCC (83 upregulated and 45 downregulated; Fig. 5E, Table 11). The top five differentially upregulated piRNAs in cirrhosis (Table 7) were: piR-32299 (7658-fold), piR-28488 (2105-fold), piR-7239 (1497-fold), piR-5939 (536-fold), and piR-5067 (106-fold); whereas, the top five downregulated were: piR-952 (423-fold), piR-28525 (140-fold), piR-5938 (87-fold), piR-5937 (65-fold), and piR-25780 (49-fold). The top five differentially upregulated piRNAs in LGDN (Table 8) were: piR-32299 (5557-fold), piR-28488 (1804-fold), piR-7239 (1139-fold), piR-5939 (627-fold), and piR-23655 (90-fold). The top five downregulated were: piR-952 (450-fold), piR-12759 (270-fold), piR-820 (101-fold), piR-28525 (82-fold), and piR-5937 (37-fold). The top five differentially upregulated piRNAs in HGDN (Table 9) were: piR-28488 (1013-fold), piR-7239 (798-fold), piR-5939 (281-fold), piR-23655 (96-fold), and piR-6147 (26-fold); with downregulation of: piR-12759 (534-fold), piR-952 (469-fold), piR-25782 (309-fold, top 10, out of 6 were alternative number transcripts of this gene with fold change ranging 94–309), piR-820 (130-fold), and piR-28525 (116-fold). The top five differentially upregulated piRNAs in eHCC (Table 10) were: piR-28488 (1966-fold), piR-7239 (1629-fold), piR-5939 (718-fold), piR-1338 (56-fold), and piR-23786 (18-fold); whereas, the top five downregulated were: piR-952 (383-fold), piR-5937 (121-fold), piR-5938 (108-fold), piR-820 (99-fold), and piR-28525 (81-fold). The top five differentially upregulated piRNAs in HCC (Table 11) were: piR-32299 (4045-fold), piR-23670 (2335-fold), piR-24684 (2220-fold), piR-28488 (1099-fold), and piR-7239 (949-fold). On the other hand, piR-952 (518-fold), piR-820 (78-fold), piR-28525 (58-fold), piR-5938 (57-fold), and piR-5937 (52-fold) were downregulated. Further investigation is needed to evaluate their functions in diseased states, as there is very limited literature available on their functionality.

**Table 7:** Differentially expressed piRNAs in cirrhosis vs healthy patient’s tissue samples (FDR < 0.05).

Feature ID	Chromosome	Fold change	FDR
piR-hsa-32299	1	7658.01	3.35E-08
piR-hsa-28488	6	2105.61	5.75E-13
piR-hsa-7239	2	1496.87	8.21E-12
piR-hsa-5939	12	535.89	6.35E-11
piR-hsa-5067.1	3	106.36	3.35E-08
piR-hsa-5067.4	6	101.29	2.01E-05
piR-hsa-1338	11	59.72	5.59E-10
piR-hsa-26754	5	27.08	1.68E-05
piR-hsa-23786	20	24.42	5.04E-05
piR-hsa-28487	2	16.72	4.06E-05
piR-hsa-3178	20	14.72	3.97E-06
piR-hsa-1823	2	10.13	4.39E-04
piR-hsa-14647	9	5.50	1.90E-02
piR-hsa-27616	19	4.59	1.11E-03
piR-hsa-28319	9	2.84	2.34E-02
piR-hsa-28319.1	9	2.75	4.02E-02
piR-hsa-27283	20	−1.61	4.30E-02
piR-hsa-28875	2	−1.76	3.05E-02
piR-hsa-28875.1	M	−1.81	2.88E-02
piR-hsa-17444	20	−2.00	1.13E-02
piR-hsa-1191	17	−2.00	1.81E-02
piR-hsa-993	5	−2.05	2.57E-02
piR-hsa-11362	21	−2.22	1.14E-02
piR-hsa-20266	5	−2.43	2.20E-02
piR-hsa-20266.1	M	−2.47	1.80E-02
piR-hsa-29218	7	−2.49	2.58E-02
piR-hsa-26039	17	−2.56	1.06E-02
piR-hsa-24672	1	−2.88	8.53E-03
piR-hsa-24672.3	6	−2.89	9.35E-03
piR-hsa-24672.4	6	−2.92	8.53E-03
piR-hsa-24672.1	1	−2.94	8.53E-03
piR-hsa-26686	M	−3.29	4.19E-03
piR-hsa-1207.3	16	−3.73	1.14E-02
piR-hsa-28131.3	3	−3.76	1.29E-02
piR-hsa-28131.1	1	−3.76	1.14E-02
piR-hsa-1207.1	16	−3.76	1.14E-02
piR-hsa-28131	1	−3.76	1.29E-02
piR-hsa-1207.2	16	−3.76	1.15E-02
piR-hsa-28131.2	16	−3.79	1.17E-02
piR-hsa-1207.4	2	−3.80	1.14E-02
piR-hsa-1207	1	−3.81	1.15E-02
piR-hsa-1207.5	5	−3.82	1.14E-02
piR-hsa-28131.4	6	−3.86	1.14E-02
piR-hsa-28877	5	−4.01	1.65E-02
piR-hsa-25447	18	−4.34	1.61E-02
piR-hsa-26685	M	−4.57	1.13E-03
piR-hsa-2117	7	−4.72	5.57E-05
piR-hsa-793	Y	−6.89	1.09E-04
piR-hsa-23317	6	−7.42	5.59E-04
piR-hsa-26681	M	−7.51	2.61E-05
piR-hsa-26684	M	−7.51	2.61E-05
piR-hsa-25780.10	4	−8.41	8.46E-03
piR-hsa-27282	14	−9.65	3.76E-07
piR-hsa-28478	2	−9.94	7.01E-07
piR-hsa-12789	Y	−10.01	1.38E-05
piR-hsa-20757.2	1	−10.06	9.74E-03
piR-hsa-2153	11	−10.23	2.96E-03
piR-hsa-27731	7	−10.91	5.62E-03
piR-hsa-27731.1	M	−11.09	4.53E-03
piR-hsa-11360	9	−11.75	1.20E-04
piR-hsa-20613	7	−12.07	8.94E-06
piR-hsa-9010	17	−12.43	3.80E-06
piR-hsa-26508.6	X	−12.64	2.39E-03
piR-hsa-11361	9	−12.75	1.29E-04
piR-hsa-3200	16	−12.86	1.95E-03
piR-hsa-27493	1	−14.50	2.96E-03
piR-hsa-1177	19	−18.54	3.42E-08
piR-hsa-963	17	−20.24	9.10E-04
piR-hsa-25780.1	1	−47.39	1.05E-03
piR-hsa-25780	1	−47.84	1.47E-03
piR-hsa-25780.9	3	−48.83	1.06E-03
piR-hsa-5937	3	−64.99	3.60E-07
piR-hsa-5938	3	−86.53	6.29E-07
piR-hsa-28525	4	−139.56	6.96E-05
piR-hsa-952	22	−424.84	1.07E-05

**Table 8:** Differentially expressed piRNAs in low-grade dysplastic nodule vs healthy patient’s tissue samples (FDR < 0.05).

Feature ID	Chromosome	Fold change	FDR
piR-hsa-32299	1	5556.66	6.74E-06
piR-hsa-28488	6	1804.05	1.10E-09
piR-hsa-7239	2	1138.69	7.04E-09
piR-hsa-5939	12	626.82	1.10E-09
piR-hsa-23655	5	100.84	8.37E-06
piR-hsa-12488	11	31.18	8.05E-06
piR-hsa-6147	2	29.33	1.29E-03
piR-hsa-12488.1	M	27.82	8.56E-06
piR-hsa-1338	11	17.68	2.04E-08
piR-hsa-23786	20	13.72	1.96E-07
piR-hsa-26131	7	10.87	2.05E-02
piR-hsa-15023.1	M	7.25	2.11E-02
piR-hsa-15023	8	6.80	3.64E-02
piR-hsa-28190	11	6.77	8.81E-04
piR-hsa-3178	20	6.45	4.65E-03
piR-hsa-25783.4	16	5.91	4.11E-03
piR-hsa-27616	19	5.75	2.05E-04
piR-hsa-25783.8	6	5.71	4.65E-03
piR-hsa-25783.1	16	5.63	5.28E-03
piR-hsa-25783.6	2	5.51	5.08E-03
piR-hsa-25783	1	5.44	5.63E-03
piR-hsa-25783.2	16	5.37	4.65E-03
piR-hsa-25783.7	5	5.16	4.11E-03
piR-hsa-25783.3	16	5.09	5.63E-03
piR-hsa-25783.5	17	5.08	6.42E-03
piR-hsa-28390	2	4.23	9.37E-03
piR-hsa-28390.1	8	3.89	2.11E-02
piR-hsa-1823	2	3.07	2.27E-03
piR-hsa-1834	17	−1.77	3.29E-02
piR-hsa-12789	Y	−1.94	2.96E-02
piR-hsa-26685	M	−2.07	3.47E-02
piR-hsa-25447	18	−2.31	4.40E-03
piR-hsa-793	Y	−2.31	1.08E-02
piR-hsa-11361	9	−2.71	1.13E-02
piR-hsa-993	5	−2.72	1.46E-03
piR-hsa-11360	9	−3.13	4.77E-03
piR-hsa-26508.6	X	−3.52	6.43E-03
piR-hsa-26039	17	−3.58	4.56E-04
piR-hsa-9010	17	−3.85	9.29E-04
piR-hsa-27493	1	−4.12	1.06E-02
piR-hsa-26681	M	−4.44	2.95E-04
piR-hsa-26684	M	−4.44	2.95E-04
piR-hsa-2153	11	−4.59	1.34E-02
piR-hsa-27282	14	−5.30	2.67E-05
piR-hsa-17444	20	−6.07	3.45E-06
piR-hsa-3200	16	−6.86	3.79E-05
piR-hsa-1177	19	−7.31	5.33E-05
piR-hsa-2117	7	−7.41	1.76E-06
piR-hsa-28478	2	−9.72	3.84E-07
piR-hsa-25780.9	3	−13.66	3.59E-03
piR-hsa-25780.1	1	−14.57	1.48E-03
piR-hsa-25780	1	−14.89	1.71E-03
piR-hsa-963	17	−16.82	2.62E-07
piR-hsa-5938	3	−36.11	7.78E-07
piR-hsa-20613	7	−36.17	1.10E-09
piR-hsa-5937	3	−36.80	1.76E-06
piR-hsa-28525	4	−81.85	1.90E-09
piR-hsa-820	2	−100.92	4.08E-07
piR-hsa-12759	5	−269.75	2.72E-06
piR-hsa-952	22	−449.82	9.17E-11

**Table 9:** Differentially expressed piRNAs in high-grade dysplastic nodule vs healthy patient’s tissue samples (FDR < 0.05).

Feature ID	Chromosome	Fold change	FDR
piR-hsa-28488	6	1013.41	5.02E-06
piR-hsa-7239	2	791.73	9.22E-06
piR-hsa-5939	12	281.36	2.40E-06
piR-hsa-23655	5	96.41	3.64E-04
piR-hsa-6147	2	25.77	2.93E-02
piR-hsa-1338	11	19.90	4.58E-06
piR-hsa-3178	20	9.12	1.10E-04
piR-hsa-27616	19	6.05	1.41E-03
piR-hsa-25783.4	16	4.10	1.33E-02
piR-hsa-1823	2	4.06	2.63E-03
piR-hsa-25783.6	2	3.78	3.36E-02
piR-hsa-25783.5	17	3.46	3.59E-02
piR-hsa-1834	17	−2.00	4.81E-02
piR-hsa-993	5	−2.74	9.03E-03
piR-hsa-26685	M	−2.79	2.16E-02
piR-hsa-23317	6	−2.93	3.62E-02
piR-hsa-26039	17	−2.98	1.02E-02
piR-hsa-11360	9	−3.62	2.78E-02
piR-hsa-27493	1	−4.62	1.22E-02
piR-hsa-17444	20	−4.65	3.76E-04
piR-hsa-12789	Y	−4.82	3.75E-03
piR-hsa-20757.2	1	−5.60	4.06E-02
piR-hsa-9010	17	−6.88	1.47E-04
piR-hsa-26684	M	−7.15	1.13E-04
piR-hsa-26681	M	−7.15	1.13E-04
piR-hsa-3200	16	−7.38	7.87E-04
piR-hsa-27282	14	−8.31	1.56E-06
piR-hsa-2153	11	−8.92	4.32E-02
piR-hsa-2117	7	−11.59	1.03E-05
piR-hsa-1177	19	−12.56	1.64E-05
piR-hsa-28478	2	−15.42	4.78E-07
piR-hsa-25780.9	3	−16.03	3.03E-02
piR-hsa-25780.1	1	−16.41	4.25E-03
piR-hsa-25780	1	−16.85	2.78E-02
piR-hsa-963	17	−20.25	8.80E-03
piR-hsa-20613	7	−39.75	2.27E-07
piR-hsa-5937	3	−44.83	9.22E-06
piR-hsa-5938	3	−45.11	5.46E-05
piR-hsa-25782.4	16	−94.21	7.87E-04
piR-hsa-25782.3	16	−104.58	3.65E-04
piR-hsa-28525	4	−116.32	2.79E-10
piR-hsa-25782.6	2	−126.18	4.15E-04
piR-hsa-820	2	−130.03	3.65E-04
piR-hsa-25782.8	6	−160.04	5.10E-04
piR-hsa-25782.2	16	−165.67	5.83E-05
piR-hsa-25782.1	16	−181.07	5.46E-05
piR-hsa-25782.5	17	−309.27	3.15E-05
piR-hsa-952	22	−469.05	1.02E-04
piR-hsa-12759	5	−533.60	4.46E-05

**Table 10:** Differentially expressed piRNAs in early hepatocellular carcinoma vs healthy patient’s tissue samples (FDR < 0.05).

Feature ID	Chromosome	Fold change	FDR
piR-hsa-28488	6	1966.20	2.53E-07
piR-hsa-7239	2	1629.10	1.08E-06
piR-hsa-5939	12	718.32	1.08E-06
piR-hsa-1338	11	55.63	4.90E-05
piR-hsa-23786	20	18.06	7.48E-05
piR-hsa-3178	20	12.31	1.78E-03
piR-hsa-1823	2	8.23	2.73E-03
piR-hsa-27616	19	5.29	9.09E-03
piR-hsa-14647	9	4.66	8.69E-03
piR-hsa-793	Y	−1.62	4.58E-02
piR-hsa-11362	21	−1.94	2.81E-02
piR-hsa-26686	M	−2.15	2.02E-02
piR-hsa-26685	M	−2.43	1.24E-02
piR-hsa-993	5	−2.46	1.95E-02
piR-hsa-11361	9	−2.53	8.71E-03
piR-hsa-9010	17	−2.95	8.69E-03
piR-hsa-23317	6	−3.13	1.65E-02
piR-hsa-26039	17	−3.58	4.48E-03
piR-hsa-11360	9	−3.70	6.03E-03
piR-hsa-26508.6	X	−4.56	6.03E-03
piR-hsa-24672.1	1	−4.59	2.73E-03
piR-hsa-24672	1	−4.60	2.83E-03
piR-hsa-27282	14	−4.69	9.52E-04
piR-hsa-24672.3	6	−4.71	2.73E-03
piR-hsa-28131.3	3	−4.74	2.33E-02
piR-hsa-1207.2	16	−4.76	2.33E-02
piR-hsa-1207.1	16	−4.78	2.33E-02
piR-hsa-28131.1	1	−4.79	2.33E-02
piR-hsa-1207.3	16	−4.79	2.26E-02
piR-hsa-24672.4	6	−4.80	2.67E-03
piR-hsa-28131	1	−4.84	2.23E-02
piR-hsa-2117	7	−4.85	1.28E-03
piR-hsa-28131.4	6	−4.87	2.26E-02
piR-hsa-1207.4	2	−4.87	2.26E-02
piR-hsa-1207.5	5	−4.87	2.21E-02
piR-hsa-1207	1	−4.88	2.26E-02
piR-hsa-28131.2	16	−4.90	2.24E-02
piR-hsa-26684	M	−4.99	1.89E-03
piR-hsa-26681	M	−4.99	1.89E-03
piR-hsa-27493	1	−5.54	6.01E-03
piR-hsa-2153	11	−5.95	2.47E-02
piR-hsa-25780.10	4	−8.01	1.83E-02
piR-hsa-1177	19	−9.36	1.92E-04
piR-hsa-28478	2	−10.27	3.44E-05
piR-hsa-3200	16	−13.35	7.06E-04
piR-hsa-20757.2	1	−13.91	6.03E-03
piR-hsa-963	17	−16.72	6.03E-03
piR-hsa-20613	7	−20.96	3.11E-03
piR-hsa-25780.9	3	−24.55	1.08E-02
piR-hsa-25780	1	−24.99	1.14E-02
piR-hsa-25780.1	1	−26.09	8.69E-03
piR-hsa-28525	4	−81.22	1.51E-03
piR-hsa-820	2	−99.09	4.65E-04
piR-hsa-5938	3	−108.31	2.58E-06
piR-hsa-5937	3	−120.72	2.53E-07
piR-hsa-952	22	−382.71	1.07E-09

**Table 11:** Differentially expressed piRNAs in hepatocellular carcinoma vs healthy patient’s tissue samples (FDR < 0.05).

Feature ID	Chromosome	Fold change	FDR
piR-hsa-32299	1	4044.62	9.95E-10
piR-hsa-23670.1	8	2335.40	4.15E-14
piR-hsa-24684.5	5	2219.85	3.09E-08
piR-hsa-24684	1	2072.08	7.08E-10
piR-hsa-28488	6	1098.53	1.93E-15
piR-hsa-7239	2	949.32	2.37E-14
piR-hsa-24684.1	1	761.46	2.93E-08
piR-hsa-5939	12	529.51	2.33E-15
piR-hsa-24684.2	1	375.35	8.29E-08
piR-hsa-24684.3	11	355.01	1.96E-07
piR-hsa-24684.7	5	331.73	3.48E-06
piR-hsa-24684.8	6	241.61	7.96E-07
piR-hsa-24684.4	5	228.88	2.41E-07
piR-hsa-23655	5	106.32	2.38E-06
piR-hsa-24684.6	5	88.62	4.13E-05
piR-hsa-5067.1	3	46.48	1.07E-08
piR-hsa-5067.4	6	44.68	3.71E-06
piR-hsa-12488	11	39.49	1.55E-06
piR-hsa-12488.1	M	35.19	3.88E-06
piR-hsa-6147	2	30.46	1.03E-03
piR-hsa-26131	7	20.96	5.50E-04
piR-hsa-28378.6	6	19.88	4.16E-08
piR-hsa-28374.6	6	19.88	4.16E-08
piR-hsa-1338	11	19.63	4.37E-09
piR-hsa-28378.2	5	18.44	6.16E-08
piR-hsa-28374.2	5	18.44	6.16E-08
piR-hsa-28378	12	18.44	3.01E-08
piR-hsa-28374	12	18.44	3.01E-08
piR-hsa-28374.4	6	18.29	3.90E-08
piR-hsa-28378.4	6	18.29	3.90E-08
piR-hsa-28374.5	6	17.33	3.85E-08
piR-hsa-28378.5	6	17.33	3.85E-08
piR-hsa-28374.1	12	17.22	4.14E-08
piR-hsa-28378.1	12	17.22	4.14E-08
piR-hsa-23786	20	16.93	4.71E-12
piR-hsa-28374.3	6	16.31	4.23E-08
piR-hsa-28378.3	6	16.31	4.23E-08
piR-hsa-26754	5	14.19	8.68E-05
piR-hsa-3178	20	12.26	8.67E-07
piR-hsa-28487	2	9.95	3.28E-08
piR-hsa-15023.1	M	8.68	8.72E-04
piR-hsa-15023	8	8.24	8.71E-04
piR-hsa-28116.3	6	7.13	2.75E-02
piR-hsa-28116.1	17	7.05	3.12E-02
piR-hsa-28116.2	6	6.84	3.45E-02
piR-hsa-28116.7	6	6.73	3.72E-02
piR-hsa-28116.6	6	6.64	4.50E-02
piR-hsa-28116.4	6	6.48	2.58E-02
piR-hsa-28116.8	6	6.36	2.91E-02
piR-hsa-28116.9	6	6.15	3.63E-02
piR-hsa-28190	11	6.13	6.32E-05
piR-hsa-2155	M	5.29	4.29E-02
piR-hsa-27616	19	4.98	5.27E-07
piR-hsa-7193.2	1	4.61	7.08E-03
piR-hsa-7193.3	15	4.49	1.39E-02
piR-hsa-7193.6	2	4.44	1.48E-02
piR-hsa-7193.5	15	4.42	4.51E-03
piR-hsa-7193.1	1	4.33	7.65E-03
piR-hsa-7193.4	15	4.30	7.43E-03
piR-hsa-23619.1	16	4.23	1.70E-02
piR-hsa-25783.4	16	4.20	1.61E-02
piR-hsa-23619.4	6	4.16	1.18E-02
piR-hsa-7193	1	4.12	1.39E-02
piR-hsa-23619	1	4.09	2.36E-02
piR-hsa-25783.6	2	4.05	2.06E-02
piR-hsa-25783.8	6	4.05	1.58E-02
piR-hsa-23619.3	5	4.04	2.88E-02
piR-hsa-25783.1	16	4.03	1.86E-02
piR-hsa-28390	2	3.98	3.29E-03
piR-hsa-25783	1	3.98	2.15E-02
piR-hsa-14647	9	3.87	2.85E-04
piR-hsa-25783.2	16	3.86	2.52E-02
piR-hsa-23619.2	5	3.82	2.04E-02
piR-hsa-28875	2	3.80	2.40E-02
piR-hsa-28875.1	M	3.79	2.91E-02
piR-hsa-25783.3	16	3.75	2.06E-02
piR-hsa-25783.7	5	3.69	2.91E-02
piR-hsa-25783.5	17	3.64	2.86E-02
piR-hsa-28390.1	8	3.62	4.46E-03
piR-hsa-1823	2	3.38	9.34E-04
piR-hsa-1742	1	2.95	8.96E-05
piR-hsa-28319.1	9	1.85	1.01E-02
piR-hsa-28319	9	1.80	1.14E-02
piR-hsa-28382	12	−1.63	4.36E-02
piR-hsa-26686	M	−1.84	2.03E-02
piR-hsa-11362	21	−1.88	3.47E-02
piR-hsa-9010	17	−1.88	1.48E-02
piR-hsa-993	5	−1.90	9.08E-03
piR-hsa-20266	5	−2.29	2.15E-03
piR-hsa-26685	M	−2.35	2.99E-03
piR-hsa-20266.1	M	−2.38	9.82E-04
piR-hsa-1043	4	−2.40	9.82E-04
piR-hsa-24672.3	6	−2.41	6.97E-03
piR-hsa-24672	1	−2.43	5.81E-03
piR-hsa-24672.4	6	−2.44	7.14E-03
piR-hsa-24672.1	1	−2.45	6.07E-03
piR-hsa-23317	6	−2.65	9.61E-03
piR-hsa-28212.2	7	−2.69	6.82E-04
piR-hsa-28212.1	11	−2.70	6.10E-04
piR-hsa-25447	18	−2.82	9.80E-05
piR-hsa-28212	1	−2.82	2.94E-04
piR-hsa-27282	14	−2.99	1.91E-04
piR-hsa-25780.10	4	−3.11	2.88E-02
piR-hsa-26039	17	−3.30	4.84E-05
piR-hsa-17444	20	−3.46	7.18E-06
piR-hsa-11361	9	−3.66	3.52E-03
piR-hsa-26508.6	X	−3.66	2.50E-03
piR-hsa-2153	11	−4.00	2.00E-03
piR-hsa-20757.2	1	−4.30	3.05E-02
piR-hsa-26681	M	−4.36	1.77E-05
piR-hsa-26684	M	−4.36	1.77E-05
piR-hsa-2117	7	−5.03	7.76E-06
piR-hsa-11360	9	−5.05	1.59E-04
piR-hsa-1177	19	−5.27	2.17E-06
piR-hsa-27493	1	−5.27	7.05E-04
piR-hsa-3200	16	−7.10	7.96E-07
piR-hsa-20613	7	−7.45	9.32E-07
piR-hsa-28478	2	−10.34	4.94E-11
piR-hsa-963	17	−11.58	6.23E-08
piR-hsa-25780.1	1	−11.82	2.13E-04
piR-hsa-25780	1	−11.96	2.34E-04
piR-hsa-25780.9	3	−12.16	3.07E-04
piR-hsa-5936	5	−20.70	3.07E-10
piR-hsa-5937	3	−51.29	3.07E-10
piR-hsa-5938	3	−56.84	4.68E-09
piR-hsa-28525	4	−57.64	4.37E-06
piR-hsa-820	2	−78.24	9.30E-06
piR-hsa-952	22	−517.94	1.14E-19

Large data from all five groups was placed in a Venn diagram (Fig. 5F), which showed, 30 piRs were commonly expressed in all groups; whereas six in cirrhosis, seven in HGDN, and 52 in HCC were uniquely expressed (Fig. 5F). Interestingly, we could not determine piRs linked specifically to LGDN and eHCC. There were several piRs commonly expressed between groups; specifically, 14 in LGDN and HCC, 10 in cirrhosis and HCC, 11 in cirrhosis and eHCC, and eight in cirrhosis, HGDN, eHCC, and HCC (Fig. 5F). We also prepared a Circos Plot for comprehensive visualization of differentially expressed piRs, with chromosome number and location (Fig. 5G). After, plugging the data into Circos Plot, we found chromosomes 1, 2, 5, 6, and 22 were the most enriched chromosomes in all five groups (Fig. 5G).

### Differential Expression of lncRNA in Cirrhosis, LGDN, HGDN, eHCC, and HCC Tissue Samples

**Figure 6:**
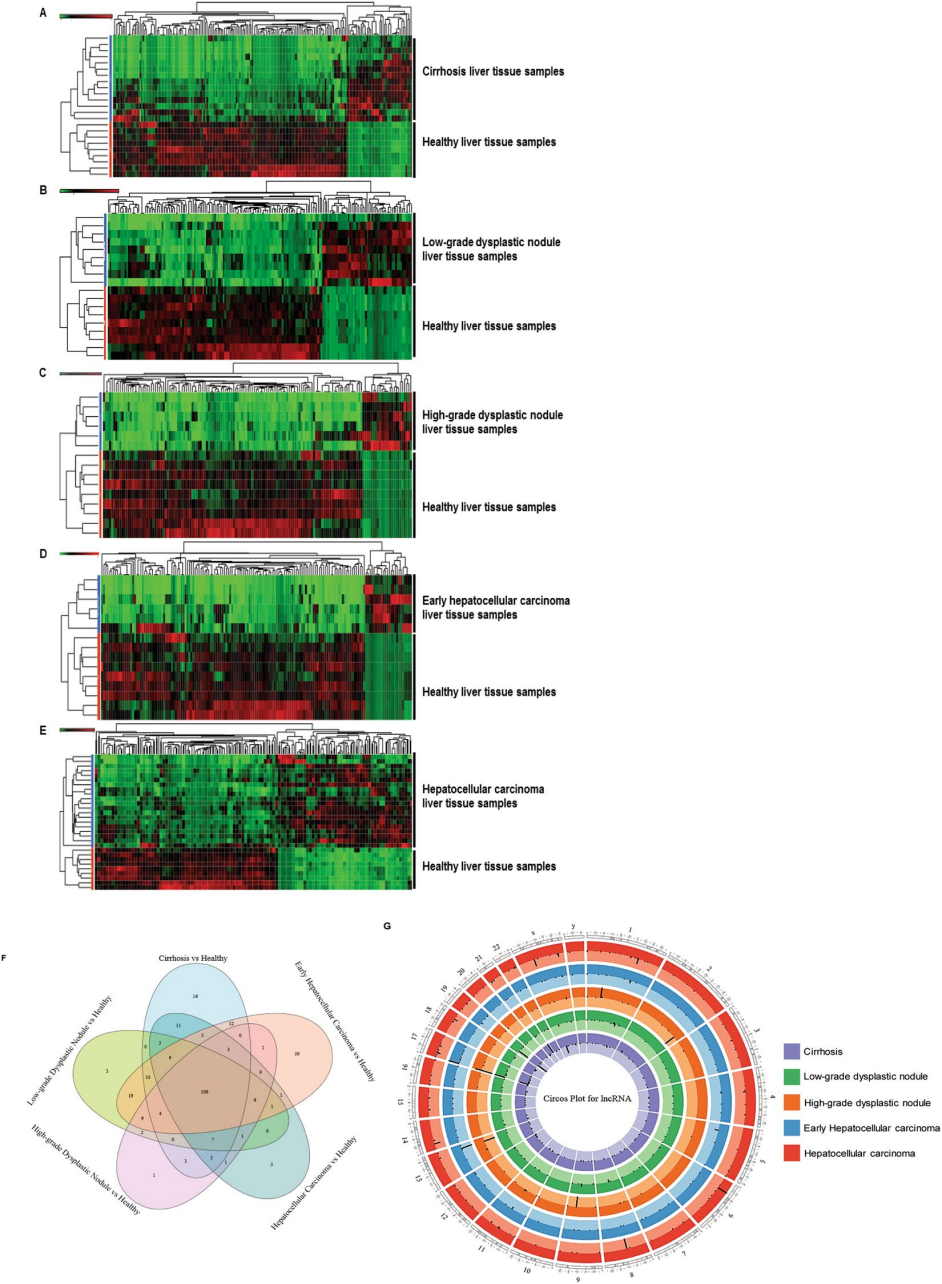
Differential expression of long non-coding RNAs (lncRNAs) in liver tissue samples: Differential expression of lncRNAs were quantified and a heatmap view was prepared (*FDR* < *0.05*) for each disease stage (Cirrhosis, Low-grade dysplastic nodule, High-grade dysplastic nodule, Early stage Hepatocellular carcinoma, and Advanced stage Hepatocellular carcinoma) with healthy control samples (**A**–**E**). All stages of liver disease enriched lncRNAs were summarized by a Venn diagram, which identified 109 lncRNAs commonly expressed in all stages with 39 lncRNAs specifically enriched in early hepatocellular carcinoma and only three lncRNAs enriched in advanced hepatocellular carcinoma (**F**). (**G**) A Circos plot was prepared incorporating all differential expressions of lncRNAs in cirrhosis, low-grade dysplastic nodule, high-grade dysplastic nodule, early hepatocellular carcinoma, and hepatocellular carcinoma tissue samples compared with healthy samples (FDR < 0.05). Chromosome and bands were listed in chromosomal positions of lncRNAs affected expression in liver disease vs healthy samples. The Innermost ring represents cirrhosis, then low-grade dysplastic nodule, high-grade dysplastic nodule, early hepatocellular carcinoma and hepatocellular carcinoma with darker and lighter background colors representing upregulated and downregulated genes respectively.

We further analyzed data for long non-coding RNAs using lncRNA annotation on small RNA sequencing data and calculated differential expression in all five groups (Fig. 6A–E, Tables 12–14, Supplimetery Tables 1 and 2). Our annotation identified 192 lncRNAs in cirrhosis (42 upregulated and 150 downregulated; Fig. 6A, Table 12), 180 lncRNAs in LGDN (53 upregulated and 127 downregulated; Fig. 6B, Supplimentery Table 1), 150 lncRNAs in HGDN (24 upregulated and 126 downregulated; Fig. 6C, Table 13), 160 lncRNAs in eHCC (24 upregulated and 136 downregulated; Fig. 6D, Table 14), and 225 lncRNAs in HCC (96 upregulated and 129 downregulated; Fig. 6E, Supplimentery Table 2). The top five upregulated lncRNAs in cirrhosis (Table 12) were: lnc-C21orf67-10 (23661-fold), lnc-CRK-3 (19138-fold), lnc-FBXO11-7 (5354-fold), lnc-GCNT1-4 (383-fold, 5 various transcripts with fold change range 279–383), and HAGLR:1 (260-fold); whereas, lnc-AC022098.1-1:10 (343-fold), lnc-HSD17B10-3 (231-fold), lnc-NEDD4L-1 (42-fold), GAS5:43 (41-fold), and lnc-CTD-2144E22.5.1-20 (36-fold) were downregulated. The top five upregulated lncRNAs in LGDN (Supplimentery Table 1) were: lnc-CRK-3 (1830-fold), GCNT1-4 (355-fold, five various transcripts with fold change range 339–383), lnc-ADCY10-1 (271-fold), lnc-UBC-3 (237-fold), and lnc-TRIM27-18 (172-fold). The top five downregulated were: lnc-AC022098.1-1:10 (756-fold), SNHG6:15 (144-fold), GAS5 (124-fold, three transcripts), lnc-HSD17B10-3 (104-fold), and lnc-CCNB1IP1-1 (103-fold). The top five upregulated lncRNAs in HGDN (Table 13) were: GCNT1-4 (307-fold, top 5 various transcripts with fold change range 295–307), lnc-UBC-3 (293-fold), lnc-TRIM27-18 (238-fold), LNC00273:8 (34-fold), and lnc-FNBP1L-2 (18-fold) and the top five downregulated were: lnc-AC022098.1-1:10 (1603-fold), GAS5 (685-fold, four transcripts with fold change ranging from 51–685), SNHG6:15 (409-fold), lnc-HSD17B10-3 (105-fold), and lnc-CTD-2144E22.5.1-20 (89-fold). The top five upregulated lncRNAs in eHCC (Table 14) were: lnc-CRK-3 (6385-fold), GCNT1-4 (290-fold, continue 5 various transcripts with fold change range 237–290), HAGLR (251-fold), lnc-UBC-3 (202-fold), and lnc-TRIM27-18 (171-fold); whereas the top five downregulated were: lnc-MBNL2-3 (1457-fold), LINC01021:16 (1317-fold), GAS5 (815-fold, multiple transcript types), SNHG1:60 (694-fold), and lnc-AC022098.1-1:10 (577-fold). The top five upregulated lncRNAs in HCC (Supplimentery Table 2) were: lnc-CCDC167-2 (1 million-fold), lnc-TPTE-3 (18378-fold), lnc-C21orf67-10 (9271-fold), lnc-TMEM8A-1 (6308-fold), and lnc-CRK-3 (3027-fold), with downregulation seen in: lnc-SNHG6:15 (99-fold), HSD17B10-3 (65-fold), lnc-CCNB1IP1-1 (50-fold), GAS5 (45-fold, multiple transcripts), and lnc-ARHGEF6-4 (28-fold). Further investigation is needed to determine their importance in HCC development.

**Table 12:** Differentially expressed lncRNAs in cirrhosis vs healthy patient’s tissue samples (FDR < 0.05).

lncRNA ID	Chromosome	Fold change	FDR
lnc-C21orf67-10:1	21	23661.18	1.25E-09
lnc-CRK-3:2	17	19137.71	4.54E-08
lnc-FBXO11-7:1	2	5354.15	1.25E-09
lnc-GCNT1-4:6	9	382.70	4.77E-08
lnc-GCNT1-4:5	9	320.01	8.81E-07
lnc-GCNT1-4:2	9	284.32	5.92E-05
lnc-GCNT1-4:3	9	284.07	4.24E-05
lnc-GCNT1-4:4	9	278.80	8.29E-05
HAGLR:1	2	259.55	2.06E-06
HAGLR:31	2	259.36	2.06E-06
lnc-ADCY10-1:1	1	199.46	3.12E-06
lnc-UBC-3:1	12	146.21	8.81E-07
lnc-TRIM27-18:1	6	68.97	7.70E-04
lnc-AC106017.1.1-2:1	17	52.61	1.30E-09
lnc-FNBP1L-2:1	1	36.38	3.18E-05
lnc-FCGR3A-3:1	1	30.45	3.64E-08
lnc-ZNF391-5:2	6	26.39	2.34E-07
lnc-AC102948.1.1-4:1	17	25.97	3.76E-07
lnc-ECHDC1-1:1	6	16.78	4.18E-04
lnc-MAP1LC3B-7:1	16	16.24	4.41E-06
lnc-PRRC2C-5:1	1	15.40	5.83E-05
lnc-C6orf100-12:1	6	15.25	9.73E-06
LIMD1-AS1:6	3	14.15	1.96E-05
lnc-GNGT2-1:2	17	14.13	5.72E-04
lnc-GPR39-7:2	2	11.30	1.17E-02
SNHG1:34	11	8.88	5.63E-05
lnc-TMPO-1:1	12	6.71	4.40E-03
lnc-HIST1H2BI-2:2	6	6.70	8.37E-04
lnc-NARF-2:2	17	6.53	7.74E-04
lnc-SAG-4:1	2	6.35	6.94E-03
lnc-TRIM7-2:5	5	5.95	7.95E-03
lnc-PARVG-4:1	22	4.65	1.98E-03
MIR22HG:35	17	4.37	4.94E-02
MIR22HG:18	17	4.37	4.94E-02
MIR22HG:24	17	4.32	4.77E-02
lnc-GRAP-1:1	17	4.27	4.96E-04
lnc-AC007952.2.1-2:1	17	4.26	5.31E-04
MIR22HG:47	17	4.25	4.87E-02
lnc-AC007952.1.1-3:1	17	4.21	4.04E-04
lnc-AC007952.2-2:3	17	4.16	4.51E-04
lnc-AC007952.2-2:1	17	4.14	4.59E-04
lnc-METTL12-1:2	11	1.97	3.70E-02
DLEU2:20	13	−1.17	4.93E-02
GAS5:42	1	−1.31	3.12E-02
GAS5:83	1	−1.32	3.08E-02
lnc-GPC3-1:1	X	−1.49	2.81E-02
lnc-AC027763.2.1-1:2	17	−1.56	2.42E-02
lnc-ZFAT-1:6	8	−1.57	4.35E-02
lnc-MON2-2:8	12	−1.58	3.34E-02
lnc-MON2-2:10	12	−1.60	3.08E-02
lnc-MON2-2:2	12	−1.60	3.08E-02
lnc-KNG1-2:6	3	−1.61	3.90E-02
lnc-COPG2-3:1	7	−1.62	4.77E-02
SNHG16:42	17	−1.74	3.16E-02
lnc-TPTE-3:4	21	−1.86	8.02E-03
lnc-CTR9-1:2	11	−2.06	9.05E-03
lnc-FLOT2-1:1	17	−2.07	3.17E-02
lnc-SNURF-1:73	15	−2.14	2.67E-02
lnc-C9orf100-1:1	9	−2.15	4.22E-03
lnc-IRS4-2:1	X	−2.22	4.25E-03
lnc-SNURF-1:17	15	−2.50	8.46E-03
lnc-HIATL1-20:1	9	−2.55	7.27E-03
lnc-SNAPC5-4:3	15	−2.57	5.11E-03
lnc-TRIM52-2:1	5	−2.61	3.17E-03
lnc-RPL13A-1:5	19	−2.65	3.05E-03
lnc-AC106017.1.1-2:2	17	−2.70	5.11E-03
LINC00273:1	16	−2.71	1.04E-03
LINC00273:11	16	−2.77	3.02E-03
lnc-GRAP-1:2	17	−2.83	3.64E-02
lnc-TRIM59-2:1	3	−2.96	1.45E-03
MIR210HG:7	11	−2.96	7.50E-03
SNHG21:29	15	−3.01	2.42E-02
lnc-GCNT1-4:1	9	−3.04	3.40E-03
lnc-PLA2G1B-2:3	12	−3.13	3.40E-03
FTX:29	X	−3.18	8.08E-03
lnc-HEPH-1:1	X	−3.29	2.27E-02
lnc-HES7-1:1	17	−3.29	1.61E-02
lnc-TTLL10-3:9	1	−3.42	3.14E-02
lnc-CCNB1IP1-1:4	14	−3.42	4.32E-02
lnc-ERAL1-1:1	17	−3.43	1.65E-02
LINC-PINT:28	7	−3.46	7.20E-03
lnc-COL4A5-3:1	X	−3.46	6.64E-04
LINC-PINT:29	7	−3.48	4.72E-03
lnc-KIF20B-3:1	10	−3.54	2.92E-03
lnc-FGA-1:1	4	−3.57	2.25E-04
SNHG1:44	11	−3.57	1.08E-03
SNHG1:4	11	−3.57	1.08E-03
SNHG19:3	16	−3.63	5.35E-04
lnc-RP4-695O20__B.10.1-3:10	22	−3.75	2.66E-03
lnc-GPR39-10:3	2	−3.75	1.98E-04
lnc-RP4-695O20__B.10.1-3:9	22	−3.75	2.66E-03
lnc-RP4-695O20__B.10.1-3:12	22	−3.75	2.66E-03
SNHG22:3	18	−3.82	2.20E-04
UBA6-AS1:26	4	−3.90	4.16E-04
SNHG5:47	6	−3.92	6.02E-04
SNHG5:5	6	−3.92	6.02E-04
lnc-CTD-2144E22.5.1-19:1	16	−3.93	2.31E-02
lnc-VMP1-3:3	17	−4.24	2.10E-03
DNM3OS:3	1	−4.27	2.92E-03
LINC00472:10	6	−4.33	2.57E-03
lnc-SLC15A4-24:1	12	−4.41	9.30E-06
lnc-AL669831.1-12:2	1	−4.46	2.20E-04
lnc-C11orf54-1:1	11	−4.50	1.65E-02
lnc-AC007952.1.1-3:2	17	−4.62	8.97E-03
GAS5:68	1	−4.64	5.35E-04
lnc-CUL1-5:1	7	−4.68	6.37E-04
lnc-AL669831.1-14:1	1	−4.78	2.26E-02
lnc-TSPY10-14:1	Y	−4.78	4.41E-05
lnc-KLK1-2:3	19	−4.90	2.25E-04
lnc-KIF2C-2:1	1	−5.12	2.17E-04
lnc-C19orf57-6:5	19	−5.31	1.09E-03
lnc-PDIA4-1:1	7	−5.34	4.07E-04
lnc-GPR39-7:1	2	−5.46	2.64E-05
lnc-LRR1-1:1	14	−5.49	1.21E-04
MIR503HG:16	X	−5.53	1.18E-02
lnc-LRR1-1:4	14	−5.54	1.10E-04
lnc-HIST1H2AH-5:1	6	−5.57	5.50E-03
lnc-SLC25A30-3:1	13	−5.59	1.55E-02
lnc-GPR39-10:2	2	−5.61	6.27E-06
MIR503HG:17	X	−5.62	1.01E-02
lnc-FGG-2:1	4	−5.63	5.83E-05
lnc-NEMF-1:4	14	−5.67	1.17E-04
lnc-AC007952.2.1-2:2	17	−5.92	7.20E-03
MIR193BHG:18	16	−6.01	1.27E-02
lnc-AFP-1:2	4	−6.14	1.85E-05
lnc-AFP-3:1	4	−6.17	8.71E-06
lnc-ANKRD34B-4:2	5	−6.22	1.51E-04
lnc-AUTS2-6:1	7	−6.55	1.28E-06
lnc-KNG1-2:8	3	−6.65	2.69E-04
MIR99AHG:51	21	−6.83	1.98E-04
MIR99AHG:50	21	−6.83	1.98E-04
MIR99AHG:63	21	−6.83	1.98E-04
MIR99AHG:42	21	−6.84	1.98E-04
lnc-DUOX1-2:1	15	−6.94	2.92E-03
lnc-DUOXA1-2:1	15	−6.97	2.92E-03
lnc-DUOXA1-1:1	15	−7.02	2.71E-03
lnc-ZBTB37-2:1	1	−7.11	4.82E-04
GAS5:16	1	−7.16	7.55E-03
lnc-SYT10-3:5	12	−7.18	1.04E-05
LINC01138:11	1	−7.26	5.98E-06
lnc-RABGGTB-1:6	1	−7.59	5.68E-05
lnc-HNRNPA2B1-10:4	7	−7.75	1.47E-04
lnc-AC006156.1-11:1	Y	−7.76	9.35E-06
lnc-DAO-3:1	12	−7.76	1.85E-05
lnc-CFH-2:1	1	−7.78	1.66E-06
lnc-VSTM5-1:13	11	−8.06	1.98E-04
lnc-SNURF-1:2	15	−8.07	3.64E-03
lnc-AUTS2-6:2	7	−8.45	9.30E-06
lnc-NEK8-2:1	17	−8.52	4.33E-05
SNHG1:25	11	−8.66	4.22E-03
LRRC75A-AS1:49	17	−8.93	1.04E-04
MIR17HG:6	13	−9.59	9.21E-05
lnc-SPG7-2:3	16	−9.70	2.94E-05
ZFAS1:23	20	−9.84	4.68E-05
lnc-AK3-1:1	9	−9.89	2.69E-04
lnc-TPTE-3:5	21	−9.93	2.64E-02
SNHG1:12	11	−10.01	3.40E-03
lnc-ZNF169-7:2	9	−10.29	2.64E-04
lnc-APAF1-3:1	12	−10.43	5.36E-03
lnc-SNAPC5-4:1	15	−10.61	1.26E-05
lnc-SNAPC5-4:2	15	−10.74	9.76E-03
lnc-SLC3A2-6:1	11	−11.22	7.37E-05
lnc-SNURF-1:92	15	−12.62	2.89E-05
lnc-SNURF-1:95	15	−12.62	2.96E-05
SNHG1:1	11	−13.61	6.63E-06
lnc-AFP-2:1	4	−14.05	6.73E-07
SNHG1:59	11	−14.65	3.37E-03
lnc-ZNF169-7:7	9	−15.03	1.52E-03
lnc-VSTM5-1:10	11	−16.65	1.75E-03
lnc-MOS-1:2	8	−17.57	2.77E-06
lnc-HAUS5-3:2	19	−18.31	2.94E-07
lnc-HAUS5-3:1	19	−18.35	2.94E-07
lnc-MINA-3:5	3	−19.52	1.41E-03
SNHG8:14	4	−19.53	1.11E-03
lnc-RPL17-2:4	18	−21.13	3.79E-05
lnc-MYO16-7:1	13	−21.97	3.76E-07
lnc-CRKL-2:1	22	−22.43	4.38E-07
lnc-VSTM5-1:7	11	−23.84	5.36E-04
LINC00910:16	17	−25.81	6.63E-06
LINC00910:1	17	−26.20	5.76E-06
lnc-ARHGEF6-4:1	X	−28.71	4.77E-08
MIR17HG:5	13	−28.92	1.14E-03
LRRC75A-AS1:41	17	−29.36	9.83E-05
lnc-CCNB1IP1-1:2	14	−31.52	1.91E-05
lnc-TPTE-3:9	21	−33.36	7.98E-04
LRRC75A-AS1:36	17	−34.20	2.52E-06
lnc-TMEM132C-11:1	12	−34.56	1.45E-05
lnc-CTD-2144E22.5.1-20:1	16	−35.71	1.21E-03
GAS5:43	1	−41.26	2.89E-05
lnc-NEDD4L-1:6	18	−41.69	1.28E-06
lnc-HSD17B10-3:1	X	−230.89	1.85E-05
lnc-AC022098.1-1:10	19	−343.09	2.34E-07

**Table 13:** Differentially expressed lncRNAs in high-grade dysplastic nodule vs healthy patient’s tissue samples (FDR < 0.05).

**lncRNA ID**	**Chromosome**	**Fold change**	**FDR**
lnc-GCNT1-4:6	9	306.64	2.76E-08
lnc-GCNT1-4:5	9	305.75	2.69E-08
lnc-GCNT1-4:2	9	302.98	4.40E-08
lnc-GCNT1-4:3	9	301.19	4.40E-08
lnc-GCNT1-4:4	9	295.48	4.40E-08
lnc-UBC-3:1	12	292.71	1.48E-05
lnc-TRIM27-18:1	6	238.48	1.25E-03
LINC00273:8	16	33.66	2.40E-02
lnc-FNBP1L-2:1	1	17.70	1.66E-02
lnc-GNGT2-1:2	17	16.60	6.70E-05
lnc-SDHC-3:1	1	15.91	4.38E-03
lnc-PRRC2C-5:1	1	13.19	3.82E-05
lnc-TRIM7-2:5	5	13.02	1.95E-04
lnc-OR2V2-3:1	5	12.02	2.11E-02
lnc-ZNF391-5:2	6	11.70	7.69E-03
lnc-IL17RB-3:1	3	8.65	2.54E-02
LIMD1-AS1:6	3	7.10	1.81E-03
MIR22HG:47	17	5.12	6.22E-05
MIR22HG:24	17	5.12	6.10E-05
MIR22HG:18	17	5.08	6.47E-05
MIR22HG:35	17	5.08	6.47E-05
lnc-ZNF133-3:1	20	4.65	8.10E-03
lnc-AL669831.1-11:4	1	4.19	2.94E-03
lnc-SAG-4:1	2	3.83	2.18E-03
lnc-RRBP1-3:1	20	−1.51	4.91E-02
lnc-REG3G-6:1	2	−1.75	3.84E-02
lnc-RP4-695O20__B.10.1-3:10	22	−1.93	4.56E-02
lnc-RP4-695O20__B.10.1-3:9	22	−1.93	4.60E-02
lnc-RP4-695O20__B.10.1-3:12	22	−1.93	4.55E-02
lnc-MON2-2:8	12	−1.97	4.43E-02
lnc-MON2-2:10	12	−1.97	4.31E-02
lnc-MON2-2:2	12	−1.98	4.33E-02
lnc-HNRNPA2B1-10:4	7	−2.04	4.08E-02
lnc-IRS4-2:1	X	−2.04	1.37E-02
MIR210HG:7	11	−2.05	3.49E-02
SNHG16:42	17	−2.14	9.50E-03
LINC00273:11	16	−2.24	1.39E-02
lnc-SLC25A30-3:1	13	−2.28	4.56E-02
lnc-NEMF-1:4	14	−2.32	3.84E-02
lnc-FPR2-1:35	19	−2.67	2.60E-02
DNM3OS:3	1	−2.68	1.35E-02
lnc-AL031590.1-1:2	22	−2.78	1.45E-03
lnc-C19orf57-6:5	19	−2.88	1.50E-02
lnc-TPTE-3:4	21	−2.91	2.61E-04
lnc-CUL1-5:1	7	−2.97	6.01E-03
lnc-HARS-1:1	5	−3.04	1.10E-02
lnc-AL669831.1-14:1	1	−3.06	1.88E-03
lnc-C9orf100-1:1	9	−3.09	9.39E-04
lnc-AL669831.1-12:2	1	−3.20	1.09E-03
lnc-ANKRD34B-4:2	5	−3.22	3.65E-03
lnc-TRIM52-2:1	5	−3.26	1.35E-03
SNHG1:4	11	−3.33	1.94E-02
SNHG1:44	11	−3.33	1.94E-02
lnc-CFH-2:1	1	−3.33	1.16E-02
lnc-FGG-2:1	4	−3.34	1.46E-03
lnc-ZNF169-7:2	9	−3.37	2.81E-02
SNHG22:3	18	−3.38	1.46E-03
lnc-GMEB1-1:5	1	−3.40	1.23E-02
lnc-SNAPC5-4:3	15	−3.46	7.41E-03
lnc-RPL13A-1:5	19	−3.51	4.33E-04
lnc-TMC2-1:1	20	−3.58	1.49E-03
lnc-SNURF-1:73	15	−3.82	4.76E-03
SNHG5:5	6	−3.92	1.37E-03
SNHG5:47	6	−3.92	1.37E-03
lnc-LRR1-1:1	14	−3.96	4.88E-03
lnc-LRR1-1:4	14	−4.00	4.66E-03
MIR17HG:6	13	−4.09	3.17E-03
UBA6-AS1:26	4	−4.13	3.02E-04
lnc-DAO-3:1	12	−4.13	1.95E-04
MIR99AHG:42	21	−4.40	3.20E-04
MIR99AHG:63	21	−4.41	3.05E-04
MIR99AHG:51	21	−4.41	2.99E-04
MIR99AHG:50	21	−4.41	3.07E-04
lnc-AK3-1:1	9	−4.52	1.49E-02
lnc-MINA-3:5	3	−4.58	4.83E-02
lnc-AFP-1:2	4	−4.61	1.20E-04
lnc-AFP-3:1	4	−4.73	6.18E-05
GAS5:68	1	−4.82	8.89E-04
lnc-SYT10-3:5	12	−4.83	2.39E-04
lnc-AC106017.1.1-2:2	17	−5.05	1.62E-04
ZFAS1:23	20	−5.13	4.77E-04
lnc-SLC15A4-24:1	12	−5.27	9.67E-05
lnc-VSTM5-1:10	11	−5.28	4.80E-03
GAS5:16	1	−5.32	8.40E-03
lnc-SPG7-2:3	16	−5.39	2.51E-03
MIR503HG:16	X	−5.40	3.87E-03
lnc-TRIM59-2:1	3	−5.45	6.70E-05
SNHG8:14	4	−5.54	4.91E-02
MIR503HG:17	X	−5.57	3.20E-03
lnc-TSPY10-14:1	Y	−5.60	1.12E-05
lnc-TTLL10-3:9	1	−5.63	5.54E-03
lnc-SLC3A2-6:1	11	−5.63	9.50E-03
LINC00273:1	16	−5.75	3.85E-05
lnc-SNURF-1:2	15	−5.95	2.64E-03
lnc-TPTE-3:5	21	−5.99	3.08E-02
lnc-GPR39-10:3	2	−6.00	8.60E-06
lnc-KIF2C-2:1	1	−6.04	2.16E-04
lnc-RABGGTB-1:6	1	−6.38	4.67E-04
lnc-AFP-2:1	4	−6.72	3.18E-05
SNHG1:12	11	−6.73	9.93E-03
SNHG19:3	16	−6.74	1.06E-05
lnc-DNAJC12-1:1	10	−6.82	1.71E-02
lnc-GPR39-7:1	2	−6.83	4.56E-06
lnc-COL4A5-3:1	X	−6.87	2.97E-04
lnc-ZBTB37-2:1	1	−7.10	3.12E-03
lnc-CTD-2144E22.5.1-19:1	16	−7.13	5.07E-04
lnc-SERHL2-4:4	22	−7.33	6.07E-03
lnc-SERHL2-4:2	22	−7.33	6.07E-03
lnc-KLK1-2:3	19	−7.62	3.05E-04
lnc-APAF1-3:1	12	−7.94	3.07E-04
lnc-AUTS2-6:1	7	−8.38	1.07E-06
lnc-AC007952.2.1-2:2	17	−8.55	1.44E-02
lnc-GPR39-10:2	2	−8.80	1.53E-07
lnc-CRKL-2:1	22	−8.88	1.21E-04
lnc-HAUS5-3:2	19	−9.02	8.35E-05
lnc-HAUS5-3:1	19	−9.03	8.47E-05
lnc-MOS-1:2	8	−9.24	2.95E-04
LRRC75A-AS1:49	17	−9.48	1.37E-03
lnc-MYO16-7:1	13	−9.67	4.56E-06
lnc-SNURF-1:92	15	−10.05	2.32E-04
lnc-AUTS2-6:2	7	−10.05	3.06E-06
lnc-TPTE-3:9	21	−10.19	8.25E-03
lnc-AC006156.1-11:1	Y	−10.27	6.52E-05
lnc-SNURF-1:95	15	−10.49	2.06E-04
lnc-SNAPC5-4:2	15	−10.58	2.81E-04
lnc-SNAPC5-4:1	15	−10.70	2.78E-04
MIR17HG:5	13	−10.86	5.80E-04
lnc-GCNT1-4:1	9	−11.55	1.95E-04
lnc-RPL17-2:4	18	−13.78	2.45E-04
lnc-TMEM132C-11:1	12	−14.40	1.56E-03
SNHG1:59	11	−14.45	6.70E-05
SNHG1:1	11	−15.41	6.28E-05
LINC00910:1	17	−16.17	9.82E-04
LINC00910:16	17	−16.27	9.82E-04
LINC01138:11	1	−16.27	4.28E-07
lnc-NEDD4L-1:6	18	−16.89	8.47E-05
lnc-VSTM5-1:7	11	−19.43	3.30E-04
LRRC75A-AS1:41	17	−33.16	6.44E-03
lnc-ARHGEF6-4:1	X	−34.34	4.40E-08
lnc-MINA-3:4	3	−36.74	2.35E-03
LRRC75A-AS1:36	17	−41.92	1.37E-04
lnc-CCNB1IP1-1:2	14	−48.86	2.60E-04
GAS5:43	1	−50.97	1.92E-04
lnc-CTD-2144E22.5.1-20:1	16	−89.06	2.07E-03
lnc-HSD17B10-3:1	X	−104.76	7.69E-04
GAS5:41	1	−237.50	6.47E-05
SNHG6:15	8	−408.63	1.61E-04
GAS5:7	1	−685.38	1.16E-05
GAS5:72	1	−685.38	1.16E-05
lnc-AC022098.1-1:10	19	−1602.87	1.29E-05

**Table 14:** Differentially expressed lncRNAs in early hepatocellular carcinoma vs healthy patient’s tissue samples (FDR < 0.05).

lncRNA ID	Chromosome	Fold change	FDR
lnc-CRK-3:2	17	6384.96	9.03E-05
lnc-GCNT1-4:6	9	289.82	1.66E-08
lnc-GCNT1-4:5	9	269.34	1.66E-08
HAGLR:1	2	251.27	3.02E-03
lnc-GCNT1-4:2	9	240.21	1.33E-07
lnc-GCNT1-4:3	9	237.82	1.33E-07
lnc-GCNT1-4:4	9	237.45	1.33E-07
lnc-UBC-3:1	12	202.61	4.21E-05
lnc-TRIM27-18:1	6	170.61	4.91E-03
lnc-AC106017.1.1-2:1	17	51.74	2.12E-05
lnc-FNBP1L-2:1	1	24.51	2.18E-03
lnc-GNGT2-1:2	17	23.07	4.17E-04
LINC00273:8	16	20.55	1.80E-02
lnc-AC102948.1.1-4:1	17	15.69	8.11E-05
LIMD1-AS1:6	3	15.67	3.53E-04
lnc-PRRC2C-5:1	1	15.57	1.02E-03
lnc-ZNF391-5:2	6	14.67	1.26E-03
lnc-GPR39-7:2	2	11.36	1.21E-03
lnc-ZNF133-3:1	20	5.79	3.20E-02
lnc-SAG-4:1	2	4.05	1.22E-02
MIR22HG:35	17	2.84	1.32E-02
MIR22HG:18	17	2.84	1.32E-02
MIR22HG:24	17	2.84	1.16E-02
MIR22HG:47	17	2.83	1.19E-02
FTX:29	X	−1.01	4.72E-02
lnc-SLC25A30-3:1	13	−1.61	4.56E-02
LINC-PINT:28	7	−1.66	4.39E-02
lnc-VMP1-3:3	17	−1.70	3.20E-02
LINC-PINT:29	7	−1.74	3.44E-02
lnc-C9orf100-1:1	9	−1.90	3.30E-02
lnc-RPL13A-1:5	19	−1.97	3.73E-02
LINC00472:10	6	−1.98	2.50E-02
lnc-SNURF-1:17	15	−1.99	4.63E-02
SNHG16:42	17	−2.01	2.10E-02
lnc-KIF20B-3:1	10	−2.02	3.04E-02
lnc-ZNF169-7:7	9	−2.07	2.64E-02
MIR193BHG:18	16	−2.12	2.17E-02
lnc-FPR2-1:35	19	−2.27	2.20E-02
LINC00324:3	17	−2.30	1.37E-02
lnc-SNAPC5-4:3	15	−2.35	2.63E-02
lnc-SLC15A4-24:1	12	−2.36	1.32E-02
lnc-RP4-695O20__B.10.1-3:9	22	−2.36	1.01E-02
lnc-RP4-695O20__B.10.1-3:12	22	−2.37	9.86E-03
lnc-RP4-695O20__B.10.1-3:10	22	−2.37	9.86E-03
SNHG1:4	11	−2.41	1.50E-02
SNHG1:44	11	−2.41	1.50E-02
lnc-MON2-2:8	12	−2.43	1.19E-02
lnc-PLA2G1B-2:3	12	−2.45	2.44E-02
lnc-MON2-2:10	12	−2.45	1.10E-02
lnc-MON2-2:2	12	−2.45	1.13E-02
lnc-SYT10-3:5	12	−2.46	1.38E-02
lnc-NEMF-1:4	14	−2.49	1.75E-02
lnc-CUL1-5:1	7	−2.58	1.09E-02
lnc-TRIM52-2:1	5	−2.62	9.07E-03
lnc-ANKRD34B-4:2	5	−2.68	9.57E-03
MIR210HG:7	11	−2.75	1.99E-02
LINC00273:1	16	−2.89	1.49E-03
UBA6-AS1:26	4	−2.97	3.85E-03
lnc-SNURF-1:73	15	−3.03	1.67E-02
lnc-LRR1-1:1	14	−3.07	5.99E-03
lnc-LRR1-1:4	14	−3.10	5.70E-03
lnc-TRIM59-2:1	3	−3.19	7.10E-04
lnc-CRKL-2:1	22	−3.22	3.83E-02
lnc-TSPY10-14:1	Y	−3.29	2.12E-03
lnc-NEK8-2:1	17	−3.33	8.39E-03
SNHG19:3	16	−3.36	1.54E-03
lnc-GCNT1-4:1	9	−3.38	7.74E-03
lnc-ZNF169-7:2	9	−3.49	4.90E-03
lnc-CFH-2:1	1	−3.52	3.31E-03
lnc-AC106017.1.1-2:2	17	−3.53	2.10E-03
lnc-HNRNPA2B1-10:4	7	−3.57	6.41E-03
lnc-MINA-3:5	3	−3.63	2.18E-02
lnc-HEPH-1:1	X	−3.70	1.28E-02
lnc-AUTS2-6:1	7	−3.72	2.54E-04
lnc-GPR39-10:3	2	−3.79	2.22E-04
lnc-KNG1-2:8	3	−3.80	7.11E-03
SNHG5:47	6	−3.83	1.35E-03
SNHG5:5	6	−3.83	1.35E-03
lnc-TTLL10-3:9	1	−3.88	1.04E-02
lnc-GPR39-7:1	2	−3.91	6.00E-04
lnc-FGG-2:1	4	−3.97	1.01E-03
SNHG22:3	18	−4.08	2.79E-03
lnc-KLK1-2:3	19	−4.24	5.23E-03
lnc-DUOX1-2:1	15	−4.38	4.95E-02
lnc-CTD-2144E22.5.1-19:1	16	−4.39	5.42E-03
lnc-DUOXA1-1:1	15	−4.44	4.91E-02
lnc-COL4A5-3:1	X	−4.50	2.74E-02
lnc-C19orf57-6:5	19	−4.51	3.02E-03
lnc-C11orf54-1:1	11	−4.55	1.20E-03
MIR17HG:6	13	−4.58	2.26E-03
lnc-KIF2C-2:1	1	−4.65	1.27E-03
ZFAS1:23	20	−4.80	1.49E-03
GAS5:68	1	−4.82	1.03E-03
lnc-GPR39-10:2	2	−4.89	8.14E-05
lnc-FLOT2-1:1	17	−4.95	1.70E-02
lnc-AL669831.1-14:1	1	−4.99	2.43E-04
lnc-VSTM5-1:13	11	−5.01	3.83E-03
lnc-TPTE-3:9	21	−5.16	7.51E-03
DNM3OS:3	1	−5.18	2.18E-03
lnc-AL669831.1-12:2	1	−5.19	1.30E-04
lnc-MSH3-2:1	5	−5.54	1.78E-02
lnc-VSTM5-1:10	11	−5.73	1.71E-02
lnc-AUTS2-6:2	7	−5.75	7.10E-04
lnc-AFP-3:1	4	−5.79	4.31E-05
lnc-HAUS5-3:2	19	−5.89	1.21E-03
lnc-HAUS5-3:1	19	−5.90	1.23E-03
SNHG8:14	4	−6.16	1.01E-02
lnc-AK3-1:1	9	−6.43	2.40E-03
lnc-ZBTB37-2:1	1	−6.45	4.27E-03
lnc-AC006156.1-11:1	Y	−6.46	7.10E-04
lnc-AFP-1:2	4	−6.56	5.13E-05
lnc-MYO16-7:1	13	−6.62	5.66E-04
lnc-SLC3A2-6:1	11	−6.69	6.61E-03
lnc-RABGGTB-1:6	1	−6.84	5.72E-04
SNHG1:25	11	−7.00	1.37E-02
lnc-AC007952.2.1-2:2	17	−7.51	1.91E-03
lnc-MOS-1:2	8	−7.79	6.47E-04
LINC01138:11	1	−7.81	1.59E-04
MIR503HG:16	X	−7.92	1.14E-03
lnc-APAF1-3:1	12	−7.99	4.93E-04
MIR17HG:5	13	−8.00	1.03E-03
MIR503HG:17	X	−8.05	1.02E-03
MIR99AHG:50	21	−8.35	1.50E-04
MIR99AHG:51	21	−8.35	1.47E-04
MIR99AHG:42	21	−8.35	1.54E-04
MIR99AHG:63	21	−8.37	1.50E-04
lnc-SPG7-2:3	16	−8.44	4.74E-04
lnc-ERAL1-1:1	17	−10.17	2.29E-03
lnc-SNAPC5-4:1	15	−10.33	3.18E-04
lnc-SNAPC5-4:2	15	−10.49	2.79E-04
SNHG1:1	11	−10.64	6.00E-04
lnc-SNURF-1:2	15	−10.67	1.22E-02
SNHG1:12	11	−10.79	1.50E-02
lnc-AFP-2:1	4	−11.47	1.18E-05
SNHG1:59	11	−13.06	7.36E-04
LRRC75A-AS1:49	17	−14.14	4.71E-04
lnc-RPL17-2:4	18	−14.76	2.66E-04
LINC00910:1	17	−14.92	1.14E-03
LINC00910:16	17	−15.01	1.14E-03
lnc-VSTM5-1:7	11	−17.05	3.53E-04
LRRC75A-AS1:41	17	−17.68	7.08E-04
lnc-MINA-3:4	3	−19.80	1.50E-04
lnc-SNURF-1:92	15	−20.18	6.06E-05
lnc-SNURF-1:95	15	−20.18	6.18E-05
lnc-TPTE-3:5	21	−20.61	1.72E-03
GAS5:43	1	−24.86	1.26E-05
lnc-ARHGEF6-4:1	X	−28.38	1.37E-06
LRRC75A-AS1:36	17	−31.63	4.59E-05
lnc-NEDD4L-1:6	18	−32.06	1.18E-05
lnc-CCNB1IP1-1:2	14	−34.31	2.78E-03
GAS5:41	1	−43.46	1.41E-03
lnc-TMEM132C-11:1	12	−67.46	6.92E-04
SNHG6:15	8	−103.09	7.10E-04
lnc-HSD17B10-3:1	X	−146.51	2.70E-04
lnc-AC022098.1-1:10	19	−576.95	3.27E-05
SNHG1:60	11	−693.84	4.85E-06
GAS5:7	1	−814.77	1.19E-05
GAS5:72	1	−814.77	1.19E-05
LINC01021:16	5	−1317.35	9.16E-06
lnc-MBNL2-3:1	13	−4157.50	9.10E-07

Differentially expressed lncRNAs in all five groups were listed in a Venn diagram (Fig. 6F), which showed, 109 lncRNAs were commonly expressed in all groups; whereas, 16 in cirrhosis, 3 in LGDN, 1 in HGDN, 39 in eHCC, and 3 in HCC were uniquely expressed (Fig. 6F). Several lncRNAs were commonly expressed between groups. Specifically, 19 in LGDN and eHCC; 10 in cirrhosis, LGDN, and eHCC; and 6 in LGDN, HGDN, eHCC, and HCC (Fig. 6F). We also organized a Circos Plot for comprehensive visualization of differentially expressed lncRNAs in the five groups with chromosome number and location (Fig. 6G). After plugging the data into Circos Plot, we found chromosomes 1, 2, 9, 13, 17, and X were the most enriched chromosomes in all five groups (Fig. 6G).

### Differential Expression of circRNA in Cirrhosis, LGDN, HGDN, eHCC, and HCC Tissue Samples

**Figure 7:**
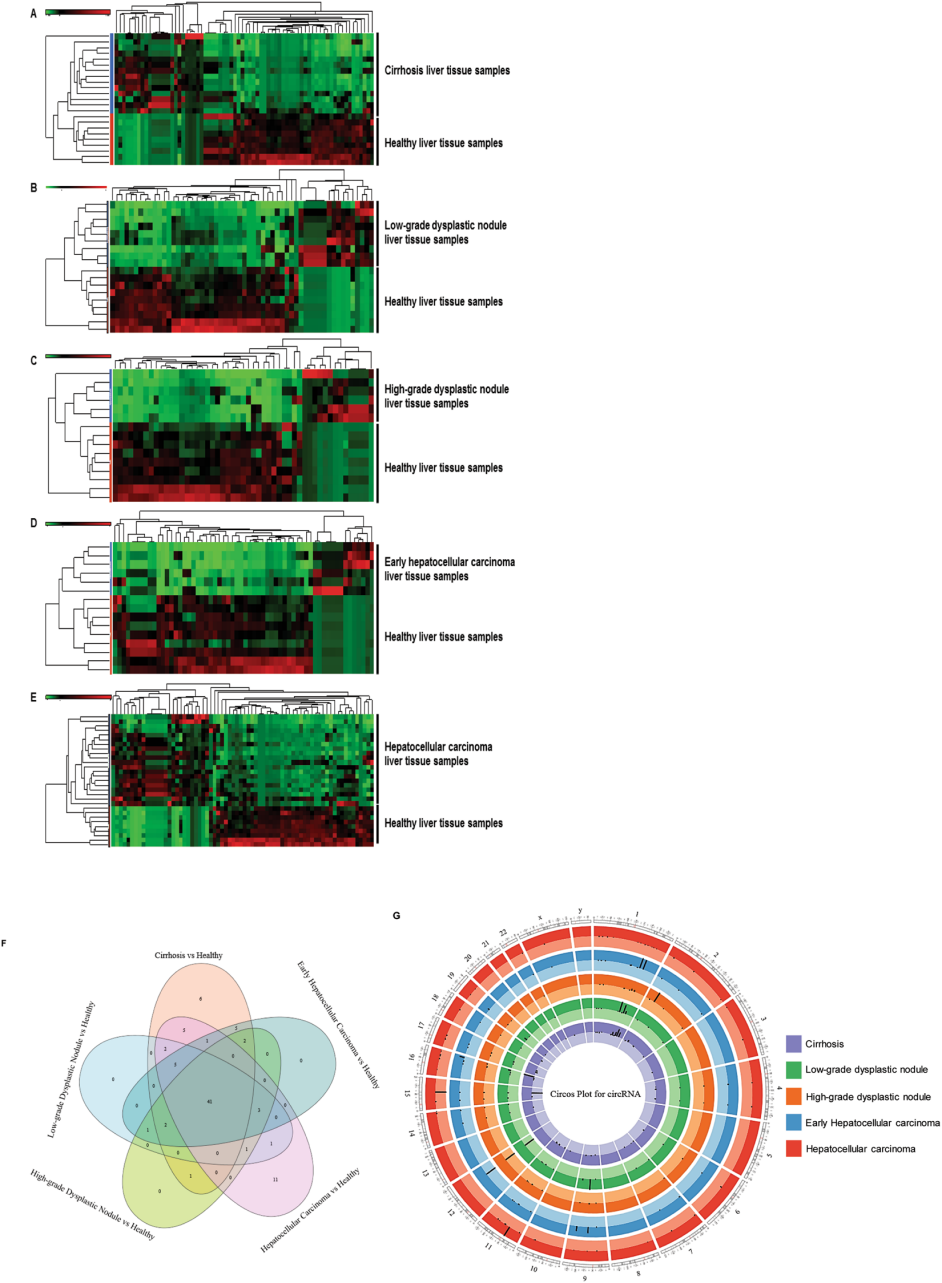
Differential expression of circular RNAs (circRNAs) in liver tissue samples: Differentially expressed circRNAs were quantified and a heatmap view was prepared (FDR < 0.05) for each disease stage (Cirrhosis, Low-grade dysplastic nodule, High-grade dysplastic nodule, Early stage Hepatocellular carcinoma and Advanced stage Hepatocellular carcinoma) with healthy control samples (**A**–**E**). All stages of liver disease enriched circRNAs were summarized by a Venn diagram, which identified 41 circRNAs commonly expressed in all stages and 11 circRNAs were specifically enriched in advanced hepatocellular carcinoma (**F**). (**G**) A Circos plot was prepared incorporating all differential expressions of circRNAs in cirrhosis, low-grade dysplastic nodule, high-grade dysplastic nodule, early hepatocellular carcinoma,and hepatocellular carcinoma tissue samples compared with healthy samples (FDR < 0.05). Chromosome and bands were listed in chromosomal positions of circRNAs affected expression in liver disease vs healthy samples. The Innermost ring is cirrhosis, followed by low-grade dysplastic nodule, high-grade dysplastic nodule, early hepatocellular carcinoma, and hepatocellular carcinoma with darker and lighter background colors representing upregulated and downregulated genes respectively.

We used circRNA annotation to identify differential expression in cirrhosis, LGDN, HGDN, eHCC, and HCC (Fig. 7A–E, Tables 15–19). We found 70 circRNAs in cirrhosis (24 upregulated and 46 downregulated; Fig. 7A, Table 15), 56 circRNAs in LGDN (16 upregulated and 40 downregulated; Fig. 7B, Table 16), 51 circRNAs in HGDN (14 upregulated and 37 downregulated; Fig. 7C, Table 17), 60 circRNAs in eHCC (14 upregulated and 46 downregulated; Fig. 7D, Table 18), and 70 circRNAs in HCC (26 upregulated and 44 downregulated; Fig. 7E, Table 19). The top five differentially upregulated circRNAs in cirrhosis (Table 15) were: circR-0040679 (2328-fold), circR-0015774 (1622-fold), circR-0016456 (1039-fold), circR-0015637 (810-fold), and circR-0014848 (591-fold); whereas, the top five downregulated were: circR-0035407 (258-fold), circR-0035409 (245-fold), circR-0092360 (39-fold), circR-0043980 (26-fold), and circR-0007956 (21-fold). The top five differentially upregulated circRNAs in LGDN (Table 16) were: circR-0087119 (455-fold), circR-0014848 (454-fold), circR-0015637 (369-fold), circR-0015774 (126-fold), and circR-0008347 (116-fold). circR-0006257 (48723-fold), circR-0035409 (2745-fold), circR-0034507 (791-fold), circR-0089763 (86-fold), and circR-0076872 (71-fold) were downregulated. The top five differentially upregulated circRNAs in HGDN (Table 17) were: circR-0054435 (2907-fold), circR-0015637 (454-fold), circR-0015774 (417-fold), circR-000007119 (265-fold), and circR-0040679 (211-fold); whereas the top five downregulated were: circR-0006257 (27596-fold), circR-0035409 (1234-fold), circR-0035407 (995-fold), circR-0076872 (44-fold), and circR-0092360 (28-fold). The top five differentially upregulated circRNAs in eHCC (Table 18) were: circR-0015637 (556-fold), circR-0015774 (516-fold), circR-0087119 (315-fold), circR-0040679 (258-fold), and circR-0087948 (254-fold); and circR-0006257 (19579-fold), circR-0035409 (810-fold), circR-0035407 (623-fold), circR-0076872 (47-fold), and circR-0092360 (29-fold) were downregulated. The top five differentially upregulated circRNAs in HCC (Table 19) were: circR-0021905 (800542-fold), circR-0016456 (1204-fold), circR-0015637 (608-fold), circR-0087119 (602-fold), and circR-0014848 (438-fold). The top five downregulated were: circR-0035409 (1219-fold), circR-0035407 (784-fold), circR-0076872 (98-fold), circR-0092360 (15-fold), and circR-0069970 (13-fold).

**Table 15:** Differentially expressed circRNAs in cirrhosis vs healthy patient’s tissue samples (FDR < 0.05).

Feature ID	Chromosome	Fold change	FDR
hsa_circ_0040679	16	2327.99	1.34E-08
hsa_circ_0015774	1	1621.70	5.47E-10
hsa_circ_0016456	1	1039.17	1.35E-07
hsa_circ_0015637	1	809.57	3.13E-11
hsa_circ_0014848	1	591.30	7.64E-07
hsa_circ_0054435	2	451.43	3.58E-08
hsa_circ_0087948	9	180.01	1.31E-14
hsa_circ_0087119	9	122.08	3.40E-04
hsa_circ_0019374	10	97.40	2.73E-06
hsa_circ_0012861	1	86.11	2.10E-06
hsa_circ_0008347	19	59.74	8.62E-05
hsa_circ_0069968	4	38.17	2.86E-06
hsa_circ_0069969	4	38.17	2.83E-06
hsa_circ_0079763	7	27.36	1.85E-02
hsa_circ_0006061	1	27.15	1.32E-08
hsa_circ_0069749	4	11.31	4.43E-04
hsa_circ_0009016	11	10.30	1.30E-06
hsa_circ_0043602	17	5.39	1.25E-02
hsa_circ_0043601	17	5.39	1.28E-02
hsa_circ_0043605	17	5.29	9.64E-03
hsa_circ_0043604	17	5.26	1.01E-02
hsa_circ_0043603	17	5.21	1.42E-02
hsa_circ_0034154	15	2.97	4.16E-02
hsa_circ_0004659	17	1.73	2.23E-02
hsa_circ_0034118	15	−2.15	1.70E-02
hsa_circ_0034110	15	−2.44	1.48E-02
hsa_circ_0055902	2	−2.53	2.75E-03
hsa_circ_0034138	15	−2.67	7.89E-03
hsa_circ_0022568	11	−2.96	4.03E-03
hsa_circ_0000146	1	−2.97	2.14E-02
hsa_circ_0031772	14	−3.14	5.91E-04
hsa_circ_0008733	1	−3.20	6.59E-03
hsa_circ_0002978	1	−3.21	6.16E-03
hsa_circ_0004784	1	−3.22	6.16E-03
hsa_circ_0006643	1	−3.22	5.89E-03
hsa_circ_0090906	X	−3.30	1.99E-02
hsa_circ_0000705	16	−3.55	2.16E-03
hsa_circ_0063778	22	−3.75	2.20E-03
hsa_circ_0067418	3	−4.22	3.72E-04
hsa_circ_0007319	7	−4.88	3.74E-06
hsa_circ_0006940	14	−5.24	7.79E-05
hsa_circ_0034121	15	−5.28	2.76E-03
hsa_circ_0092282	1	−5.34	7.09E-05
hsa_circ_0081371	7	−5.37	1.16E-05
hsa_circ_0069967	4	−5.57	2.23E-05
hsa_circ_0034117	15	−5.66	2.58E-03
hsa_circ_0010166	1	−6.21	2.36E-04
hsa_circ_0040412	16	−6.75	3.24E-06
hsa_circ_0001853	9	−6.84	1.21E-05
hsa_circ_0092300	2	−7.54	8.76E-05
hsa_circ_0092280	1	−7.97	2.15E-04
hsa_circ_0039972	16	−8.61	8.88E-06
hsa_circ_0004287	11	−9.77	1.35E-07
hsa_circ_0007636	1	−10.13	1.39E-03
hsa_circ_0008442	1	−10.29	9.87E-04
hsa_circ_0007702	2	−10.60	3.04E-06
hsa_circ_0052884	2	−10.78	1.62E-06
hsa_circ_0022547	11	−11.22	5.22E-05
hsa_circ_0092275	1	−11.82	2.50E-05
hsa_circ_0069970	4	−12.02	8.64E-07
hsa_circ_0092342	11	−14.00	2.17E-03
hsa_circ_0076872	6	−16.04	8.64E-07
hsa_circ_0024018	11	−17.54	1.58E-03
hsa_circ_0024019	11	−17.91	5.58E-04
hsa_circ_0071320	4	−19.54	7.31E-07
hsa_circ_0007956	18	−20.82	1.47E-03
hsa_circ_0043980	17	−26.23	3.38E-06
hsa_circ_0092360	17	−38.68	7.66E-05
hsa_circ_0035409	15	−248.93	8.64E-07
hsa_circ_0035407	15	−258.02	6.97E-07

**Table 16:** Differentially expressed circRNAs in low-grade dysplastic nodule vs healthy patient’s tissue samples (FDR < 0.05).

Feature ID	Chromosome	Fold change	FDR
hsa_circ_0087119	9	454.61	2.78E-05
hsa_circ_0014848	1	454.34	1.95E-05
hsa_circ_0015637	1	368.71	7.33E-11
hsa_circ_0015774	1	126.00	1.72E-08
hsa_circ_0008347	19	116.32	2.00E-09
hsa_circ_0040679	16	111.54	1.93E-09
hsa_circ_0087948	9	60.83	7.33E-11
hsa_circ_0043602	17	19.26	6.33E-04
hsa_circ_0043601	17	19.22	6.33E-04
hsa_circ_0043605	17	18.79	5.24E-04
hsa_circ_0043603	17	18.67	5.99E-04
hsa_circ_0043604	17	18.63	5.37E-04
hsa_circ_0006061	1	10.72	2.54E-06
hsa_circ_0009016	11	5.35	3.47E-04
hsa_circ_0051491	19	4.94	2.45E-03
hsa_circ_0064383	3	2.35	3.79E-02
hsa_circ_0063778	22	−1.73	4.65E-02
hsa_circ_0006940	14	−1.82	4.56E-02
hsa_circ_0031772	14	−1.86	1.94E-02
hsa_circ_0055902	2	−1.97	1.26E-02
hsa_circ_0000705	16	−2.14	4.01E-03
hsa_circ_0004489	14	−2.29	1.54E-02
hsa_circ_0001853	9	−2.30	1.05E-02
hsa_circ_0010166	1	−2.45	3.93E-02
hsa_circ_0034110	15	−2.53	2.13E-02
hsa_circ_0002571	14	−3.01	2.14E-03
hsa_circ_0052884	2	−3.15	4.52E-03
hsa_circ_0040412	16	−3.37	1.25E-03
hsa_circ_0022568	11	−3.37	8.86E-04
hsa_circ_0034117	15	−3.70	3.26E-02
hsa_circ_0034138	15	−3.74	1.60E-03
hsa_circ_0022547	11	−3.85	1.41E-02
hsa_circ_0071320	4	−4.26	1.78E-03
hsa_circ_0092342	11	−4.49	1.37E-03
hsa_circ_0092282	1	−4.70	4.02E-05
hsa_circ_0069967	4	−4.73	6.48E-05
hsa_circ_0092300	2	−4.87	1.46E-04
hsa_circ_0081371	7	−4.92	1.09E-04
hsa_circ_0034118	15	−5.09	1.31E-02
hsa_circ_0092275	1	−5.35	1.93E-03
hsa_circ_0069970	4	−5.44	1.47E-04
hsa_circ_0092280	1	−5.76	4.92E-04
hsa_circ_0034121	15	−6.03	1.35E-02
hsa_circ_0004287	11	−6.07	8.33E-05
hsa_circ_0007319	7	−8.02	1.86E-08
hsa_circ_0043980	17	−8.35	1.31E-03
hsa_circ_0024018	11	−8.97	3.79E-05
hsa_circ_0024019	11	−9.04	3.49E-05
hsa_circ_0039972	16	−9.50	1.79E-05
hsa_circ_0007956	18	−10.74	1.52E-05
hsa_circ_0092360	17	−25.62	1.66E-07
hsa_circ_0076872	6	−71.14	2.26E-10
hsa_circ_0089763	M	−86.14	9.20E-06
hsa_circ_0035407	15	−790.56	6.21E-07
hsa_circ_0035409	15	−2745.28	5.53E-08
hsa_circ_0006257	12	−48722.51	2.34E-09

**Table 17:** Differentially expressed circRNAs in high-grade dysplastic nodule vs healthy patient’s tissue samples (FDR < 0.05).

Feature ID	Chromosome	Fold change	FDR
hsa_circ_0054435	2	2907.43	3.25E-03
hsa_circ_0015637	1	454.29	1.93E-07
hsa_circ_0015774	1	416.69	1.82E-05
hsa_circ_0087119	9	265.25	7.85E-04
hsa_circ_0040679	16	211.05	1.61E-06
hsa_circ_0087948	9	90.25	4.85E-07
hsa_circ_0008347	19	83.53	4.47E-06
hsa_circ_0006061	1	13.47	1.72E-03
hsa_circ_0009016	11	7.07	4.46E-03
hsa_circ_0043602	17	6.37	3.17E-02
hsa_circ_0043601	17	6.36	3.58E-02
hsa_circ_0043605	17	6.16	2.94E-02
hsa_circ_0043603	17	6.16	3.60E-02
hsa_circ_0051491	19	3.45	1.84E-02
hsa_circ_0063778	22	−1.93	4.40E-02
hsa_circ_0089763	M	−1.98	2.43E-02
hsa_circ_0006940	14	−2.35	2.96E-02
hsa_circ_0004489	14	−2.45	1.84E-02
hsa_circ_0031772	14	−2.48	1.08E-02
hsa_circ_0055902	2	−3.03	1.46E-04
hsa_circ_0022568	11	−3.29	1.13E-02
hsa_circ_0071320	4	−3.43	2.60E-03
hsa_circ_0002571	14	−3.67	2.20E-03
hsa_circ_0040412	16	−4.35	7.55E-05
hsa_circ_0052884	2	−4.42	8.04E-03
hsa_circ_0008442	1	−4.71	4.12E-02
hsa_circ_0007636	1	−4.77	3.48E-02
hsa_circ_0069967	4	−5.12	1.01E-04
hsa_circ_0022547	11	−5.64	1.08E-02
hsa_circ_0069970	4	−5.81	6.97E-05
hsa_circ_0034138	15	−5.91	4.11E-04
hsa_circ_0092300	2	−6.18	4.22E-04
hsa_circ_0092342	11	−6.77	4.34E-02
hsa_circ_0001853	9	−7.36	5.60E-05
hsa_circ_0092280	1	−7.72	2.17E-03
hsa_circ_0004287	11	−8.52	4.90E-05
hsa_circ_0092282	1	−8.66	1.84E-05
hsa_circ_0092275	1	−9.62	4.47E-03
hsa_circ_0007319	7	−9.86	4.85E-07
hsa_circ_0039972	16	−10.98	3.05E-05
hsa_circ_0007956	18	−12.27	3.78E-04
hsa_circ_0034121	15	−12.37	2.80E-03
hsa_circ_0024019	11	−12.37	1.24E-04
hsa_circ_0024018	11	−12.67	1.11E-04
hsa_circ_0034118	15	−14.24	2.70E-04
hsa_circ_0043980	17	−16.26	1.07E-03
hsa_circ_0092360	17	−27.80	9.02E-03
hsa_circ_0076872	6	−43.61	4.85E-07
hsa_circ_0035407	15	−994.94	7.76E-11
hsa_circ_0035409	15	−1234.90	1.96E-07
hsa_circ_0006257	12	−27596.01	4.85E-07

**Table 18:** Differentially expressed circRNAs in early hepatocellular carcinoma vs healthy patient’s tissue samples (FDR < 0.05).

Feature ID	Chromosome	Fold change	FDR
hsa_circ_0015637	1	556.31	8.94E-09
hsa_circ_0015774	1	515.70	2.74E-07
hsa_circ_0087119	9	314.69	2.71E-03
hsa_circ_0040679	16	258.13	4.88E-08
hsa_circ_0087948	9	253.91	3.36E-07
hsa_circ_0008347	19	88.59	2.08E-05
hsa_circ_0069969	4	32.75	4.85E-02
hsa_circ_0006061	1	27.89	1.87E-04
hsa_circ_0043602	17	13.55	5.26E-03
hsa_circ_0043601	17	13.49	5.26E-03
hsa_circ_0043605	17	13.24	4.89E-03
hsa_circ_0043604	17	13.09	4.94E-03
hsa_circ_0043603	17	13.05	5.89E-03
hsa_circ_0009016	11	7.27	1.64E-03
hsa_circ_0031772	14	−2.24	1.14E-02
hsa_circ_0063778	22	−2.37	9.10E-03
hsa_circ_0055902	2	−2.48	1.75E-02
hsa_circ_0006940	14	−2.49	1.38E-02
hsa_circ_0004489	14	−2.60	1.38E-02
hsa_circ_0089763	M	−2.96	1.21E-03
hsa_circ_0000705	16	−2.97	4.25E-03
hsa_circ_0002978	1	−3.04	2.56E-02
hsa_circ_0008733	1	−3.04	2.72E-02
hsa_circ_0006643	1	−3.04	2.39E-02
hsa_circ_0004784	1	−3.05	2.52E-02
hsa_circ_0081371	7	−3.17	5.16E-03
hsa_circ_0052884	2	−3.39	1.91E-03
hsa_circ_0002571	14	−3.50	4.16E-03
hsa_circ_0001853	9	−3.68	2.89E-03
hsa_circ_0090906	X	−3.71	1.12E-02
hsa_circ_0034138	15	−3.86	2.86E-03
hsa_circ_0034118	15	−3.94	6.64E-03
hsa_circ_0022568	11	−4.34	5.88E-03
hsa_circ_0004287	11	−4.83	1.05E-03
hsa_circ_0010166	1	−4.92	3.53E-03
hsa_circ_0092275	1	−4.94	6.76E-03
hsa_circ_0040412	16	−5.00	2.97E-04
hsa_circ_0008442	1	−5.31	2.15E-02
hsa_circ_0007636	1	−5.38	2.08E-02
hsa_circ_0092282	1	−5.55	3.43E-04
hsa_circ_0069967	4	−6.01	7.74E-05
hsa_circ_0092342	11	−6.18	3.14E-02
hsa_circ_0007319	7	−6.26	2.30E-06
hsa_circ_0092300	2	−6.59	2.29E-03
hsa_circ_0034117	15	−6.66	3.19E-03
hsa_circ_0022547	11	−6.69	6.43E-03
hsa_circ_0092280	1	−6.87	3.00E-03
hsa_circ_0071320	4	−8.06	4.03E-04
hsa_circ_0034121	15	−8.26	4.71E-03
hsa_circ_0069970	4	−9.34	4.64E-05
hsa_circ_0039972	16	−10.95	9.20E-05
hsa_circ_0024018	11	−11.52	4.20E-04
hsa_circ_0024019	11	−11.60	4.03E-04
hsa_circ_0007956	18	−13.18	3.43E-04
hsa_circ_0043980	17	−15.09	1.13E-03
hsa_circ_0092360	17	−29.19	7.77E-05
hsa_circ_0076872	6	−46.60	1.73E-07
hsa_circ_0035407	15	−622.97	4.30E-05
hsa_circ_0035409	15	−810.36	3.04E-05
hsa_circ_0006257	12	−19578.54	1.03E-06

**Table 19:** Differentially expressed circRNAs in hepatocellular carcinoma vs healthy patient’s tissue samples (FDR < 0.05).

Feature ID	Chromosome	Fold change	FDR
hsa_circ_0021905	11	800542.00	5.29E-31
hsa_circ_0016456	1	1204.47	4.52E-10
hsa_circ_0015637	1	607.83	1.37E-18
hsa_circ_0087119	9	602.14	3.06E-09
hsa_circ_0014848	1	437.51	1.49E-09
hsa_circ_0015774	1	308.14	4.00E-11
hsa_circ_0040679	16	228.28	5.92E-12
hsa_circ_0087948	9	125.30	4.13E-16
hsa_circ_0008347	19	114.16	1.65E-14
hsa_circ_0019374	10	61.21	1.36E-04
hsa_circ_0012861	1	24.27	6.41E-11
hsa_circ_0043602	17	21.84	1.24E-05
hsa_circ_0043601	17	21.55	1.23E-05
hsa_circ_0043605	17	21.26	1.63E-06
hsa_circ_0043604	17	21.13	1.68E-06
hsa_circ_0043603	17	21.00	1.37E-05
hsa_circ_0006061	1	13.52	1.62E-09
hsa_circ_0002189	8	7.83	7.01E-07
hsa_circ_0009060	16	7.02	1.68E-05
hsa_circ_0034021	14	5.93	1.57E-02
hsa_circ_0005554	14	5.15	1.94E-02
hsa_circ_0009016	11	4.44	2.22E-07
hsa_circ_0051491	19	3.74	8.30E-05
hsa_circ_0064383	3	3.61	1.36E-04
hsa_circ_0016169	1	2.87	3.30E-02
hsa_circ_0004659	17	1.56	2.21E-02
hsa_circ_0089763	M	−1.12	1.01E-03
hsa_circ_0071319	4	−1.54	2.20E-02
hsa_circ_0010166	1	−1.91	3.06E-02
hsa_circ_0019218	10	−2.20	6.32E-04
hsa_circ_0004489	14	−2.21	3.26E-03
hsa_circ_0034110	15	−2.23	1.15E-02
hsa_circ_0000705	16	−2.38	3.34E-05
hsa_circ_0055902	2	−2.39	8.97E-05
hsa_circ_0001853	9	−2.41	1.96E-03
hsa_circ_0090906	X	−2.47	1.19E-02
hsa_circ_0063778	22	−2.50	6.21E-03
hsa_circ_0022568	11	−2.52	1.42E-03
hsa_circ_0034138	15	−2.54	6.47E-03
hsa_circ_0092280	1	−2.69	1.19E-02
hsa_circ_0002571	14	−2.76	1.58E-03
hsa_circ_0034121	15	−2.97	8.08E-03
hsa_circ_0034117	15	−2.98	1.75E-02
hsa_circ_0092342	11	−2.99	1.47E-03
hsa_circ_0022547	11	−3.30	5.06E-03
hsa_circ_0067418	3	−3.70	3.66E-05
hsa_circ_0040412	16	−3.82	4.65E-05
hsa_circ_0092300	2	−3.82	1.70E-05
hsa_circ_0003812	11	−3.90	1.13E-03
hsa_circ_0004287	11	−4.24	1.59E-07
hsa_circ_0081371	7	−4.36	6.84E-07
hsa_circ_0092275	1	−4.65	2.76E-04
hsa_circ_0052884	2	−4.89	2.41E-06
hsa_circ_0092282	1	−5.04	5.12E-09
hsa_circ_0052882	2	−5.37	1.52E-07
hsa_circ_0034118	15	−6.34	5.39E-04
hsa_circ_0071323	4	−6.56	1.50E-05
hsa_circ_0007319	7	−6.85	1.94E-11
hsa_circ_0007956	18	−7.25	3.56E-06
hsa_circ_0024019	11	−7.44	2.24E-06
hsa_circ_0024018	11	−7.58	1.65E-06
hsa_circ_0071320	4	−7.99	2.41E-06
hsa_circ_0039972	16	−8.42	4.52E-10
hsa_circ_0069967	4	−11.02	4.61E-11
hsa_circ_0043980	17	−11.70	1.15E-05
hsa_circ_0069970	4	−13.17	1.15E-09
hsa_circ_0092360	17	−15.18	2.95E-08
hsa_circ_0076872	6	−97.52	8.42E-19
hsa_circ_0035407	15	−783.68	3.39E-11
hsa_circ_0035409	15	−1219.10	3.39E-11

A Venn diagram (Fig. 7F) showed, 41 circRNAs commonly expressed in all groups whereas, six in cirrhosis; none in LGDN, HGDN, oreHCC; and 11 in HCC were uniquely expressed (Fig. 7F). There were several circRs commonly expressed between groups: five in cirrhosis and HCC; five in cirrhosis, LGDN, eHCC, and HCC; and three in LGDN, HGDN, eHCC, and HCC (Fig. 7F). We also prepared a Circos Plot for comprehensive visualization of differentially expressed circRNAs (Fig. 7G) which found chromosomes 1, 2, 5, 6, and 22 to be the most enriched across groups (Fig. 7G).

### Differential Expression of sno/mt-RNA in Cirrhosis, LGDN, HGDN, eHCC, and HCC Tissue Samples

**Figure 8:**
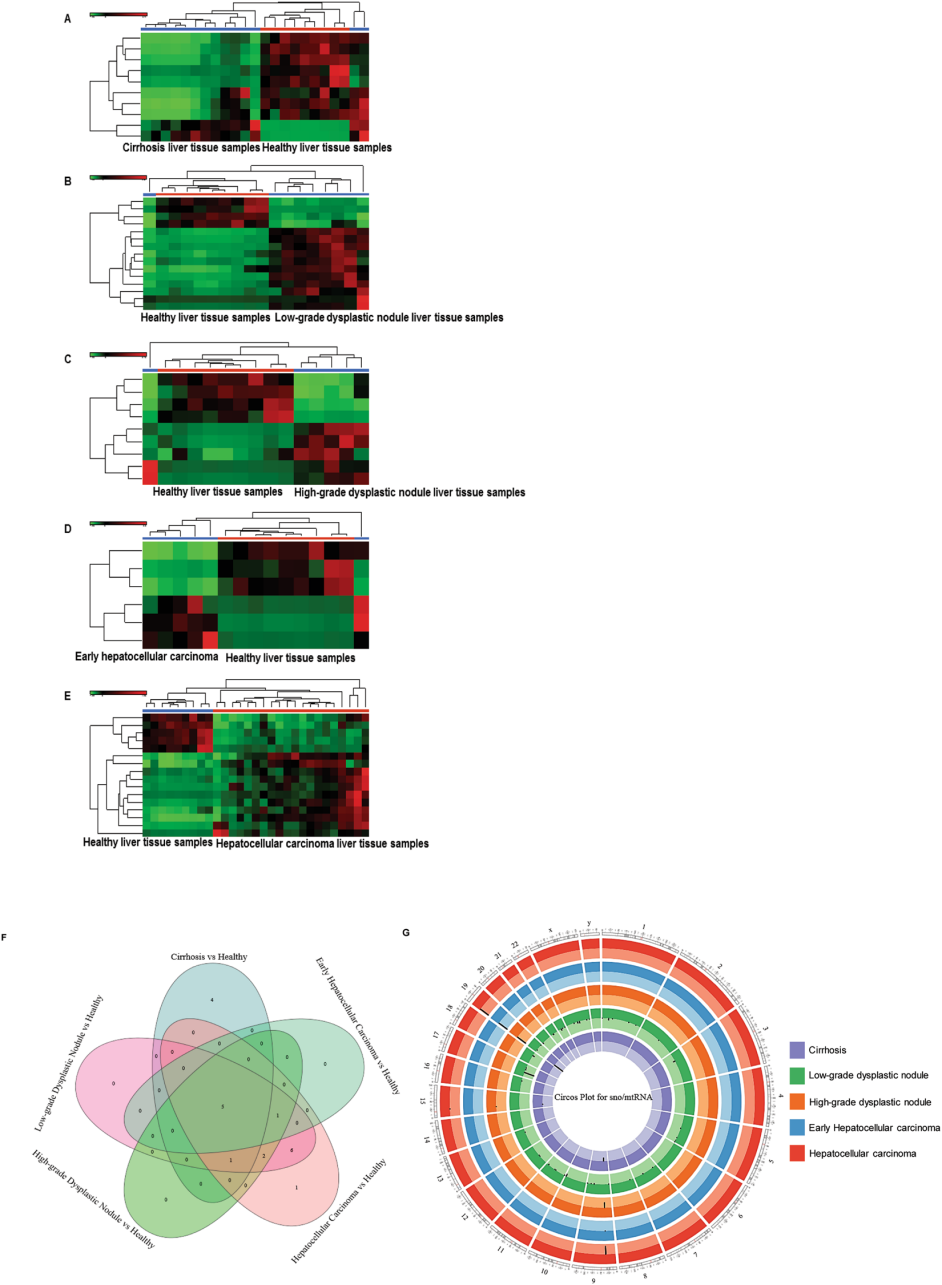
Differential expression of snoRNAs and mitochondrial RNAs (mt-RNAs) liver tissue samples: Differential expression of snoRNAs and mt-RNAs were quantified and a heatmap view was prepared (FDR < 0.05) for each disease stage (Cirrhosis, Low-grade dysplastic nodule, High-grade dysplastic nodule, Early stage Hepatocellular carcinoma and Advanced stage of Hepatocellular carcinoma) with healthy control samples (**A**–**E**). All stages of liver disease enriched sno/mt-RNAs were summarized by a Venn diagram, which identified 5 small RNAs commonly expressed in all stages and 1 snoRNA was specifically enriched in advanced hepatocellular carcinoma (**F**). (**G**) A Circos plot was prepared incorporating all differential expressions of sno/mt-RNAs in cirrhosis, LGDN, HGDN, eHCC and HCC tissue samples compared with healthy samples (FDR < 0.05). Chromosome and bands were listed in chromosomal positions of sno/mt-RNAs expression in liver disease vs healthy samples. The Innermost ring is cirrhosis, then low-grade dysplastic nodule, high-grade dysplastic nodule, early hepatocellular carcinoma and hepatocellular carcinoma with darker and lighter background colors representing upregulated and downregulated genes respectively.

Small nuclear RNAs form a class of RNA molecules that localize within the nucleus of eukaryotic cells^28^. Their primary function is pre-mRNA processing, for which they are always associated with a set of specific proteins. These complexes are referred to as small nuclear ribonucleoproteins (snRNP). The small nucleolar RNAs (snoRNAs) are another subclass of snRNA that localize in the nucleolus and are associated with the maturation of RNA molecules through chemical modifications targeting mainly rRNAs, tRNAs, and snRNAs^28^. We remapped aligned reads to GenCode v26 database, which contains most of the curated small RNAs to identify differential expression in cirrhosis, LGDN, HGDN, eHCC, and HCC (Fig. 8A–E, Tables 20–24). We found 10 sno/mt-RNAs in cirrhosis (Fig. 8A, Table 20), 15 sno/mt-RNAs in LGDN (Fig. 8B and Table 21), nine sno/mt-RNAs in HGDN (Fig 8C and Table 22), six sno/mt-RNAs in eHCC (Fig. 8D and Table 23), and 16 sno/mt-RNAs in HCC (Fig. 8E and Table 24). Most of the mitochondrial RNAs were upregulated in HCC and all four snoRNAs were downregulated. All five groups had downregulated snoRD121B, whereas snoRD121A was downregulated only in HCC samples.

**Table 20:** Differentially expressed sno/mt RNAs in cirrhosis vs healthy patient’s tissue samples (FDR < 0.05).

Gene name	Gene type	Chromosome	Fold change	FDR
MT-TS1	Mt_tRNA	M	45.05	4.11E-10
MT-TP	Mt_tRNA	M	38.27	3.33E-09
MT-TH	Mt_tRNA	M	−1.56	3.48E-02
MT-TI	Mt_tRNA	M	−2.05	4.25E-02
MT-TL1	Mt_tRNA	M	−2.19	9.59E-03
MT-TK	Mt_tRNA	M	−4.03	9.31E-04
MT-TS2	Mt_tRNA	M	−6.09	5.28E-05
SNORD115-31	snoRNA	15	−9.48	3.45E-03
SNORD37	snoRNA	19	−13.81	8.28E-06
SNORD121B	snoRNA	9	−15.21	6.44E-07

**Table 21:** Differentially expressed sno/mt RNAs in low-grade dysplastic nodule vs healthy patient’s tissue samples (FDR < 0.05).

Gene name	Gene type	Chromosome	Fold change	FDR
MT-TP	Mt_tRNA	M	27.11	5.90E-07
MT-TS1	Mt_tRNA	M	25.43	5.13E-06
MT-TT	Mt_tRNA	M	12.34	7.07E-05
MT-TQ	Mt_tRNA	M	11.95	1.47E-04
MT-TN	Mt_tRNA	M	11.56	8.95E-04
MT-TL2	Mt_tRNA	M	10.88	7.37E-05
MT-TC	Mt_tRNA	M	7.62	3.19E-03
MT-TG	Mt_tRNA	M	5.24	1.04E-02
MT-TE	Mt_tRNA	M	4.52	1.84E-03
MT-TV	Mt_tRNA	M	4.19	2.14E-02
MT-TM	Mt_tRNA	M	4.18	1.23E-02
MT-TS2	Mt_tRNA	M	−3.29	1.60E-03
SNORD115-31	snoRNA	15	−4.42	7.03E-04
SNORD121B	snoRNA	9	−8.06	1.65E-05
SNORD37	snoRNA	19	−15.97	1.10E-07

**Table 22:** Differentially expressed sno/mt RNAs in high-grade dysplastic nodule vs healthy patient’s tissue samples (FDR < 0.05).

Gene name	Gene type	Chromosome	Fold change	FDR
MT-TP	Mt_tRNA	M	21.76	1.42E-05
MT-TS1	Mt_tRNA	M	19.48	3.85E-05
MT-TT	Mt_tRNA	M	10.44	6.55E-05
MT-TQ	Mt_tRNA	M	8.22	2.66E-03
MT-TC	Mt_tRNA	M	2.58	2.29E-02
MT-TS2	Mt_tRNA	M	−4.32	1.27E-03
SNORD115-31	snoRNA	15	−4.75	4.10E-02
SNORD121B	snoRNA	9	−11.02	1.33E-05
SNORD37	snoRNA	19	−17.68	1.44E-05

**Table 23:** Differentially expressed sno/mt RNAs in early hepatocellular carcinoma vs healthy patient’s tissue samples (FDR < 0.05).

Gene name	Gene type	Chromosome	Fold change	FDR
MT-TP	Mt_tRNA	M	31.48	6.80E-07
MT-TS1	Mt_tRNA	M	26.18	2.11E-06
MT-TT	Mt_tRNA	M	13.18	8.81E-03
MT-TS2	Mt_tRNA	M	−3.77	2.52E-03
SNORD121B	snoRNA	9	−10.05	4.43E-04
SNORD37	snoRNA	19	−11.78	6.27E-05

**Table 24:** Differentially expressed sno/mt RNAs in hepatocellular carcinoma vs healthy patient’s tissue samples (FDR < 0.05).

Gene name	Gene type	Chromosome	Fold change	FDR
MT-TP	Mt_tRNA	M	20.68	6.96E-09
MT-TS1	Mt_tRNA	M	12.82	1.34E-06
MT-TT	Mt_tRNA	M	12.23	3.65E-05
MT-TL2	Mt_tRNA	M	10.90	8.32E-05
MT-TQ	Mt_tRNA	M	8.34	1.11E-07
MT-TN	Mt_tRNA	M	7.23	2.23E-05
MT-TV	Mt_tRNA	M	5.11	1.74E-02
MT-TM	Mt_tRNA	M	4.95	1.00E-03
MT-TG	Mt_tRNA	M	4.23	2.25E-02
MT-TC	Mt_tRNA	M	4.21	1.76E-06
MT-TE	Mt_tRNA	M	3.05	3.94E-04
MT-TS2	Mt_tRNA	M	−3.23	1.50E-04
SNORD115-31	snoRNA	15	−4.34	5.04E-04
SNORD121B	snoRNA	9	−6.87	1.24E-07
SNORD121A	snoRNA	9	−7.45	1.11E-07
SNORD37	snoRNA	19	−8.32	1.12E-07

Differentially expressed data for all five groups is shown in a Venn diagram (Fig. 8F), that demonstrated five sno/mt-RNAs commonly expressed in all groups whereas, four in cirrhosis; none in LGDN, HGDN, or eHCC; and one in HCC uniquely expressed (Fig. 8F). There were several sno/mt-RNAs commonly expressed between groups: six in LGDN and HCC; and one in LGDN, HGDN, and HCC (Fig. 8F). We also prepared a Circos Plot for comprehensive visualization of differentially expressed sno/mt-RNAs in the five groups (Fig. 8G). Chromosomes 9, 17, and 19 were the most enriched (Fig. 8F).

## Discussion

We have identified various non-coding RNAs in liver disease samples, which can be used for validation and development of novel therapeutics for HCC. miR-101 regulates proliferation, migration, and invasion in various cancers^29–32^; suggesting importance in the ordered transformation from normal to malignant phenotype. This was also suggested in our study as miR-101 was continually overexpressed in all disease stages when compared to normal liver tissue. miR-22 is considered to have tumor suppressor activity; however, in our study, it showed remarkable overexpression in HCC^33^. miR-23a has been reported to downregulate expression of interferon regulatory factor-1 in HCC^34^. Accordingly, it was overexpressed in the eHCC samples we analyzed. miR-7704 was found to be highly overexpressed in HCC when compared to cirrhosis (~400-fold change).

Current therapies and targeted strategies are well documented in the literature; however, more research is needed to identify crucial players in early disease development and treatment targets. Deregulation of various pathways such as p53, RAS/MAPK, PI3K/AKT/mTOR, WNT, MET, MYC, and TGF-beta are involved in oncogenesis^35^. Interestingly, miR-122 affects all of these pathways while also targeting CUTL1 transcriptional repression^35,36^, leading to apoptosis and cell cycle arrest. Accordingly, miR-122 is downregulated in more than 70% of cancers, suggesting a crucial role in oncologic transformation. miR-122 appears to act as a tumor suppressor and its downregulation in our study may be pertinent to an ordered progression from normal liver to HCC phenotype. In our analysis, miR-122 was downregulated across all disease stages in both sequenced data and clinical samples (Fig. 1H).

The Lethal-7 (Let-7) family of miRNAs is present in multiple copies in the genome and highly conserved^37^. These miRNAs are known to act as tumor suppressors and prevent angiogenesis. The Let-7 family has 10 members in the human genome (Let-7a, let-7b, let-7c, let-7d, let-7e, let-7f, let-7g, let-7i, miR-98, and miR-202^38^), which are involved in gene regulation and cell adhesion. We found, Let-7f microRNAs were strikingly downregulated in HGDN and eHCC (1 million-fold change) and Let-7g in HCC (358-fold change) with similar reports published in gastric^39,40^, prostate^41^, colon^42,43^, small cell lung^44^, thyroid^45^, breast^46,47^, and hepatocellular cancer^48,49^. miR-221, was found to be upregulated in HCC via in silico analysis and has multiple known pathway targets, such as p27Kip1, p53, BMF, PI3K, PTEN, and mTOR^50^. Vascular endothelial growth factor (VEGF) plays a major role in tumor development, and is partly regulated by miR-16. Our data correlates with recent studies that have shown miR-16 upregulation in HCC. GEMOX (Gemcitabine and oxaliplatin) is one of the chemotherapeutic options in HCC treatment, which specifically targets VEGF and miR-16^51^. Other available chemotherapeutic agents such as sunitibib, linifanib, brivanib, tivantibib, and everolimus target other kinases. Specifically, everolimus targets mTOR signaling and mTOR regulated miRNAs such as miR-99a-3p (upregulated), miR-99a-5p (downregulated), miR-221 (upregulated), and miR-100 (downregulated).

Most circular RNA functions are not clear. Until now, the only clear evidence is that they can serve as miRNA “sponges”^52^. In our study, we found altered expression of many circular RNAs, however additional validation is needed prior to assigning functionality.

Overall, 37 dysregulated sncRNAs were shared between all five phenotypic groups. miR-101, miR-22, and circR-0015774 were the top upregulated sncRNAs, whereas miR-122, piR-952, and circR-0035409 were the most frequently downregulated. 30 piRNAs, 109 lncRNAs, 41 circRNAs, and five sno/mt-RNAs were downregulated in all groups. circR-0015774, circR-0035409, MT-TS1, and MT-TP were in the top five upregulated RNAs in all groups; whereas, sno115-31 and snoRD37 were in the top five downregulated. Specific RNAs were also dysregulated in a single group. In cirrhosis, 16 miRNAs were dysregulated whereas four were dysregulated in LGDN (let-7d, miR-141, miR-181b, and miR-3120), none in HGDN, miR-150 in eHCC, and 29 in HCC. miR200b was commonly downregulated in HGDN, eHCC, and HCC (<3.5-fold). Six piRNAs were affected only in cirrhosis, seven in HGDN, and 52 in HCC. 16 lncRNAs were affected in cirrhosis, three in LGDN (lnc-C17orf51-5:1, lnc-KDM4C-18:1, and SNHG1:57), one in HGDN (lnc-REG3G-6:1), 39 in eHCC, and three in HCC (LINC01021:16, lnc-MBNL2-3:1, and SNHG1:60). Six circRNAs in cirrhosis and 11 in HCC were specifically dysregulated. Four sno/mt-RNAs were dysregulated in cirrhosis only and one in HCC (snoRD121A).

Our results summarize that multiple differentially expressed sncRNAs were identifided in concordance with disease progression, which may provide a basis for future study and clues to understanding HCC pathogenesis. Although we have a substantial sequencing dataset, we were limited in miRNA validation by a small tissue sample size. Additionally, limited data on disease etiology prevented further analysis. Nevertheless, we believe that our study uncovers the potential of data mining for sncRNA identification with regards to HCC. It is also appreciated that further research is needed to address the functional validation of sncRNA signatures and their relevance.

In summary, miR-101, miR-22, miR-122, circR-0015774, circR-0035409, MT-TS1, MT-TP, sno115-31, and snoRD37 may serve as biomarkers for liver pathogenesis, since they were differentially expressed. Each liver phenotype demonstrated a unique molecular signature. In cirrhosis, there were 16 dysregulated miRNAs, six piRNAs, 16 lncRNAs, and six circRNAs. Four miRNAs and three lncRNAs were differentially expressed in LGDN; whereas, seven piRNAs and one lncRNA were dysregulated in HGDN. The molecular signature of eHCC was altered by one miRNA and 39 lncRNAs, while HCC demonstrated changes in 29 miRNAs, 52 piRNAs, three lncRNAs, 11 circRNAs, and one sno/mt-RNA. The sncRNAs most strongly associated with cirrhosis were mir-192 (32-fold), miR 320b (14-fold), and circ-0079763 (27-fold). LGDN was most defined by changes in miR-141(626-fold) and piR-25782.5 (309-fold), while piR-25782.1 (181-fold) was expressed in HGDN. miR-150 (4-fold) was most strongly associated with eHCC, whereas, miR-142 (1-million fold), miR23a (124-fold), miR 130b (65-fold), piR-23670 (2335-fold), piR-24684 (2072-fold), circR-0021905, and snoRD121A were specifically altered in HCC. Further studies are needed to validate sncRNA functionality in liver disease prior to biomarker development.

## Electronic supplementary material

Supplementary Information
