# Supplementary material for: Non-coding RNAs in Various Stages of Liver Disease Leading to Hepatocellular Carcinoma: Differential Expression of miRNAs, piRNAs, lncRNAs, circRNAs, and sno/mt-RNAs
Source: Sci Rep. 2018 May 22;8:7967. doi: 10.1038/s41598-018-26360-1 (PMC5964116; doi:10.1038/s41598-018-26360-1)
Supplement: Supplementary file 1 — Supplementary Information [file 41598_2018_26360_MOESM1_ESM.docx]

**Non-coding RNAs in Various Stages of Liver Disease Leading to Hepatocellular Carcinoma: Differential Expression of miRNAs, piRNAs, lncRNAs, circRNAs, and sno/mt-RNAs**

**Srinivas V. Koduru^1^*, Ashley N. Leberfinger^1^, Yuka I. Kawasawa^2^, Milind Mahajan^3^, Niraj J. Gusani^4^, Arun J. Sanyal^5^, Dino J. Ravnic^1*^**

**^1^**Division of Plastic Surgery, Department of Surgery, Pennsylvania State University College of Medicine, 500 University Drive, Hershey, PA 17033

**^2^**Department of Pharmacology, Department of Biochemistry & Molecular Biology, and Institute for Personalized Medicine, Pennsylvania State University College of Medicine, 500 University Drive, Hershey, PA 17033

**^3^**Genomics Facility, Department of Genetics and Genomics Sciences, Icahn School of Medicine, Mount Sinai, 1425 Madison Ave, New York, NY 10029

**^4^**Program for Liver, Pancreas, & Foregut Tumors, Department of Surgery, Pennsylvania State University College of Medicine, 500 University Drive, Hershey, PA 17033

**^5^**Division of Gastroenterology, Hepatology and Nutrition, Department of Internal Medicine, Virginia Commonwealth University, 1201 E Marshall St, Richmond, VA 23298

**Table 1: Differentially expressed lncRNAs in low-grade dysplastic nodule vs healthy patient’s tissue samples (FDR <0.05)**

| **lncRNA ID** | **Chromosome** | **Fold change** | **FDR** |
| --- | --- | --- | --- |
| lnc-CRK-3:2 | 17 | 1830.14 | 7.66E-06 |
| lnc-GCNT1-4:6 | 9 | 355.48 | 1.91E-13 |
| lnc-GCNT1-4:5 | 9 | 344.82 | 1.10E-12 |
| lnc-GCNT1-4:2 | 9 | 344.33 | 5.00E-11 |
| lnc-GCNT1-4:3 | 9 | 340.53 | 3.56E-11 |
| lnc-GCNT1-4:4 | 9 | 339.16 | 1.37E-10 |
| lnc-ADCY10-1:1 | 1 | 271.06 | 5.67E-05 |
| lnc-UBC-3:1 | 12 | 237.37 | 8.01E-08 |
| lnc-TRIM27-18:1 | 6 | 172.10 | 6.46E-05 |
| LINC00273:13 | 16 | 93.41 | 2.58E-02 |
| lnc-HIST1H2BI-3:1 | 6 | 77.91 | 1.83E-06 |
| lnc-FCGR3A-3:1 | 1 | 55.79 | 3.00E-07 |
| lnc-C6orf100-12:1 | 6 | 36.47 | 3.60E-07 |
| lnc-FNBP1L-2:1 | 1 | 32.21 | 4.28E-04 |
| lnc-GNGT2-1:2 | 17 | 29.44 | 5.09E-07 |
| lnc-PRRC2C-5:1 | 1 | 27.81 | 7.48E-06 |
| lnc-MAP1LC3B-7:1 | 16 | 25.24 | 1.67E-06 |
| LINC00273:8 | 16 | 23.88 | 1.08E-03 |
| GAS5:78 | 1 | 22.92 | 2.14E-03 |
| SNHG1:57 | 11 | 18.09 | 1.29E-03 |
| lnc-ZNF391-5:2 | 6 | 17.09 | 7.98E-04 |
| lnc-HIST1H2BI-2:2 | 6 | 15.01 | 2.42E-05 |
| lnc-NARF-2:2 | 17 | 14.87 | 2.30E-05 |
| lnc-EPHA7-8:1 | 6 | 14.12 | 3.90E-04 |
| lnc-AC102948.1.1-4:1 | 17 | 13.05 | 3.09E-04 |
| lnc-C15orf52-3:3 | 15 | 12.22 | 5.96E-03 |
| lnc-TRIM7-2:5 | 5 | 12.14 | 7.84E-05 |
| lnc-TSPAN11-10:1 | 12 | 12.09 | 1.37E-03 |
| LIMD1-AS1:6 | 3 | 11.23 | 1.42E-05 |
| lnc-PARVG-4:1 | 22 | 10.21 | 2.02E-04 |
| lnc-CLP1-3:11 | 11 | 9.65 | 8.50E-04 |
| lnc-IL17RB-3:1 | 3 | 8.72 | 1.58E-03 |
| lnc-FANCI-1:1 | 15 | 8.41 | 6.52E-03 |
| lnc-HFE2-1:1 | 1 | 8.05 | 2.01E-03 |
| lnc-OR2V2-3:1 | 5 | 7.58 | 1.14E-02 |
| lnc-SDHC-3:1 | 1 | 7.45 | 1.45E-03 |
| lnc-ZNF133-3:1 | 20 | 7.41 | 9.60E-04 |
| lnc-TRIM27-10:2 | 6 | 6.42 | 1.01E-02 |
| MIR17HG:8 | 13 | 6.26 | 1.69E-02 |
| lnc-ZNF391-5:4 | 6 | 6.04 | 6.72E-04 |
| lnc-AL669831.1-11:4 | 1 | 5.93 | 5.18E-03 |
| lnc-TMPO-1:1 | 12 | 5.88 | 9.59E-04 |
| lnc-OR4D9-1:1 | 11 | 5.20 | 2.19E-02 |
| lnc-MDM4-11:1 | 1 | 4.82 | 1.40E-02 |
| lnc-OSBP-1:4 | 11 | 4.38 | 8.31E-03 |
| LINC01623:6 | 6 | 4.22 | 3.98E-02 |
| lnc-SAG-4:1 | 2 | 3.99 | 1.14E-04 |
| lnc-C1orf132-1:6 | 1 | 3.93 | 1.34E-02 |
| MIR22HG:24 | 17 | 3.38 | 5.90E-03 |
| MIR22HG:18 | 17 | 3.37 | 5.68E-03 |
| MIR22HG:35 | 17 | 3.37 | 5.68E-03 |
| MIR22HG:47 | 17 | 3.33 | 5.85E-03 |
| lnc-ZNF649-2:1 | 19 | 3.10 | 2.65E-02 |
| lnc-KDM4C-18:1 | 9 | -1.66 | 4.15E-02 |
| lnc-RP4-695O20__B.10.1-3:10 | 22 | -1.73 | 4.58E-02 |
| lnc-RP4-695O20__B.10.1-3:12 | 22 | -1.73 | 4.56E-02 |
| lnc-RP4-695O20__B.10.1-3:9 | 22 | -1.73 | 4.56E-02 |
| lnc-MON2-2:8 | 12 | -1.75 | 3.44E-02 |
| lnc-MON2-2:10 | 12 | -1.75 | 3.31E-02 |
| lnc-MON2-2:2 | 12 | -1.76 | 3.31E-02 |
| lnc-CUL1-5:1 | 7 | -1.80 | 4.52E-02 |
| lnc-RRBP1-3:1 | 20 | -1.86 | 6.68E-03 |
| lnc-LRR1-1:4 | 14 | -2.18 | 1.55E-02 |
| lnc-RPL13A-1:5 | 19 | -2.19 | 5.58E-03 |
| lnc-LRR1-1:1 | 14 | -2.21 | 1.50E-02 |
| DNM3OS:3 | 1 | -2.24 | 1.69E-02 |
| lnc-C19orf57-6:5 | 19 | -2.26 | 1.45E-02 |
| lnc-HARS-1:1 | 5 | -2.30 | 2.31E-02 |
| lnc-FGA-1:1 | 4 | -2.31 | 7.44E-03 |
| lnc-TRIM52-2:1 | 5 | -2.34 | 5.18E-03 |
| lnc-SNAPC5-4:3 | 15 | -2.39 | 8.63E-03 |
| lnc-DAO-3:1 | 12 | -2.41 | 4.04E-03 |
| lnc-SLC15A4-24:1 | 12 | -2.46 | 3.06E-03 |
| lnc-ANKRD34B-4:2 | 5 | -2.46 | 4.60E-03 |
| lnc-SYT10-3:5 | 12 | -2.53 | 8.46E-04 |
| SNHG16:42 | 17 | -2.58 | 9.23E-04 |
| lnc-ZNF169-7:7 | 9 | -2.58 | 1.95E-02 |
| MIR210HG:7 | 11 | -2.64 | 5.97E-03 |
| lnc-KNG1-2:8 | 3 | -2.70 | 1.25E-02 |
| lnc-AL031590.1-1:2 | 22 | -2.72 | 2.42E-04 |
| lnc-GMEB1-1:5 | 1 | -2.75 | 2.05E-02 |
| lnc-AL669831.1-12:2 | 1 | -2.75 | 9.95E-04 |
| SNHG22:3 | 18 | -2.87 | 1.53E-03 |
| lnc-CFH-2:1 | 1 | -2.88 | 3.08E-03 |
| lnc-ZNF169-7:2 | 9 | -2.90 | 4.17E-03 |
| SNHG1:4 | 11 | -2.91 | 1.34E-02 |
| SNHG1:44 | 11 | -2.91 | 1.34E-02 |
| LINC00273:1 | 16 | -2.93 | 7.48E-04 |
| lnc-FGG-2:1 | 4 | -3.01 | 1.78E-03 |
| MIR193BHG:18 | 16 | -3.06 | 1.41E-02 |
| lnc-C11orf54-1:1 | 11 | -3.06 | 1.04E-03 |
| lnc-CTD-2144E22.5.1-20:1 | 16 | -3.13 | 2.44E-02 |
| lnc-TMC2-1:1 | 20 | -3.13 | 7.18E-04 |
| lnc-AL669831.1-14:1 | 1 | -3.14 | 4.54E-04 |
| lnc-CTD-2144E22.5.1-19:1 | 16 | -3.21 | 4.63E-03 |
| lnc-NEK8-2:1 | 17 | -3.21 | 8.59E-03 |
| UBA6-AS1:26 | 4 | -3.34 | 1.11E-03 |
| lnc-HNRNPA2B1-10:4 | 7 | -3.38 | 4.55E-03 |
| lnc-MINA-3:5 | 3 | -3.47 | 2.14E-03 |
| lnc-TSPY10-14:1 | Y | -3.51 | 3.31E-04 |
| lnc-COL4A5-3:1 | X | -3.52 | 4.31E-03 |
| lnc-AFP-3:1 | 4 | -3.57 | 3.72E-04 |
| SNHG5:5 | 6 | -3.58 | 1.85E-04 |
| SNHG5:47 | 6 | -3.58 | 1.85E-04 |
| MIR17HG:6 | 13 | -3.65 | 2.00E-03 |
| GAS5:16 | 1 | -3.74 | 2.72E-03 |
| GAS5:68 | 1 | -3.81 | 1.22E-04 |
| ZFAS1:23 | 20 | -3.84 | 8.71E-04 |
| lnc-SLC3A2-6:1 | 11 | -3.85 | 1.30E-02 |
| lnc-C17orf51-5:1 | 17 | -3.92 | 9.98E-03 |
| lnc-AFP-1:2 | 4 | -3.97 | 1.43E-04 |
| lnc-TMEM132C-11:1 | 12 | -4.00 | 1.39E-02 |
| lnc-AC106017.1.1-2:2 | 17 | -4.15 | 1.36E-04 |
| lnc-KIF2C-2:1 | 1 | -4.27 | 7.35E-05 |
| lnc-TRIM59-2:1 | 3 | -4.27 | 1.26E-05 |
| lnc-CRKL-2:1 | 22 | -4.37 | 1.92E-03 |
| lnc-VSTM5-1:10 | 11 | -4.43 | 5.42E-03 |
| lnc-GPR39-10:3 | 2 | -4.50 | 7.66E-06 |
| lnc-SURF2-1:1 | 9 | -4.52 | 3.77E-04 |
| lnc-VSTM5-1:13 | 11 | -4.52 | 4.48E-04 |
| lnc-GPR39-7:1 | 2 | -4.84 | 1.58E-05 |
| lnc-AK3-1:1 | 9 | -5.01 | 8.62E-04 |
| lnc-GCNT1-4:1 | 9 | -5.20 | 4.71E-04 |
| lnc-SNURF-1:2 | 15 | -5.27 | 2.68E-04 |
| lnc-KLK1-2:3 | 19 | -5.31 | 1.80E-04 |
| lnc-RABGGTB-1:6 | 1 | -5.33 | 1.18E-04 |
| lnc-AC006156.1-11:1 | Y | -5.34 | 1.11E-04 |
| lnc-ZBTB37-2:1 | 1 | -5.45 | 6.70E-04 |
| lnc-SPG7-2:3 | 16 | -5.57 | 1.18E-04 |
| lnc-HAUS5-3:2 | 19 | -5.63 | 2.37E-04 |
| lnc-HAUS5-3:1 | 19 | -5.64 | 2.37E-04 |
| lnc-GRAP-1:2 | 17 | -5.77 | 2.03E-02 |
| SNHG19:3 | 16 | -5.83 | 5.93E-07 |
| lnc-APAF1-3:1 | 12 | -5.86 | 1.72E-04 |
| lnc-GPR39-10:2 | 2 | -5.90 | 4.74E-06 |
| lnc-AUTS2-6:1 | 7 | -5.97 | 1.69E-06 |
| lnc-MSH3-2:1 | 5 | -6.00 | 3.22E-04 |
| lnc-SERHL2-4:2 | 22 | -6.05 | 4.99E-03 |
| lnc-SERHL2-4:4 | 22 | -6.05 | 4.99E-03 |
| MIR503HG:16 | X | -6.05 | 2.37E-04 |
| SNHG8:14 | 4 | -6.10 | 1.85E-04 |
| MIR503HG:17 | X | -6.37 | 1.83E-04 |
| MIR99AHG:50 | 21 | -6.38 | 3.01E-05 |
| MIR99AHG:51 | 21 | -6.38 | 3.01E-05 |
| MIR99AHG:42 | 21 | -6.39 | 3.01E-05 |
| MIR99AHG:63 | 21 | -6.39 | 3.01E-05 |
| SNHG1:12 | 11 | -6.60 | 2.37E-04 |
| lnc-AUTS2-6:2 | 7 | -6.64 | 6.46E-05 |
| lnc-AFP-2:1 | 4 | -6.85 | 5.70E-05 |
| lnc-AC007952.2.1-2:2 | 17 | -7.17 | 8.59E-03 |
| lnc-MYO16-7:1 | 13 | -7.23 | 8.16E-06 |
| lnc-MOS-1:2 | 8 | -7.31 | 8.92E-05 |
| lnc-SNAPC5-4:2 | 15 | -7.83 | 6.46E-05 |
| lnc-SNAPC5-4:1 | 15 | -7.84 | 6.51E-05 |
| LINC00910:1 | 17 | -8.32 | 1.05E-03 |
| LINC00910:16 | 17 | -8.37 | 1.05E-03 |
| SNHG1:59 | 11 | -8.58 | 3.01E-05 |
| LRRC75A-AS1:49 | 17 | -9.71 | 7.66E-06 |
| lnc-RPL17-2:4 | 18 | -10.41 | 1.10E-05 |
| LINC01138:11 | 1 | -10.48 | 2.43E-07 |
| SNHG1:25 | 11 | -10.68 | 8.92E-05 |
| MIR17HG:5 | 13 | -11.08 | 3.52E-05 |
| lnc-SNURF-1:92 | 15 | -11.71 | 7.72E-06 |
| lnc-SNURF-1:95 | 15 | -11.71 | 7.48E-06 |
| lnc-VSTM5-1:7 | 11 | -12.65 | 1.41E-05 |
| SNHG1:1 | 11 | -13.09 | 6.16E-06 |
| GAS5:43 | 1 | -16.72 | 1.65E-05 |
| lnc-MINA-3:4 | 3 | -17.25 | 1.19E-06 |
| lnc-NEDD4L-1:6 | 18 | -20.51 | 3.13E-06 |
| lnc-TPTE-3:5 | 21 | -26.36 | 1.72E-04 |
| LRRC75A-AS1:41 | 17 | -27.36 | 6.51E-05 |
| lnc-ARHGEF6-4:1 | X | -27.80 | 3.84E-09 |
| LRRC75A-AS1:36 | 17 | -36.34 | 1.17E-07 |
| lnc-CCNB1IP1-1:2 | 14 | -72.25 | 1.00E-04 |
| GAS5:41 | 1 | -89.48 | 4.10E-04 |
| lnc-HSD17B10-3:1 | X | -103.78 | 1.18E-04 |
| GAS5:72 | 1 | -124.14 | 2.34E-06 |
| GAS5:7 | 1 | -124.14 | 2.34E-06 |
| SNHG6:15 | 8 | -144.53 | 3.38E-05 |
| lnc-AC022098.1-1:10 | 19 | -756.02 | 4.53E-06 |

**Table 2: Differentially expressed lncRNAs in hepatocellular carcinoma vs healthy patient’s tissue samples (FDR <0.05)**

| **lncRNA ID** | **Chromosome** | **Fold change** | **FDR** |
| --- | --- | --- | --- |
| lnc-CCDC167-2:2 | 6 | 1000000.00 | 5.17E-21 |
| lnc-TPTE-3:3 | 21 | 18378.09 | 1.01E-08 |
| lnc-C21orf67-10:1 | 21 | 9271.37 | 9.93E-13 |
| lnc-TMEM8A-1:4 | 16 | 6308.60 | 3.57E-15 |
| lnc-CRK-3:2 | 17 | 3027.08 | 1.45E-10 |
| lnc-FBXO11-7:1 | 2 | 2400.27 | 5.65E-13 |
| lnc-SHISA5-1:6 | 3 | 1043.39 | 1.39E-10 |
| MYLK-AS1:12 | 3 | 553.44 | 2.41E-09 |
| lnc-GCNT1-4:6 | 9 | 490.49 | 3.54E-20 |
| lnc-GCNT1-4:5 | 9 | 418.47 | 1.42E-18 |
| lnc-GCNT1-4:3 | 9 | 413.33 | 1.33E-14 |
| lnc-GCNT1-4:2 | 9 | 400.03 | 2.33E-14 |
| lnc-GCNT1-4:4 | 9 | 395.76 | 1.29E-13 |
| lnc-LRRC48-2:1 | 17 | 303.29 | 1.48E-08 |
| LINC00273:13 | 16 | 261.58 | 3.25E-03 |
| lnc-ADCY10-1:1 | 1 | 245.11 | 6.15E-08 |
| lnc-UBC-3:1 | 12 | 243.81 | 1.53E-12 |
| lnc-TRIM27-18:1 | 6 | 211.08 | 4.17E-08 |
| HAGLR:1 | 2 | 140.96 | 6.73E-05 |
| HAGLR:31 | 2 | 140.87 | 6.73E-05 |
| lnc-FCGR3A-3:1 | 1 | 51.63 | 1.47E-14 |
| lnc-HIST1H2BI-3:1 | 6 | 51.19 | 1.20E-09 |
| GAS5:78 | 1 | 44.83 | 2.60E-04 |
| lnc-C6orf100-12:1 | 6 | 34.47 | 2.02E-13 |
| lnc-AC106017.1.1-2:1 | 17 | 32.76 | 9.93E-13 |
| lnc-MTA3-6:1 | 2 | 31.78 | 3.46E-13 |
| LINC00273:8 | 16 | 28.40 | 6.55E-07 |
| lnc-RPS16-3:1 | 19 | 27.39 | 1.93E-11 |
| lnc-FNBP1L-2:1 | 1 | 26.04 | 2.20E-05 |
| lnc-MAP1LC3B-7:1 | 16 | 25.62 | 3.94E-10 |
| lnc-GNGT2-1:2 | 17 | 22.97 | 3.46E-11 |
| lnc-PPIAL4C-3:6 | 1 | 20.55 | 5.39E-09 |
| lnc-OR6S1-5:3 | 14 | 19.94 | 7.67E-09 |
| lnc-CHST10-3:1 | 2 | 19.37 | 1.23E-04 |
| lnc-CD226-5:1 | 18 | 19.24 | 1.94E-04 |
| lnc-PRRC2C-5:1 | 1 | 19.11 | 1.47E-09 |
| LINC00869:14 | 1 | 19.02 | 3.09E-06 |
| lnc-EPHA7-8:1 | 6 | 18.16 | 4.73E-06 |
| lnc-SCAND3-2:3 | 6 | 17.27 | 4.53E-07 |
| lnc-TRIM7-2:5 | 5 | 16.30 | 1.23E-05 |
| lnc-PPIL2-2:1 | 22 | 15.86 | 4.73E-06 |
| lnc-ZNF391-5:2 | 6 | 15.77 | 1.59E-04 |
| SNHG3:5 | 1 | 14.25 | 1.89E-06 |
| lnc-C15orf52-3:3 | 15 | 13.67 | 2.42E-05 |
| lnc-TSPAN11-10:1 | 12 | 13.66 | 3.66E-04 |
| lnc-SDHC-3:1 | 1 | 13.40 | 8.66E-04 |
| lnc-HIST1H2BI-2:2 | 6 | 13.05 | 3.27E-08 |
| lnc-NARF-2:2 | 17 | 12.90 | 3.76E-08 |
| SNHG1:63 | 11 | 12.29 | 1.13E-04 |
| LIMD1-AS1:6 | 3 | 10.82 | 7.78E-08 |
| lnc-FANCI-1:4 | 15 | 8.61 | 8.44E-06 |
| lnc-FANCI-1:1 | 15 | 8.61 | 8.44E-06 |
| lnc-FANCI-1:10 | 15 | 8.61 | 8.44E-06 |
| lnc-OR2V2-3:1 | 5 | 7.77 | 1.90E-03 |
| lnc-ECHDC1-1:1 | 6 | 7.73 | 3.33E-02 |
| lnc-AC102948.1.1-4:1 | 17 | 7.00 | 7.40E-04 |
| lnc-PARVG-4:1 | 22 | 6.97 | 1.12E-05 |
| lnc-AP4E1-3:1 | 15 | 6.79 | 2.23E-05 |
| lnc-IL17RB-3:1 | 3 | 6.51 | 4.50E-05 |
| lnc-ZNF391-5:4 | 6 | 6.20 | 2.40E-07 |
| lnc-TRIM7-2:4 | 5 | 6.19 | 1.22E-04 |
| lnc-TRIM27-10:2 | 6 | 6.18 | 4.39E-05 |
| lnc-ZNF133-3:1 | 20 | 5.91 | 4.15E-05 |
| MIR17HG:8 | 13 | 5.88 | 1.49E-03 |
| lnc-HFE2-1:1 | 1 | 5.45 | 5.58E-04 |
| lnc-CLP1-3:11 | 11 | 5.41 | 2.13E-02 |
| SNHG1:34 | 11 | 5.37 | 9.74E-04 |
| lnc-TMPO-1:1 | 12 | 4.88 | 7.07E-05 |
| lnc-AC108134.2-2:1 | 16 | 4.88 | 8.26E-03 |
| lnc-SAG-4:1 | 2 | 4.51 | 2.44E-06 |
| LINC01623:6 | 6 | 4.49 | 1.24E-03 |
| DLEU2:20 | 13 | 4.21 | 4.37E-02 |
| lnc-C1orf132-1:6 | 1 | 3.95 | 6.80E-03 |
| lnc-OR4D9-1:1 | 11 | 3.81 | 4.63E-04 |
| lnc-OSBP-1:4 | 11 | 3.55 | 7.38E-05 |
| lnc-MDM4-11:1 | 1 | 3.43 | 3.67E-04 |
| lnc-C3orf25-2:15 | 3 | 3.28 | 4.59E-04 |
| GAS5:2 | 1 | 3.11 | 4.66E-04 |
| DANCR:14 | 4 | 3.08 | 8.62E-04 |
| lnc-IL6-2:11 | 7 | 2.95 | 2.34E-02 |
| lnc-AC007952.2.1-2:1 | 17 | 2.86 | 1.44E-04 |
| lnc-GRAP-1:1 | 17 | 2.84 | 2.21E-04 |
| lnc-AC007952.1.1-3:1 | 17 | 2.75 | 1.80E-04 |
| lnc-AC007952.2-2:3 | 17 | 2.70 | 3.36E-04 |
| lnc-AC007952.2-2:1 | 17 | 2.69 | 3.62E-04 |
| lnc-METTL12-1:2 | 11 | 2.66 | 4.59E-04 |
| MIR22HG:24 | 17 | 2.57 | 5.14E-04 |
| MIR22HG:47 | 17 | 2.57 | 5.14E-04 |
| MIR22HG:35 | 17 | 2.56 | 5.25E-04 |
| MIR22HG:18 | 17 | 2.56 | 5.25E-04 |
| lnc-AL669831.1-11:4 | 1 | 2.53 | 2.21E-02 |
| lnc-WFIKKN1-1:2 | 16 | 2.46 | 1.05E-02 |
| MIR193BHG:21 | 16 | 2.31 | 6.95E-03 |
| lnc-ZNF649-2:1 | 19 | 2.23 | 2.34E-02 |
| lnc-TMEM167B-1:5 | 1 | 1.83 | 4.98E-03 |
| lnc-TMEM167B-1:4 | 1 | 1.70 | 1.06E-02 |
| MIR3142HG:2 | 5 | -1.59 | 4.08E-02 |
| lnc-MINA-3:1 | 3 | -1.68 | 4.74E-02 |
| lnc-CTD-2144E22.5.1-20:1 | 16 | -1.69 | 1.82E-02 |
| lnc-DAO-3:1 | 12 | -1.71 | 1.88E-02 |
| lnc-TMC2-1:1 | 20 | -1.77 | 1.81E-02 |
| lnc-AL031590.1-1:2 | 22 | -1.82 | 2.75E-03 |
| lnc-TRIM52-2:1 | 5 | -1.83 | 1.13E-02 |
| lnc-KLK1-2:3 | 19 | -1.84 | 3.19E-02 |
| lnc-RPS13-1:6 | 11 | -1.85 | 5.40E-03 |
| SNHG22:3 | 18 | -1.87 | 6.20E-03 |
| lnc-C19orf57-6:5 | 19 | -1.87 | 3.77E-02 |
| lnc-ANKRD34B-4:2 | 5 | -1.88 | 9.24E-03 |
| lnc-SYT10-3:5 | 12 | -1.91 | 9.27E-03 |
| lnc-CRKL-2:1 | 22 | -1.97 | 3.41E-02 |
| MIR193BHG:18 | 16 | -1.98 | 4.88E-02 |
| lnc-MON2-2:8 | 12 | -1.98 | 9.63E-03 |
| lnc-MON2-2:10 | 12 | -1.99 | 8.91E-03 |
| lnc-MON2-2:2 | 12 | -2.00 | 8.92E-03 |
| lnc-SLC15A4-24:1 | 12 | -2.00 | 9.36E-03 |
| SNHG16:42 | 17 | -2.01 | 1.66E-03 |
| lnc-SNAPC5-4:3 | 15 | -2.10 | 4.99E-03 |
| UBA6-AS1:26 | 4 | -2.12 | 3.02E-03 |
| lnc-ZNF860-1:1 | 3 | -2.14 | 7.49E-04 |
| lnc-HARS-1:1 | 5 | -2.19 | 1.08E-02 |
| lnc-TSPY10-14:1 | Y | -2.21 | 1.53E-03 |
| lnc-CUL1-5:1 | 7 | -2.22 | 4.08E-03 |
| GAS5:16 | 1 | -2.28 | 2.23E-02 |
| lnc-NEK8-2:1 | 17 | -2.30 | 1.62E-02 |
| lnc-HEPH-1:1 | X | -2.46 | 1.24E-02 |
| lnc-ZBTB37-2:1 | 1 | -2.49 | 2.23E-02 |
| lnc-RP4-695O20__B.10.1-3:10 | 22 | -2.50 | 6.46E-03 |
| lnc-RP4-695O20__B.10.1-3:12 | 22 | -2.50 | 6.46E-03 |
| lnc-RP4-695O20__B.10.1-3:9 | 22 | -2.50 | 6.46E-03 |
| LINC00472:10 | 6 | -2.51 | 1.17E-02 |
| lnc-C11orf54-1:1 | 11 | -2.54 | 4.85E-04 |
| lnc-MINA-3:5 | 3 | -2.57 | 2.37E-03 |
| lnc-COL4A5-3:1 | X | -2.62 | 3.65E-03 |
| lnc-SNURF-1:73 | 15 | -2.65 | 3.29E-02 |
| lnc-AL669831.1-14:1 | 1 | -2.70 | 2.29E-04 |
| lnc-CFH-2:1 | 1 | -2.70 | 1.94E-03 |
| lnc-AC106017.1.1-2:2 | 17 | -2.74 | 7.36E-05 |
| lnc-CPNE4-2:1 | 3 | -2.79 | 7.34E-04 |
| lnc-VSTM5-1:10 | 11 | -2.80 | 8.40E-03 |
| MALAT1:10 | 11 | -2.86 | 2.75E-03 |
| ZFAS1:23 | 20 | -2.89 | 1.25E-03 |
| LINC00324:3 | 17 | -2.92 | 6.15E-03 |
| LINC00273:1 | 16 | -2.94 | 1.35E-04 |
| GAS5:68 | 1 | -3.08 | 3.01E-05 |
| lnc-FBXO9-1:2 | 6 | -3.11 | 4.05E-04 |
| lnc-FBXO9-1:1 | 6 | -3.11 | 4.05E-04 |
| DNM3OS:3 | 1 | -3.15 | 4.90E-03 |
| lnc-SURF2-1:1 | 9 | -3.17 | 6.46E-04 |
| lnc-VSTM5-1:13 | 11 | -3.27 | 7.80E-04 |
| lnc-SLC3A2-6:1 | 11 | -3.30 | 5.37E-03 |
| lnc-FGA-1:1 | 4 | -3.31 | 6.99E-05 |
| SNHG5:5 | 6 | -3.35 | 5.03E-06 |
| SNHG5:47 | 6 | -3.35 | 5.03E-06 |
| MIR17HG:6 | 13 | -3.38 | 1.45E-03 |
| lnc-GPR39-10:3 | 2 | -3.64 | 9.51E-07 |
| lnc-GPR39-7:1 | 2 | -3.68 | 6.16E-06 |
| lnc-GCNT1-4:1 | 9 | -3.70 | 4.16E-04 |
| lnc-HAUS5-3:1 | 19 | -3.73 | 3.36E-04 |
| lnc-TMEM132C-11:1 | 12 | -3.73 | 1.26E-02 |
| lnc-HAUS5-3:2 | 19 | -3.75 | 3.15E-04 |
| lnc-TRIM59-2:1 | 3 | -3.97 | 1.31E-07 |
| lnc-SERHL2-4:2 | 22 | -4.01 | 5.80E-03 |
| lnc-SERHL2-4:4 | 22 | -4.01 | 5.80E-03 |
| lnc-HNRNPA2B1-10:4 | 7 | -4.03 | 4.73E-05 |
| lnc-KIF2C-2:1 | 1 | -4.04 | 1.70E-06 |
| lnc-GRAP-1:2 | 17 | -4.07 | 1.38E-02 |
| lnc-MSH3-2:1 | 5 | -4.10 | 1.27E-04 |
| lnc-AL669831.1-12:2 | 1 | -4.16 | 1.02E-06 |
| lnc-RABGGTB-1:6 | 1 | -4.16 | 7.01E-06 |
| lnc-AUTS2-6:1 | 7 | -4.43 | 1.89E-06 |
| lnc-FGG-2:1 | 4 | -4.43 | 2.15E-06 |
| lnc-ERAL1-1:1 | 17 | -4.55 | 2.48E-03 |
| lnc-ZNF169-7:2 | 9 | -4.58 | 2.66E-04 |
| lnc-DNAJC12-1:1 | 10 | -4.60 | 2.12E-02 |
| SNHG8:14 | 4 | -4.64 | 2.10E-04 |
| lnc-GPR39-10:2 | 2 | -4.69 | 3.16E-07 |
| lnc-AC006156.1-11:1 | Y | -4.71 | 1.55E-05 |
| lnc-AC007952.2.1-2:2 | 17 | -4.73 | 9.24E-03 |
| lnc-SPG7-2:3 | 16 | -4.74 | 3.77E-05 |
| lnc-MYO16-7:1 | 13 | -4.86 | 6.65E-06 |
| lnc-APAF1-3:1 | 12 | -4.88 | 8.13E-06 |
| SNHG19:3 | 16 | -4.98 | 6.12E-09 |
| MIR210HG:7 | 11 | -5.18 | 7.86E-06 |
| lnc-TPTE-3:9 | 21 | -5.19 | 4.80E-02 |
| lnc-AUTS2-6:2 | 7 | -5.25 | 2.51E-05 |
| lnc-SNURF-1:2 | 15 | -5.33 | 4.30E-05 |
| lnc-MOS-1:2 | 8 | -5.53 | 8.44E-06 |
| SNHG1:1 | 11 | -6.34 | 8.73E-06 |
| LRRC75A-AS1:49 | 17 | -6.54 | 3.59E-05 |
| SNHG1:12 | 11 | -6.61 | 9.41E-04 |
| lnc-TTLL10-3:9 | 1 | -6.67 | 1.08E-04 |
| MIR503HG:16 | X | -7.26 | 7.81E-05 |
| MIR503HG:17 | X | -7.48 | 6.02E-05 |
| GAS5:43 | 1 | -7.96 | 4.53E-05 |
| LINC01138:11 | 1 | -8.01 | 4.93E-10 |
| lnc-RPL17-2:4 | 18 | -8.13 | 1.48E-06 |
| lnc-MINA-3:4 | 3 | -8.69 | 8.51E-06 |
| lnc-AFP-3:1 | 4 | -8.82 | 1.95E-10 |
| lnc-AK3-1:1 | 9 | -8.92 | 5.62E-06 |
| MIR99AHG:51 | 21 | -9.39 | 6.57E-09 |
| MIR99AHG:50 | 21 | -9.39 | 6.57E-09 |
| MIR99AHG:42 | 21 | -9.40 | 6.57E-09 |
| MIR99AHG:63 | 21 | -9.41 | 6.57E-09 |
| MIR17HG:5 | 13 | -9.54 | 1.03E-04 |
| ARHGAP5-AS1:1 | 14 | -9.70 | 7.50E-09 |
| SNHG1:59 | 11 | -9.91 | 2.12E-08 |
| lnc-AFP-1:2 | 4 | -10.35 | 3.44E-10 |
| lnc-SNAPC5-4:2 | 15 | -10.85 | 5.99E-09 |
| lnc-SNAPC5-4:1 | 15 | -10.91 | 6.40E-09 |
| LINC00910:1 | 17 | -11.64 | 1.11E-05 |
| LINC00910:16 | 17 | -11.71 | 1.11E-05 |
| lnc-VSTM5-1:7 | 11 | -11.82 | 1.63E-06 |
| lnc-TPTE-3:5 | 21 | -13.47 | 2.44E-03 |
| lnc-SNURF-1:92 | 15 | -13.54 | 3.85E-08 |
| lnc-SNURF-1:95 | 15 | -13.54 | 3.80E-08 |
| LRRC75A-AS1:41 | 17 | -15.93 | 8.76E-06 |
| lnc-AFP-2:1 | 4 | -16.47 | 2.78E-10 |
| LRRC75A-AS1:36 | 17 | -27.87 | 5.21E-10 |
| lnc-NEDD4L-1:6 | 18 | -28.32 | 6.04E-11 |
| lnc-ARHGEF6-4:1 | X | -28.40 | 3.41E-15 |
| GAS5:72 | 1 | -45.29 | 6.27E-06 |
| GAS5:7 | 1 | -45.29 | 6.27E-06 |
| lnc-CCNB1IP1-1:2 | 14 | -50.22 | 7.84E-06 |
| lnc-HSD17B10-3:1 | X | -65.41 | 3.09E-06 |
| SNHG6:15 | 8 | -99.14 | 4.74E-05 |
